# Gram-scale synthesis of a covalent nanocage that preserves the redox properties of encapsulated fullerenes†

**DOI:** 10.1039/d2sc00445c

**Published:** 2022-04-13

**Authors:** Daniel A. Rothschild, William P. Kopcha, Aaron Tran, Jianyuan Zhang, Mark C. Lipke

**Affiliations:** Department of Chemistry and Chemical Biology, Rutgers, The State University of New Jersey 123 Bevier Rd Piscataway NJ 08854 USA ml1353@chem.rutgers.edu

## Abstract

Discrete nanocages provide a way to solubilize, separate, and tune the properties of fullerenes, but these 3D receptors cannot usually be synthesized easily from inexpensive starting materials, limiting their utility. Herein, we describe the first fullerene-binding nanocage (Cage^4+^) that can be made efficiently on a gram scale. Cage^4+^ was prepared in up to 57% yield by the formation of pyridinium linkages between complemantary porphyrin components that are themselves readily accessible. Cage^4+^ binds C_60_ and C_70_ with large association constants (>10^8^ M^−1^), thereby solubilizing these fullerenes in polar solvents. Fullerene association and redox-properties were subsequently investigated across multiple charge states of the host-guest complexes. Remarkably, neutral and singly reduced fullerenes bind with similar strengths, leaving their 0/1^−^ redox couples minimally perturbed and fully reversible, whereas other hosts substantially alter the redox properties of fullerenes. Thus, C_60_@Cage^4+^ and C_70_@Cage^4+^ may be useful as solubilized fullerene derivatives that preserve the inherent electron-accepting and electron-transfer capabilities of the fullerenes. Fulleride dianions were also found to bind strongly in Cage^4+^, while further reduction is centered on the host, leading to lowered association of the fulleride guest in the case of C_60_^2−^.

## Abstract 2

This report describes the first gram-scale synthesis of a nanocage that can host fullerenes (C_60_ and C_70_). The redox properties of the fullerenes are preserved in this host, enabling characterization of complexes with fulleride anions and dianions.

## Graphical abstract

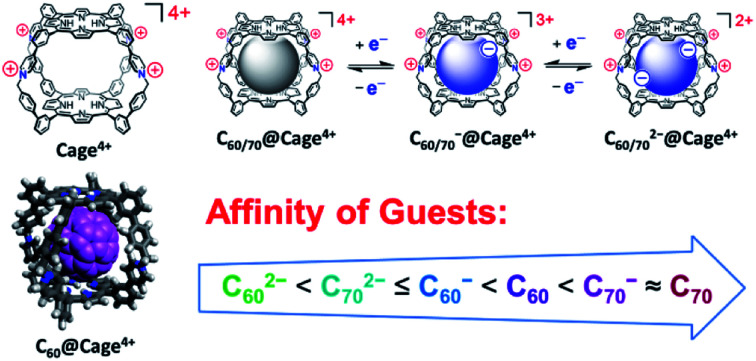

## Introduction

Macrocycles and nanocages with aromatic walls are popular synthetic targets for their interesting structures and ability to host aromatic guests.^1^ Many such molecular receptors have been examined for use in separating,^2^ sensing,^3^ or tuning the electronics^4^ and/or reactivity^5^ of various aromatic species. Fullerenes are one notable class of aromatic guests owing to their useful electron-accepting properties^6^ and the challenges that exist in purifying,^2*a*,7^ solubilizing,^8^ and derivatizing^9^ these aromatic carbon allotropes—challenges which might be overcome using the host-guest chemistry of suitable receptors. Nanocages with large aromatic components (*e.g.*, porphyrins,^2*d*,*f*,4*a*,10^ naphthalene or perylene diimides,^8*a*,11^ pyrenes,^12^ anthracenes,^13^*etc.*^14^) often show particularly high affinities for binding fullerenes due to the large π–π overlap provided upon complexation. However, the synthesis of such hosts is typically challenging or costly, either relying on the stoichiometric use of precious metals to link the aromatic walls‡‡For fullerene receptors utilizing precious metals as linking agents for aromatic walls, see ref. 2*a*, 3*a*, 7*a*, 8*a*, 8*b*, 12*a*, 12*b*, 13*a*, 13*b*, 13*d*, 13*f*, 14*h*, 14*i*, and 14*m*. or utilizing covalent linkages§§For fullerene receptors formed using covalent linkages, see ref. 10*c*, 10*b*, 10*d*, 10*i*, 10*j*, 10*k*, 11*a*, 14*a*, 14*b*, 14*e*, 14*f*, 14*k*, 14*l*, 14*n* and 14*o*. that result in low yields and challenging purifications. Furthermore, the individual components of these hosts often require numerous steps to synthesize,¶¶For fullerene receptors requiring numerous synthetic steps, see ref. 4*d*, 7*a*, 10*a*, 10*d*, 10*e*, 10*f*, 10*i*, 10*j*, 14*f*, 14*j*, and 14*k*. compounding the inefficiency of cage formation.

**Scheme 1:**
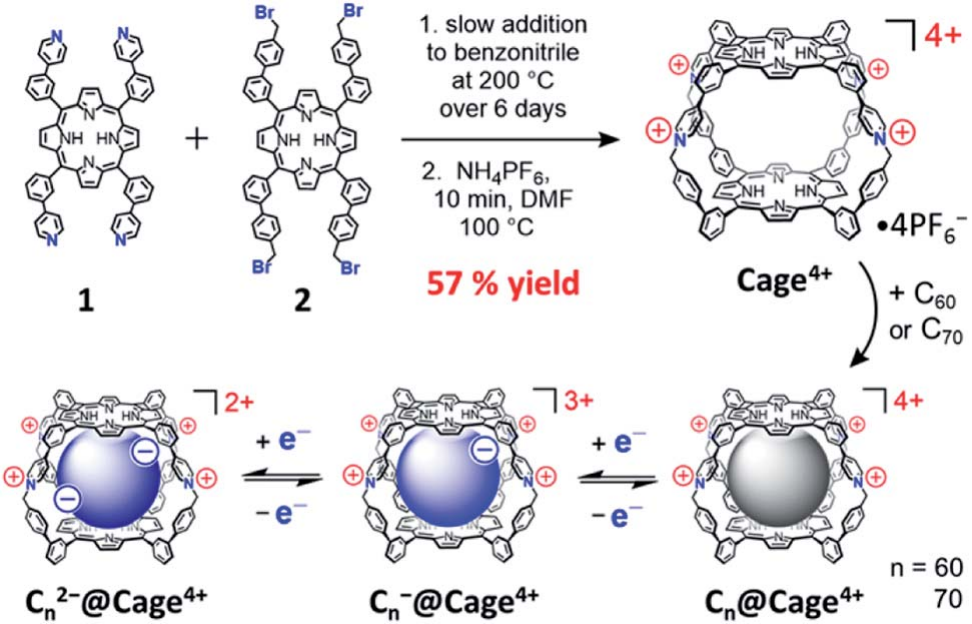
Preparation of Cage^4+^ and complexes with fullerenes and fullerides.

To overcome these limitations, we sought to develop an easily synthesized porphyrin nanocage of the appropriate shape and rigidity to bind fullerenes with high affinities and selectivity. Herein, we report the synthesis, characterization, and fullerene-binding properties of a tetracationic bis-porphyrin nanocage, Cage^4+^, which was prepared in good yield on a gram scale by the formation of pyridinium linkages between complementary pyridyl- and benzylbromide-substituted porphyrins (Scheme 1). This cage uptakes C_60_ and C_70_ quantitatively in MeCN, with essentially complete selectivity for extracting C_70_ from mixtures containing both fullerenes. Notably, the redox properties of the fullerenes are minimally perturbed by encapsulation in Cage^4+^ (Scheme 1) whereas previously reported hosts significantly alter the potentials and/or reversibility of fullerene redox processes.^4*a*,*c*,*d*,8*a*^ The easy, scalable synthesis of Cage^4+^, its strong binding of fullerenes, and the preserved electronics of fullerene guests suggest that C_60_@Cage^4+^ and C_70_@Cage^4+^ might be directly useful as solubilized fullerene derivatives.

## Results and discussion

### Synthesis of Cage^4+^

As shown in Scheme 1, Cage^4+^ was formed *via* simple S_N_2 reactions between complementary 4-fold-symmetric pyridyl- and benzylbromide-substituted porphyrins 1 and 2. Efficient synthesis of the cage was achieved using pseudo-high-dilution conditions in which the two components were added slowly to a moderate volume of PhCN at 200 °C over 6 days. The initial product Cage·4Br is insoluble in PhCN and DMF, allowing oligomeric byproducts to be removed by washing with DMF. Heating the remaining solid in a DMF solution of NH_4_PF_6_ provided the salt Cage·4PF_6_ as the only soluble product in a yield of up to 57%. The identity of Cage^4+^ was confirmed by a variety of NMR techniques (^1^H, ^13^C{^1^H}, and DOSY) in CD_3_CN and DMSO-d_6_ (see Fig. S13–S16 and S25†), and also by ESI(+)-HRMS characterization (Fig. S33–36†).

**Scheme 2:**
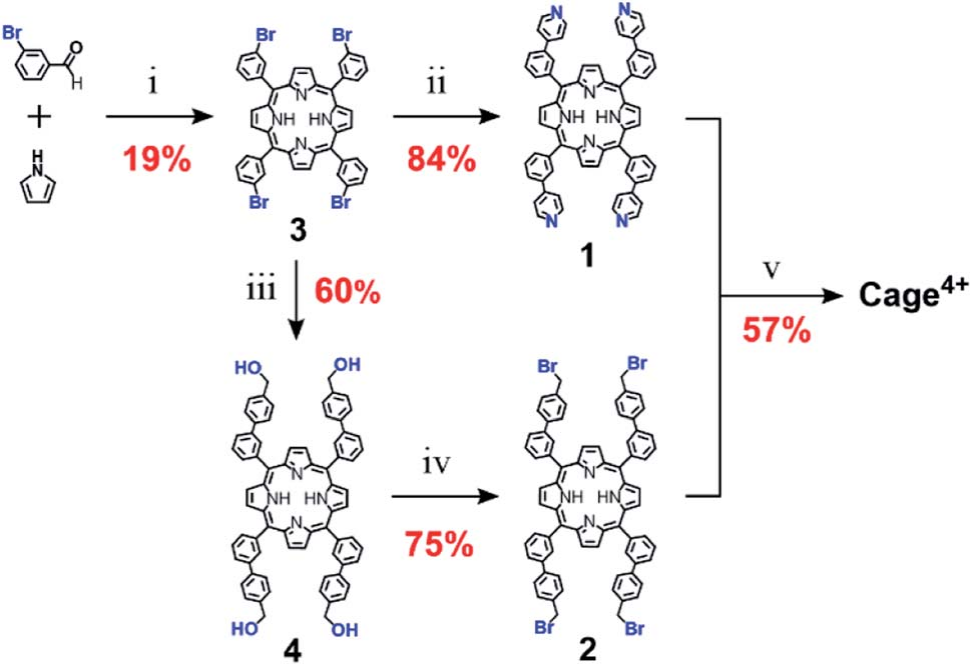
Full synthetic scheme for preparation of Cage^4+^. (i) 160 °C, 2 h, propionic acid (ii) 20 equiv. 4-pyridylboronic acid, 20 equiv. K_2_CO_3_, 12 mol% dppfPdCl_2_, 110 °C, 72 h, 20 : 80 water/toluene, (iii) 20 equiv. 4-(hydroxymethyl)-phenylboronic acid, 20 equiv. K_2_CO_3_, 12 mol% (dppf)PdCl_2_, 110 °C, 72 h, 20/80 water/toluene, (iv) 12 equiv. PBr_3_, 16 h, 0 to 25 °C, CH_2_Cl_2_. (v) 200 °C, 6 days, PhCN, followed by excess NH_4_PF_6_, 100 °C, 10 min, DMF.

The precursors of Cage^4+^ were also prepared easily in only a few steps from inexpensive starting materials (Scheme 2), suggesting the synthesis of Cage^4+^ could be economically scaled. Confirming this possibility, we prepared > 1 g of Cage·4PF_6_ in a single batch, with the percent yield (40%) suffering only a little from scale up. For comparison, we surveyed over 35 reported 3D fullerene receptors,||||We define a 3D receptor as having a well-defined three dimensional pore (see ref. 2*a*, 2*f*, 4*a*, 7*a*, 8*a*, 8*b*, 9*b*, 10*a*, 10*b*, 10*c*, 10*d*, 10*e*, 10*f*, 10*g*, 10*h*, 10*i*, 10*j*, 10*k*, 10*l*, 11*a*, 11*b*, 11*c*, 12*a*, 12*b*, 13*a*, 13*b*, 13*c*, 13*d*, 14*c*, 14*h*, 14*i*, 14*j*, 14*l*, 14*m*, and 14*n*). This definition includes all nanocage structures as well as macrocycles with large aromatic surfaces that define a 3D volume between them, but excludes most molecular tweezers, macrocycles, and linear “wrap-around” hosts that typically show lower association constants for binding fullerenes. Even among these latter receptors, only one has been prepared on a scale of >1 g (see ref. 14*a*). finding that most were prepared on scales of <100 mg, with the largest synthesis providing only about 200 mg of the cage.^12*c*^ To our knowledge, Cage^4+^ is the only fullerene-binding nanocage for which a gram scale synthesis has been demonstrated.

### Association of fullerenes in Cage^4+^

**Fig. 1:**
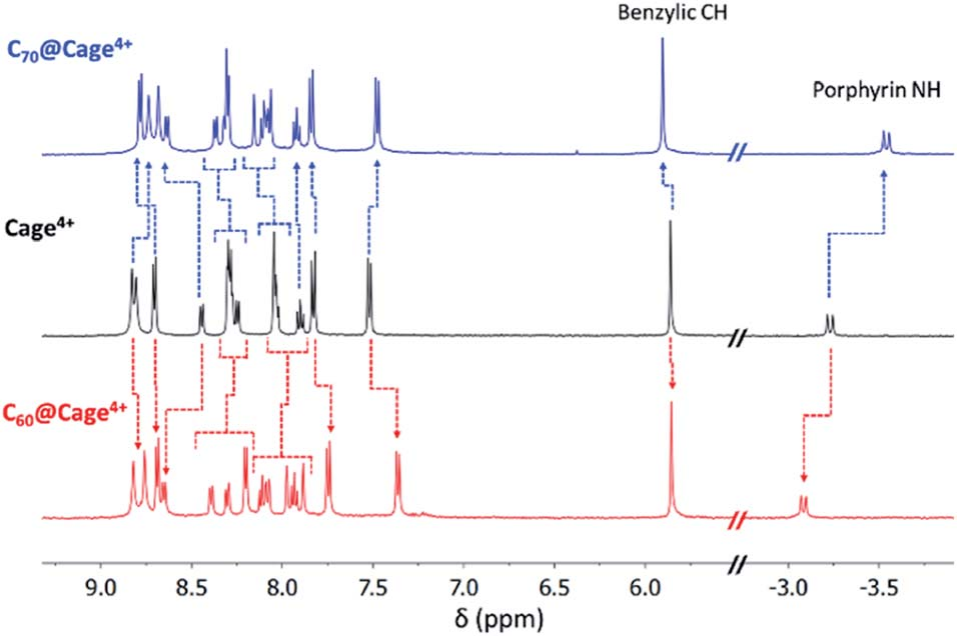
Partial ^1^H NMR spectra of Cage^4+^ (black), C_60_@Cage^4+^ (red), and C_70_@Cage^4+^ (blue) in CD_3_CN (500 MHz, 298 K). Changes to resonances of Cage^4+^ upon guest uptake are labelled with dotted arrows.

Host-guest complexes of C_60_ and C_70_ in Cage^4+^ were formed after 3 h of sonicating suspensions of each fullerene in CD_3_CN solutions of the host. Fullerene encapsulation was evident from changes to all the ^1^H NMR resonances of Cage^4+^ (Fig. 1), with the upfield porphyrin NH signals of the host providing particularly useful NMR handles for tracking complexation. The ^13^C{^1^H} NMR spectra of the host-guest complexes confirm the encapsulation of the fullerenes. Most notably, a large signal at 140.40 ppm was observed for the C_60_ guest in C_60_@Cage^4+^ (Fig. S20†), and five resonances arising from encapsulated C_70_ were observed for C_70_@Cage^4+^ (Fig. S24†), whereas ^13^C NMR signals of C_60_ and C_70_ cannot otherwise be observed in CD_3_CN due to the negligible solubility of these fullerenes in this solvent.^15^ ESI(+)-HRMS characterization further confirmed the identity of the host-guest complexes, showing a series of peaks with the expected isotope patterns for C_60_@Cage^4+^ and C_70_@Cage^4+^ with 0 to 2 PF_6_^−^ anions associated (Fig. S37–S44†).

The strength of association between C_60_ and Cage^4+^ was estimated based on the observation that the empty host could not be detected by ^1^H NMR spectroscopy after sonicating an excess of the fullerene in a saturated (0.65 mM) solution of Cage^4+^ in CD_3_CN (Fig. 1). Using the reported solubility of C_60_ in MeCN (0.56 μM)^15^ and the lower limit of ^1^H NMR detection of Cage^4+^ (1.94 μM), it can be concluded that C_60_@Cage^4+^ has a very large association constant of ≥ 6.0 × 10^8^ M^−1^. It was not possible to measure the binding strength more precisely in MeCN, so the association of C_60_ in Cage^4+^ was also quantified in benzonitrile. Fluorescence measurements revealed that C_60_@Cage^4+^ forms with a *K*_a_ of 1.14 ± 0.30 × 10^7^ M^−1^ in PhCN (Fig. S74†). Since PhCN is orders of magnitude better than MeCN at solvating C_60_,^15^ the fullerene should bind much more strongly in Cage^4+^ in MeCN, supporting the estimate of a *K*_a_ > 10^8^ M^−1^ for the formation of C_60_@Cage^4+^ in MeCN.

Strong binding of C_70_ was also evident in CD_3_CN from complete disappearance of the ^1^H NMR signals of empty Cage^4+^ after sonication in the presence of this fullerene (Fig. 1), but like for C_60_, the association constant could not be quantified precisely in this solvent. Instead, the association constant for C_70_@Cage^4+^ was measured in PhCN (Fig. S75†), revealing a *K*_a_ (1.10 ± 0.17 × 10^8^ M^−1^) that is an order of magnitude higher than that for binding C_60_ in PhCN. Competition experiments confirmed that this preference for binding C_70_ extends to MeCN solvent conditions. Sonication of a 10 : 1 mixture of C_60_ : C_70_ in a solution of Cage^4+^ in CD_3_CN led to complete disappearance of the signals of the free host from the ^1^H NMR spectrum after 3 h, initially providing C_60_@Cage^4+^ and C_70_@Cage^4+^ in similar amounts (see Fig. S30†). However, the ratio of C_70_@Cage^4+^ to C_60_@Cage^4+^ steadily increased upon further sonication, until only the C_70_ complex could be observed by ^1^H NMR spectroscopy after 26 h (Fig. S30†). These observations show that the kinetics of fullerene uptake are similar for both guests, but C_70_@Cage^4+^ is the thermodynamically more favourable complex. Thus, Cage^4+^ is highly effective for separating C_70_ from mixtures of C_60_ and C_70_, showing perfect selectivity within the limits of ^1^H NMR detection (≥30-fold selectivity for C_70_).

### Structural analysis of host-guest complexes

**Fig. 2:**
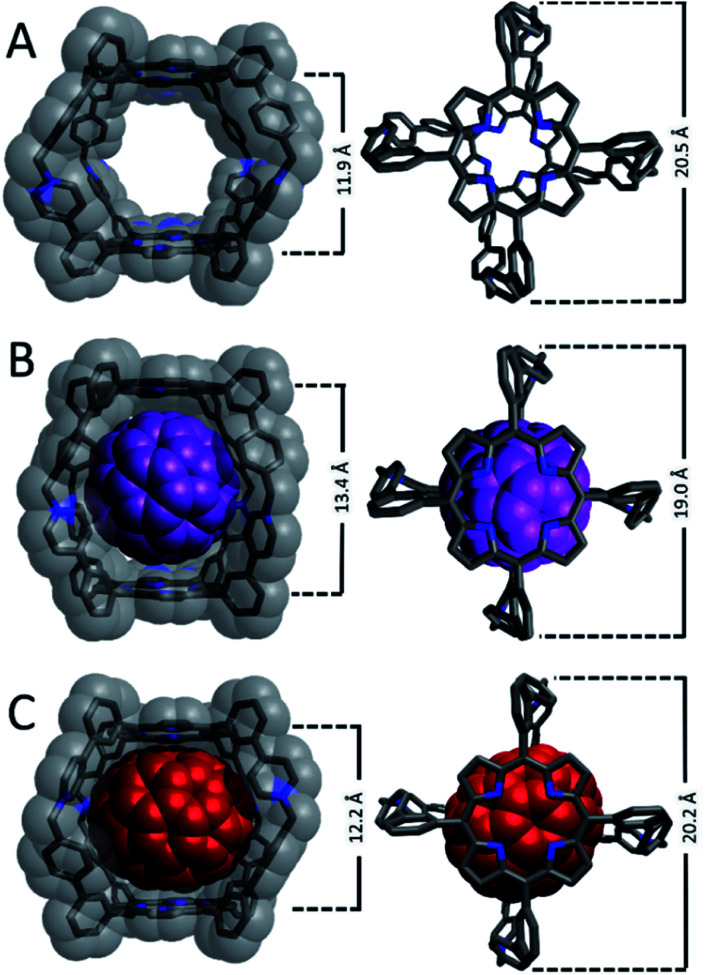
DFT optimized structures of (A) Cage^4+^ (B3LYP/6-31+G(d,p)), (B) C_60_@Cage^4+^ (B3LYP/3-21+G), and (C) C_70_@Cage^4+^ (B3LYP/3-21+G). All optimizations were performed using an SMD solvation model for acetonitrile.

Crystals suitable for single-crystal XRD analysis could not be obtained for Cage^4+^ or its host-guest complexes after several attempts using a variety of solvents (MeCN and DMF) and antisolvents (Et_2_O, ^*i*^Pr_2_O, ^*t*^BuOMe, DCM, hexanes), so DFT structural optimizations were performed. The optimized structure of Cage^4+^ (Fig. 2A) has a coplanar arrangement of its porphyrin faces, with a centroid-to-centroid separation of 11.9 Å. A distance of 20.5 Å was found between the benzylic carbon atoms at opposite ends of the cage. The dimensions of the host were altered only slightly in the optimized structure of C_70_@Cage^4+^ (Fig. 2C); spacing between the porphyrin faces is expanded to 12.2 Å and the distance between the benzylic carbon atoms is contracted to 20.2 Å. The spacing of the porphyrin faces is expanded much more (to 13.4 Å) in the optimized structure of C_60_@Cage^4+^ (Fig. 2B), and the benzylic carbon atom spacing is contracted considerably (to 19.0 Å). Thus, the geometry of Cage^4+^ is well suited to hosting C_70_, while considerable distortion is needed to host C_60_. These results help to explain the experimentally observed preference for binding C_70_*vs.* C_60_, especially since Cage^4+^ consists primarily of sp^2^ to sp^2^ linkages that are not expected to provide much conformational flexibility.

The dimensions of Cage^4+^ and its fullerene complexes were evaluated experimentally using DOSY NMR spectroscopy (Fig. S25–S27†). A diffusion coefficient (*D*) of 8.76 × 10^−11^ m^2^ s^−1^ was determined for Cage^4+^ in dmso-*d*_6_, corresponding to an effective hydrodynamic radius^16^ (*r*) of 1.25 nm, which matches well with the van der Waals distance (*ca.* 2.5 nm) across the widest dimension of the computationally optimized structure (Fig. 2A). A similar diffusion constant was measured for C_60_@Cage^4+^ (*D* = 8.34 × 10^−11^ m^2^ s; *r* = 1.31 nm, Fig. S26†), while C_70_@Cage^4+^ diffuses more slowly (*D* = 7.01 × 10^−11^ m^2^ s; *r* = 1.56 nm, Fig. S27†) even though the optimized structures of the host-guest complexes suggest that the latter exhibits less structural distortion relative to the empty host. The disparity of the diffusion coefficient of C_70_@Cage^4+^ relative to the empty host may reflect altered interactions with other solutes, such as the PF_6_^−^ counteranions. The influence of anions on the diffusion of the nanocage was assessed by comparing the diffusion of Cage^4+^ as its PF_6_^−^, BF_4_^−^, and BPh_4_^−^ salts. The BF_4_^−^ counteranions increase the rate of diffusion of Cage^4+^ substantially (*D* = 1.14 × 10^−10^ m^2^ s^−1^, Fig. S29†), while BPh_4_^−^ anions result in Cage^4+^ diffusing only slightly faster (*D* = 9.46 × 10^−11^ m^2^ s^−1^, Fig. S28†) than observed for its PF_6_^−^ salt. Since counteranions influence the diffusion of Cage^4+^, we speculate that the different diffusion rates for the host-guest complexes arise from the C_60_*vs.* C_70_ guests having differing influences on the association of the cage with its PF_6_^−^ counteranions.

Travelling-wave ion-mobility spectrometry (TWIMS) was employed as an additional technique to compare the effective sizes—specifically the collisional cross sections—of Cage^4+^ and its complexes with fullerene guests.^17^ Conveniently, this gas-phase technique allows for selective measurement of the 4+ ions of the cage and host-guest complexes to eliminate any complicating influences of counteranions. Under optimized conditions, Cage^4+^ had a drift time of 23.99 ms, while the same experimental parameters provided slightly longer drift times of 25.62 ms and 26.01 ms for C_60_@Cage^4+^ and C_70_@Cage^4+^, respectively (Fig. S45†). Thus, the host-guest complexes have very similar effective cross sections, while the empty host appears slightly smaller, which is consistent with TWIMS measurements reported for other cationic hosts that encapsulate fullerenes.^17*a*^

### Electrochemical characterization

**Fig. 3:**
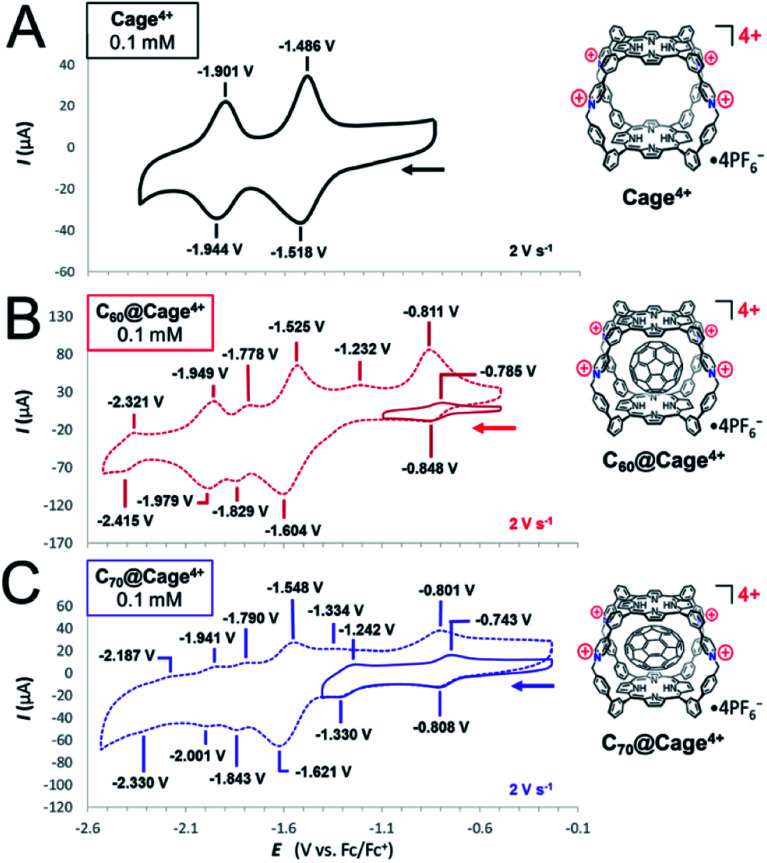
Cyclic voltammograms of (A) Cage^4+^, (B) C_60_@Cage^4+^, and (C) C_70_@Cage^4+^ recorded in DMF containing 0.1 M [Bu_4_N][PF_6_] supporting electrolyte. All samples contained a 0.1 mM concentration of the analyte and the data presented here was recorded at a scan rate of 2 V s^−1^.

The electron-accepting properties of fullerenes are important to many of their possible applications,^6^ so cyclic voltammetry was used to examine how encapsulation by Cage^4+^ affects the redox properties of C_60_ and C_70_ (Fig. 3 and Table 1). First, Cage^4+^ was examined in the absence of fullerenes, revealing reversible reductions at −1.51 V and −1.92 V *vs.* Fc^+/0^ in DMF (Fig. 3A). By comparison with monomeric porphyrins representing each half of Cage^4+^ (Fig. S46 and S47†), the first reduction wave of the cage was assigned to the reductions of both porphyrin faces and the four pyridinium groups, while the smaller, more negative reduction feature corresponds to the second reduction of each porphyrin. However, the measured currents are closer to a 2 : 1 ratio rather than the expected 3 : 1.**We also attempted to characterize Cage^4+^ and its host-guest complexes by differential pulse voltammetry, but reliable DPV measurements could not be obtained, possibly due to adhesion of Cage^4+^ to the electrode. Deviation from ideal behavior is unsurprising considering that anions affect the diffusion rate of Cage^4+^ (see above), and interactions between the cage and anions will be diminished as the cage is reduced. Assignments of the redox features of Cage^4+^ were confirmed by UV-vis-NIR monitoring of redox titrations (see below).

**Table 1:** Comparison of the reduction potentials^a^ of C_60_, C_70_, Cage^4+^, C_60_@Cage^4+^, and C_70_@Cage^4+^

	1^st^ C_*n*_ reduction	2^nd^ C_*n*_ reduction	1^st^ Cage reduction	3^rd^ C_*n*_ reduction	2^nd^ Cage reduction	4^th^ C_*n*_ reduction
C_60_^b^	−0.77 V	−1.23 V	—	−1.82 V	—	−2.36 V
C_70_	−0.77 V	−1.19 V	—	−1.71 V	—	−2.21 V
C_60_@Cage^4+^	−0.82 V	[–1.33 V]^c^	−1.56 V	−1.80 V	−1.96 V	−2.37 V
C_70_@Cage^4+^	−0.78 V	−1.29 V	−1.58 V	−1.82 V	−1.97 V	[–2.26 V]^d^
Cage^4+^	—	—	−1.50 V	—	−1.92 V	—

a Unless otherwise noted, potentials refer to *E*_1/2_ values relative to Fc^+/0^ for reversible redox couples measured in DMF containing 0.1 mM analyte and 0.1 M [Bu_4_N][PF_6_] supporting electrolyte.

b Reduction potentials of C_60_ are taken from ref. 18.

c *E*_pc_ value for a reduction of the C_60_ guest that overlaps with a reduction of the host.

d This redox couple is poorly defined.

The host-guest complexes C_60_@Cage^4+^ and C_70_@Cage^4+^ both show additional redox couples arising from the fullerene guests (Fig. 3B and C). The first fullerene-centred reductions are reversible for both complexes and occur at potentials (*E*_1/2_ = −0.82 V, C_60_@Cage^4+^; −0.78 V, C_70_@Cage^4+^) that are slightly negative of those of free C_60_ (−0.77 V)^18^ and C_70_ (−0.77 V)††††Reduction potentials of C_70_ were measured in DMF (0.1 M TBAPF6) following the technique described in ref. 18. in DMF (Table 1). Measurements in PhCN (Fig. S56–S59†) also showed that the first two reduction potentials of C_60_ and C_70_ are not altered much by encapsulation in Cage^4+^ (Table S1†). These observations are surprising since other cationic hosts shift the reduction potentials of fullerenes by as much as +500 mV.^4*c*^ We reasoned that anions associated with C_60_@Cage^4+^ and C_70_@Cage^4+^ might mitigate the electrostatic influence of the host, especially given the high electrolyte concentration used in CV measurements. However, treatment of C_60_@Cage^4+^ with 1 equiv. of a diaryl viologen radical cation as a mild chemical reductant (*E*_1/2_ = −0.71 V, Fig. S53†) resulted in nearly the same equilibrium ratio of C_60_@Cage^4+^ to C_60_^−^@Cage^4+^ in samples containing from 0.1 mM to 100 mM concentrations of PF_6_^−^ (Fig. S73†). Thus, the reduction potential of the guest does not appear to be affected by PF_6_^−^ concentration. Likewise, addition of up to 100 mM concentrations of TBAOTf or TBABPh_4_ did not substantially alter the reduction of the guest by the chemical reductant. Since neither the anions nor solvent appear essential for preserving the reduction potentials of the fullerenes upon encapsulation, we speculate that the electrostatic influence of Cage^4+^ is balanced well by the electron-donating influence of its porphyrin walls towards the fullerene guests.

Regardless of the specific cause, it is notable that Cage^4+^ minimally affects the first reduction potentials of C_60_ and C_70_. Likewise, it is noteworthy that, even at scan rates as high as 50 V s^−1^, the first reductions of C_60_@Cage^4+^ and C_70_@Cage^4+^ show nearly ideal reversibility (Δ*E*_pc_ ≤ 72 mV, Fig. S60†), whereas most hosts severely diminish the reversibility of fullerene redox processes,^4*a*,*d*,8*a*^ indicating slowed electron-transfer rates or low stability of the reduced complexes. Thus, Cage^4+^ is unique as a host that solubilizes fullerenes while preserving their reduction potentials and electron transfer capabilities. These features are likely to be useful since the inherent electron-accepting properties of fullerenes are well studied for a variety of applications,^19^ and other solubilization strategies (*e.g.*, covalent functionalization) also tend to alter the redox properties of fullerenes.^20^

Cage^4+^ and the fullerenes show greater mutual influence on each other's redox behaviour when more negative reductions of the host-guest complexes are examined, though even these processes are only modestly perturbed. The second reduction of C_70_@Cage^4+^ (*E*_1/2_ = −1.29 V) is observed clearly at a potential that is 100 mV negative of the free C_70_^−^/C_70_^2−^ redox couple (*E*_1/2_ = −1.19 V), while the second fullerene reduction of C_60_@Cage^4+^ overlaps partially with reduction of the host, also representing about a 100 mV cathodic shift relative to free C_60_^−^/C_60_^2−^ (Table 1). The first reductions of the host are also shifted cathodically by 60 to 80 mV for each complex (Table 1), suggesting the bound fullerides exert a small electrostatic influence on the host. However, the next cage-centered reductions are altered by a smaller amount (40 to 50 mV, Table 1). We speculate that electrostatic repulsion between the host and guests in their more reduced states (*i.e.*, when both are in anionic states) triggers expulsion of the fulleride anions, such that reduction processes corresponding to the empty host and free guest are observed at more negative potentials. Consistent with this possibility, the most negative observable reduction of C_60_@Cage^4+^ appears at nearly the same potentials as the free C_60_^3−^/C_60_^4−^ redox couple (note that the most negative reduction of C_70_@Cage^4+^ was not clear enough for comparisons).

### ^1^H NMR binding studies of C_60_^*n*−^ (*n* = 1, 2)

**Fig. 4:**
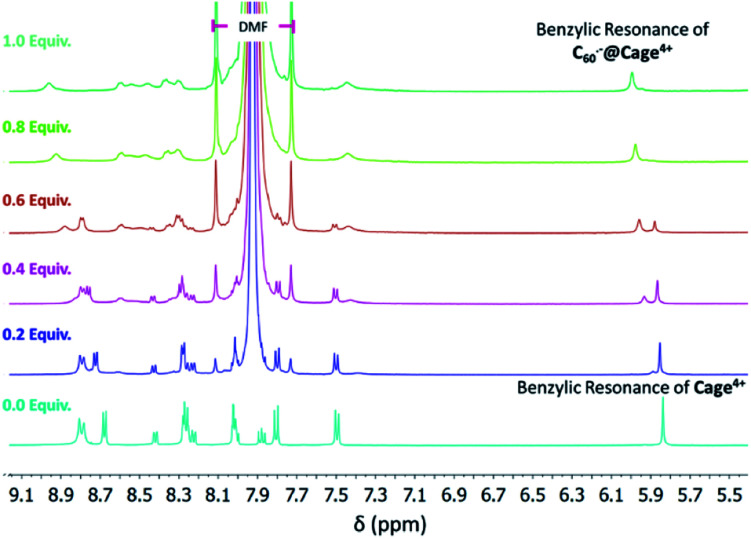
Changes to the ^1^H NMR spectra of a solution of Cage·4PF_6_ in CD_3_CN in response to titration with a solution of [Cp*_2_Co][C_60_] in DMF. Broad new signals are observed corresponding to the host-guest complex C_60_^−^@Cage^4+^ formed upon association of paramagnetic C_60_^−^ in Cage^4+^.

We were unable to acquire high quality CV data in MeCN, so we sought to directly observe the binding of fulleride anions by Cage^4+^ in CD_3_CN to complement the electrochemical measurements performed in DMF and PhCN. The association of C_60_^−^ and C_60_^2−^ in Cage^4+^ was evident *via* titration experiments monitored by ^1^H NMR spectroscopy. A solution of [Cp*_2_Co][C_60_] in DMF was titrated in 0.1 equiv. increments into a solution of Cage^4+^ in CD_3_CN, resulting in the appearance of a new benzylic CH signal at *ca.* 5.95 ppm for the host-guest complex C_60_^−^@Cage^4+^. Despite the radical character of the guest, this new benzylic CH signal was resolved clearly enough to be observed as distinct from that of the empty host, which decreased steadily as C_60_^−^ was added (Fig. 4 and S31†). Similar results were obtained upon titration of Cage^4+^ with a solution of C_60_^2−^ (Fig. S32†).

Integration of the benzylic CH resonances of empty Cage^4+^*vs.* those of C_60_^−^@Cage^4+^ and C_60_^2−^@Cage^4+^ provided association constants of approximately 10^7^ M^−1^ and 10^5^ M^−1^, respectively, which are lower than that (≥6.0 × 10^8^ M^−1^) estimated for C_60_@Cage^4+^ in MeCN. Each order of magnitude decrease in the strength of association should correspond to a −59 mV shift of the reduction potential of the guest, so the binding constants determined in these experiments imply greater changes to the redox properties of C_60_ than were measured electrochemically in DMF and PhCN. However, the ^1^H NMR measurements likely underestimate the association constants because broadening of the resonances of the host-guest complexes leads to systematic lowering of the integrals of these signals, making it difficult to accurately quantify the concentrations of C_60_^−^@Cage^4+^ and C_60_^2−^@Cage^4+^. Thus, while accurate quantitative comparisons cannot be made, the NMR studies confirm that C_60_^−^ and C_60_^2−^ associate strongly with Cage^4+^ in MeCN, which provides qualitative agreement with CV measurements performed in DMF and PhCN. The effects of further reduction could not be examined by NMR methods but were examined by UV-vis-NIR spectroscopy as described in the next section.

### UV-vis-NIR spectroscopy of Cage^4+^ and its host-guest complexes with fullerenes and fullerides

The UV-vis spectrum of Cage^4+^ in DMF displays one Soret band and one set of Q-peaks, suggesting the two distinct porphyrin faces of the cage are electronically similar, as is consistent with their overlapping redox features observed by cyclic voltammetry. Reduction of the cage with decamethylcobaltocene (Cp*_2_Co) causes the appearance of low-energy (>650 nm) absorbances that are characteristic of porphyrin radical anions (Fig. S62†).^21^ The intensity of these bands increases steadily with the addition of up to 4 equiv. Cp*_2_Co, and then less dramatically up to 6 equiv., which is consistent with nearly equal distribution of reduction over the two porphyrin faces and four pyridinium groups until all six sites are fully reduced. Addition of two more equivalents of Cp*_2_Co causes the low-energy bands to increase again, indicating further reduction of the porphyrins to their dianionic states (Fig. S62†). These results support the interpretation of the CV data described above for Cage^4+^, which is also consistent with DFT results indicating that the eight lowest unoccupied orbitals of the cage are centered on either the porphyrin faces or the pyridinium groups (Fig. S76†).

**Fig. 5:**
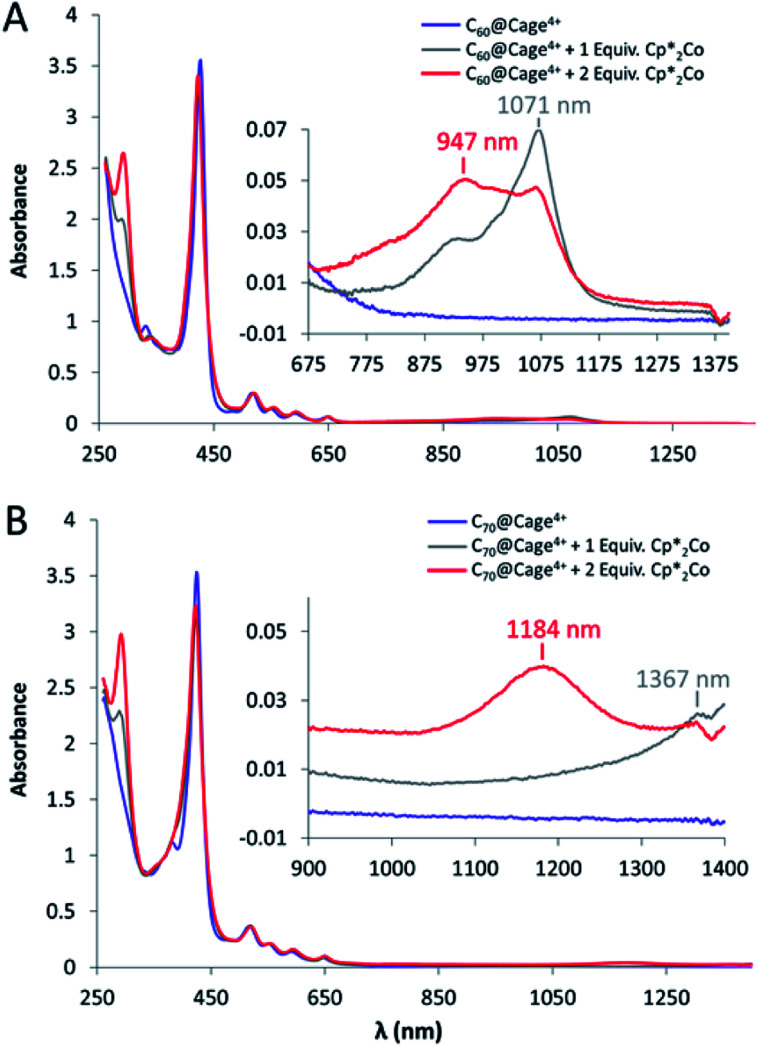
UV-vis-NIR spectra of (A) C_60_@Cage^4+^ and (B) C_70_@Cage^4+^ upon sequential 1 equiv. additions of Cp*_2_Co. Insets show magnified views of NIR absorbances that are characteristic of the anionic fullerenes. Spectra were recorded at 0.125 mM concentrations of the host-guest complexes in DMF in a 1 mm pathlength cuvette.

Since the first two fullerene reductions occur at least slightly positive of the first reductions of Cage^4+^, it was possible to spectroscopically observe host-guest complexes of the singly and double reduced fullerenes in the 4+ charged host. The complex C_60_@Cage^4+^ was reduced *via* two sequential 1 equiv. additions of Cp*_2_Co, resulting in the appearance of characteristic absorbances for the C_60_ mono- and di-anions between 800–1200 nm (Fig. 5A).^6^ Similarly, sequential 1 e^−^ reductions of C_70_@Cage^4+^ with Cp*_2_Co produces spectra that display absorbances consistent with those of C_70_^−^ and C_70_^2−^ (Fig. 5B).^22^ The Soret band and Q-peaks of Cage^4+^ remain unchanged in these spectra, confirming that the host is unreduced.

Further reduction of the host-guest complexes was examined to determine if ejection of the fulleride guests occurs as was suggested by CV data. The complex C_60_@Cage^4+^ was treated with 8 equiv. of Cp*_2_Co in MeCN, reducing the host by 6 e^−^ and the C_60_ guest by 2 e^−^. The reduced host has negligible solubility in MeCN, resulting in complete disappearance of any porphyrin spectral features, but a significant concentration of the C_60_^2−^ anion remained detectable by its NIR absorption band, representing 22% of the C_60_ that was initially present. This concentration represents a binding constant of 3.2 × 10^4^ M^−1^ for C_60_^2-^@Cage^2-^ if it is assumed that full equilibration between bound and unbound C_60_^2−^ occurs prior to precipitation of the reduced host. This result confirms that reduction of the host weakens the binding of C_60_^2−^ by at least a small amount. In contrast, performing this experiment using C_70_@Cage^4+^ did not yield the free C_70_^2−^ anion in solution. These observations are consistent with CV results showing that the C_70_^2−^/C_70_^3−^ redox couple differs by 100 mV for encapsulated *vs.* free C_70_ (Table 1), indicating the fulleride remains bound in the reduced host, at least on the short timescales of these experiments.

## Conclusion

In summary, a new covalently linked nanocage Cage^4+^ has been synthesized on a gram scale using simple synthetic methods and inexpensive starting materials. This cage binds the fullerenes C_60_ and C_70_ with high affinities (*K*_a_ > 10^8^ M^−1^ in MeCN) and displays excellent selectivity for extracting C_70_ from mixtures containing an excess of C_60_. Cage^4+^ also strongly binds the 1- and 2-states of the fullerenes. The different oxidation states of the host-guest complexes were characterized by several methods, including cyclic voltammetry and spectroscopic techniques, revealing that the electronic properties of the fullerenes are surprisingly unperturbed by encapsulation in Cage^4+^, in contrast to recent reports of fullerene guests in other cationic cages.^4*a*,*c*,*d*^

These findings suggest that Cage^4+^ may be a particularly useful host for separating and solubilizing fullerenes. Its performance is comparable to or better than that of many notable fullerene-binding hosts in terms of overall affinity and the ability to separate C_70_ from C_60_, while the low-cost scalable synthesis makes Cage^4+^ much more promising for applications. Additionally, since the encapsulation of fullerenes by this host does not appear to substantially alter their redox behaviour, C_60_@Cage^4+^ and C_70_@Cage^4+^ may be directly useful as solubilized fullerene derivatives that preserve this important functional property of the fullerene guests.

## Author contributions

D. A. Rothschild and M. C. Lipke designed Cage^4+^. D. A. Rothschild and W. P. Kopcha carried out syntheses and performed experiments. D. A. Rothschild and A. Tran carried out DFT calculations. All authors contributed to analyzing data and preparing the results for publication.

## Conflicts of interest

There are no conflicts to declare.

## Supplementary Material

SC-013-D2SC00445C-s001
