# Supplementary material for: Gram-scale synthesis of a covalent nanocage that preserves the redox properties of encapsulated fullerenes
Source: Chem Sci. 2022 Apr 13;13(18):5325–32. doi: 10.1039/d2sc00445c (PMC9093146; doi:10.1039/d2sc00445c)
Supplement: SC-013-D2SC00445C-s001 [file SC-013-D2SC00445C-s001.pdf]

Electronic Supplementary Material (ESI) for Chemical Science.
This journal is © The Royal Society of Chemistry 2022

Gram-Scale Synthesis of a Covalent Nanocage that Preserves the Redox Properties of Encapsulated Fullerenes

Daniel A. Rothschild, William P. Kopcha, Aaron Tran, Jianyuan Zhang, and Mark C. Lipke*

Department of Chemistry and Chemical Biology, Rutgers The State University of New Jersey
123 Bevier Road, Piscataway NJ 08854

Email: ml1353@chem.rutgers.edu

Supporting Information

Table of Contents:

1. General Considerations……………………………………………………………………S2
2. Synthetic Procedures……………………………………………………………………S3
3. NMR Characterization……………………………………………………………………S9
4. Mass Spectrometry……………………………………………………………………S29
5. Electrochemical Studies……………………………………………………………………S37
6. UV-Vis-NIR Spectroscopy……………………………………………………………………S45
7. Fluorescence Measurements of C_{60} and C_{70} Association in **Cage**$^{4+}$……………………S52
8. DFT Calculations……………………………………………………………………S54
9. References……………………………………………………………………S55

1. General Considerations

Sources: Unless otherwise specified, commercially available chemicals and solvents were used as received from (1) Fisher: acetonitrile, dichloromethane, *N*,*N*-dimethylformamide, methanol, and toluene; (2) Acros Organics: ammonium hexafluorophosphate, bis(triphenylphosphino)palladium(II) chloride, benzonitrile, 2-(2-methoxyethoxy)ethanol; (3) Alfa Aesar: ferrocene (Fc), iodomethane; (4) Cambridge Isotopes: dimethylsulfoxide-d_6 (D-99.9%), acetonitrile-d_3 (D-99.8%) and benzene-d_6 (D-99.5%); (5) Oakwood Chemicals: pyridine-4-boronic acid hydrate, 4-(hydroxymethyl)phenylboronic acid, 1,1′-bis(diphenylphosphino)ferrocene; ammonium tetrafluoroborate; sodium tetraphenylborate (6) Sigma Aldrich: decamethylcobaltocene (Cp^*_2Co), cobaltocene (Cp_2Co), phosphorus tribromide (7) Fischer: sodium sulfate; (8) Henan Fullerene Co. Ltd.: fullerene-C_{60}, fullerene-C_{70}. Fulleride salts $[Cp^*_2Co][C_{60}]$ and $[Cp^*_2Co]_2[C_{60}]$ were prepared using modified literature procedures[1] and the adapted procedures are described below. Tetrakis-(3-bromophenyl)porphyrin,[2] [1,1′-bis(diphenylphosphino)ferrocene]dichloro-palladium,[3] methyl viologen,[4] 4-methoxyphenyl viologen,[5] 4-phenylpyridine,[6] 4-phenylbenzyl bromide,[6] tetrabutylammonium triflate,[7] and tetrabutylammonium tetraphenylborate[8] were prepared according to previously reported methods.

Purification: Dry solvents (toluene, *N*,*N*-dimethylformamide, dichloromethane, acetonitrile) were obtained by sparging HPLC grade solvents with argon for 1 h followed by percolation through solvent drying columns supplied by Pure Process Technologies. Benzonitrile was dried over sodium sulfate and sparged for 30 minutes prior to use for synthesis. Benzonitrile for electrochemical studies was dried over CaH_2 for 72 h and then subjected to three freeze-pump-thaw cycles before being distilled under vacuum and stored under N_2. For air-sensitive NMR measurements, NMR solvents were dried over a suitable drying agent (CaH_2 for CD_3CN; NaK alloy for C_6D_6), subjected to three freeze-pump-thaw cycles, distilled by vacuum transfer, and then stored in an N_2 atmosphere glovebox prior to use. Ferrocene was sublimated under static vacuum prior to use. Decamethylcobaltocene (Cp^*_2Co) and cobaltocene (Cp_2Co) were sublimated under dynamic vacuum and stored at −25 °C in an N_2 atmosphere glovebox prior to use. Tetrabutylammonium hexafluorophosphate ($TBAPF_6$) was recrystallized three times from absolute ethanol, dried at 125 °C under dynamic vacuum for ≥ 12 hours, and stored in an N_2 atmosphere glovebox prior to use.

Air-Sensitive Procedures: Air-sensitive fulleride salts were prepared and handled in an N_2 atmosphere glovebox. Air-sensitive NMR and UV-vis-NIR spectra were acquired on samples that were sealed under N_2 in NMR tubes or cuvettes using PTFE stoppers.

Physical Measurements: NMR spectra were recorded at 25 °C using a Bruker AVANCE Neo 500 MHz spectrometer or a Varian VNMRS 500 MHz spectrometer. ^1H NMR spectra were referenced to the residual proteo signal of the solvent. A CH Instruments 600E potentiostat was used for all electrochemical experiments. UV-vis-NIR spectra were recorded using a Shimadzu UV-2600i spectrophotometer equipped with an ISR-2600Plus integrating sphere detector that provides a 220 – 1400 nm wavelength range for solution absorbance measurements. Electron spray ionization high resolution mass spectra (ESI-HRMS) were obtained via direct injection using a Waters Xevo G2-XS QToF mass spectrometer. A standard of Leu-Enkephalin (Waters, Milford, MA) was injected in tandem with the sample for in-process calibration.

2. Synthetic Procedures

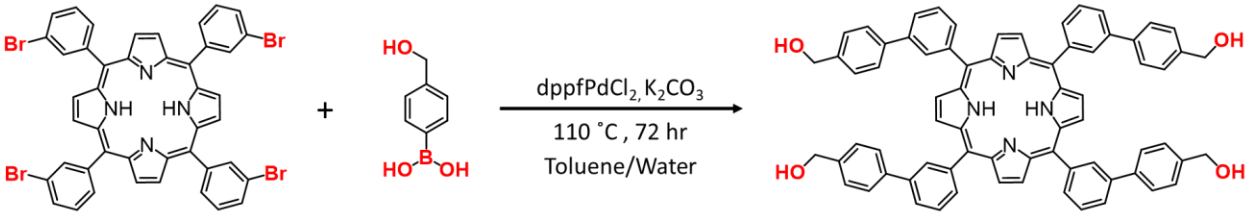

3 4

Synthesis of tetrakis-3-(4-(hydroxymethyl)phenyl)phenylporphyrin (4). A 500 mL Schlenk flask was charged with tetrakis(3-bromophenyl)porphyrin (5.00 g, 5.37 mmol), 4-(hydroxymethyl)-phenylboronic acid (16.18 g, 107.4 mmol), [1,1'-bis(diphenylphosphino)ferrocene]dichloro-palladium (0.786 g, 1.07 mmol), potassium carbonate (14.88 g, 107.4 mmol), and a PTFE-coated magnetic stir bar. The flask was then equipped with a reflux condenser before being cycled three times between vacuum and N_2 to establish an inert atmosphere. A cannula was then used to add a mixture of 150 mL of toluene and 70 mL of water that had been sparged with N_2 for 20 min. The reaction mixture was then stirred and heated to 110 °C for 72 h before being allowed to cool to room temperature. Toluene/water was removed via rotary evaporation. The resulting purple residue was suspended in deionized H_2O, sonicated, filtered, and washed with additional deionized H_2O (3 x 100 mL). Thin-layer chromatography (silica, 95:5 DCM:MeOH) at this stage shows several spots including 4 purple bands, the slowest moving of which corresponds to the desired product. The crude product was dissolved in DMF, deposited on silica by rotary evaporation, and then purified by chromatography over silica gel, eluting with a gradient of 10 to 20 % MeOH in DCM. (Note: porphyrin **4** is slightly soluble in DCM or MeOH, but a 20/80 DCM/MeOH provides good solubility. On smaller scales, this DCM/MeOH mixture can be used instead of DMF to deposit the product on silica). The desired product, which began eluting at 15 % MeOH in DCM, was the fourth porphyrin band to be separated and was eluted fully using 20 % MeOH in DCM. Fractions containing the desired product were dried by rotary evaporation. The resulting purple solid was suspended in 300 mL of water, collected by vacuum filtration, and then washed with cold DCM (2 x 20 mL) and cold methanol (2 x 20 mL) before drying under vacuum to provide **4** as a red-purple powder (3.374 g, 60.0%). ^{1}H NMR (500 MHz, DMSO-d_6) δ 8.96 (s, 8 H), 8.51 (br, 4 H), 8.23 (br, 4 H), 8.16 (d, J = 8.12 Hz, 4 H , 7.96-7.86 (m, 12 H), 7.45 (m, 8 H), 5.21 (m, 4 H), 4.55 (m, 8 H), -2.82 (s, 2 H). ^{13}C{^{1}H} NMR (125 MHz, CD_3CN): δ 142.17, 141.85, 138.63, 138.05, 133.29, 132.45, 127.62, 127.12, 126.85, 126.25, 119.98, 62.57. ESI(+)-MS, M = $C_{72}H_{54}N_4O_4$; [**MH**]$^+$ Calculated: 1039.4223 m/z, Found: 1039.4408 m/z; [**MH$_2$**]$^{2+}$ Calculated: 520.21507 m/z, Found: 520.2151.

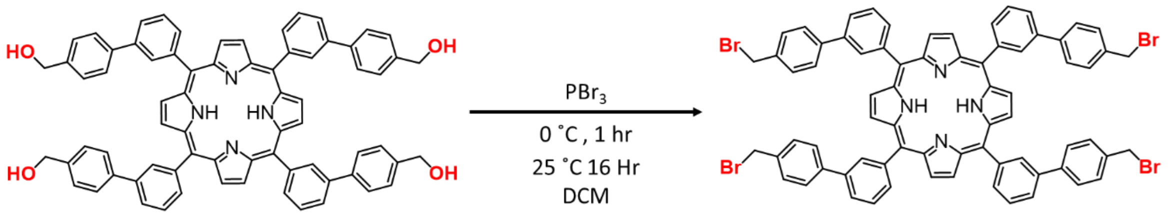

Synthesis of tetrakis-3-(4-bromomethylphenyl)phenylporphyrin (2). A 100 mL Schlenk flask was charged with porphyrin **4** (3.3 g, 3.18 mmol) and a PTFE stir bar before being sealed with a rubber septum and cycled three times between vacuum and N_2. Dry, air-free DCM (150 mL) was added via cannula and the purple suspension was cooled to 0 °C in an ice bath. Phosphorus tribromide (25.8 g, 9.1 mL, 95.0 mmol) was added dropwise to the stirred mixture to produce a deep green solution. The reaction mixture was allowed to warm slowly to room temperature and then stirred for an additional 12 h. With cooling provided by an ice/water bath, excess PBr_3 was quenched by dropwise addition of ice-cold water, followed by slow addition of a cold saturated sodium bicarbonate solution until a pH of 7 was reached (note: raising the pH above 7 results in lower yields of the desired product due to attack of hydroxides on the benzyl bromide positions). The water and dichloromethane layers were separated using a separatory funnel, and the aqueous layer was washed repeatedly with additional dichloromethane until the organic layer remained clear. The organic layers were combined and dried over magnesium sulfate. After filtration, the dichloromethane was removed via rotary evaporation. The residue was suspended in water, collected by vacuum filtration, and dried under vacuum to obtain a purple powder (3.06 g, 74.8 %). ^{1}H NMR (500 MHz, $CDCl_3$) δ 8.93 (s, 8H), 8.48 (s, 4H), 8.24 (d, J=7.16 Hz, 4H), 8.03 (d,t, J_d=7.85, J_t=1.35, 4H), 7.87-7.79 (m, 12H), 7.51 (d, 8.06, 8H), -2.71 (s, 2H). ^{13}C{^{1}H} NMR (125 MHz, DMSO-d_6): δ 141.83, 139.51, 137.96, 137.41, 135.56, 132.50, 129.91, 127.57, 127.29, 127.57, 127.29, 126.28, 119.86, 34.11.

*Mass spectroscopy could not be obtained for Porphyrin **2**, presumably because the benzyl bromide positions are unstable at elevated temperatures in the presence of water, hydroxide, or other nucleophiles which may be present in the mass spectrometer.

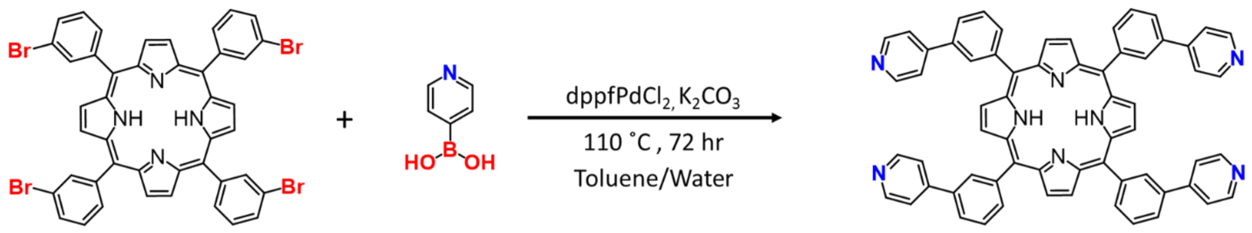

Synthesis of Tetrakis-3-(4-pyridyl)phenylporphyrin (1). A 500 mL Schlenk flask was charged with tetrakis(3-bromophenyl)porphyrin (6.00 g, 6.45 mmol), 4-pyridylboronic acid (15.85 g, 129 mmol), [1,1'-bis(diphenylphosphino)ferrocene]palladium(II) chloride (0.566 g, 0.774 mmol), potassium carbonate (18.30 g, 132 mmol), and a PTFE stir bar before being fitted with a reflux condenser and cycling 3x between vacuum and N_2. In a separate flask, a mixture of toluene/water (200 mL:100 mL) was sparged with N_2 for 20 min before being transferred to the reactants via cannula. The resulting suspension was then heated to 110 °C with stirring. Heating was ceased after 72 h and the toluene/water mixture was removed via

rotary evaporation, followed by suspending the red-brown residue in water, filtering, and washing with deionized H_2O (3 x 100 mL). At this stage, TLC (silica, 95:5 DCM:MeOH) shows several spots including four purple bands, the slowest of which corresponds the desired product. The purple solid was then dissolved in DCM, combined with silica gel, and evaporated to dryness. Column chromatography was then used to isolate the desired porphyrin by first eluting with 5% MeOH in DCM to flush out most impurities, followed by 8-10% MeOH to isolate the desired product, which is the last purple fraction eluted. Fractions containing the desired product were identified by TLC and rotovapped to dryness. The solid residue was suspended in water, filtered, and washed with water and cold methanol to obtain a dark purple powder (4.98 g, 83.7 %). ^{1}H NMR (500 MHz, $CDCl_3$) δ 8.92 (s, 8H), 8.71 (s, 8H), 8.53 (s, 4H), 8.33 (d, J=7.16 Hz, 4H), 8.10 (d, J=7.47 Hz, 4H), 7.91 (t, J=7.70 Hz, 4H), 7.75 (s, 8H). ^{13}C{^{1}H} NMR (125 MHz, DMSO-d_6: δ 150.29, 146.55, 142.07, 135.78, 134.95, 132.40, 127.89, 126.52, 121.61, 119.62. ESI(+)-MS, M = $C_{64}H_{42}N_8$: [**MH**]$^{1+}$ Calculated: 923.3611 m/z, Found: 923.3815 m/z; [**MH$_2$**]$^{2+}$ Calculated: 462.1844 m/z, Found: 462.1833 m/z; [**MH$_3$**]$^{3+}$ Calculated: 308.4589 m/z, Found: 308.4510; [**MH$_4$**]$^{4+}$ Calculated: 231.5961 m/z, Found: 231.5820 m/z.

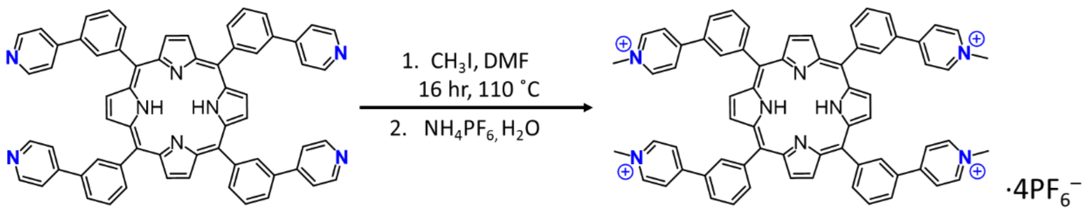

Synthesis of tetrakis-3-(*N*-methyl-4-pyridinium)phenylporphyrin. In a Schlenk flask, tetrakis-3-(4-pyridyl)phenyl)porphyrin (0.100 g, 0.108 mmol) was dissolved in 10 mL of dry DMF under a nitrogen atmosphere. Methyl iodide (0.113 g, 0.05 mL, 0.800 mmol) was added and the reaction mixture was heated under reflux for 16 h. After cooling, the solvent was evaporated under vacuum and the resulting residue was dissolved in water and precipitated out by addition of a large excess (0.5 g, 3.07 mmol) of ammonium hexafluorophosphate. The resulting precipitate was collected by filtration, washed with water, and dried under air to obtain a purple-brown powder (0.151 g, 89.2%). ^{1}H NMR (500 MHz, CD_3CN) δ 8.98 (s, 8H), 8.80 (s, 4), 8.66 (br, 8H), 8.54-8.36 (m, 16H), 8.09 (b, 4H), 4.31 (s, 12H), -2.81 (s, 2H). ^{13}C{^{1}H} NMR (125 MHz, DMSO-d_6): δ 154.19, 145.74, 142.53, 137.28, 132.55, 128.57, 128.02, 124.71, 119.27, 47.19. ESI(+)-MS, **M** = $C_{68}H_{54}N_8(PF_6)_4$: [**M-2PF$_6$**]$^{2+}$ Calculated: 636.1877 m/z, Found: 636.1922 m/z; [**M-3PF$_6$**]$^{3+}$ Calculated: 375.8038 m/z, Found: 375.7990; [**M-4PF$_6$**]$^{4+}$ Calculated: 245.6118 m/z, Found: 245.6011 m/z.

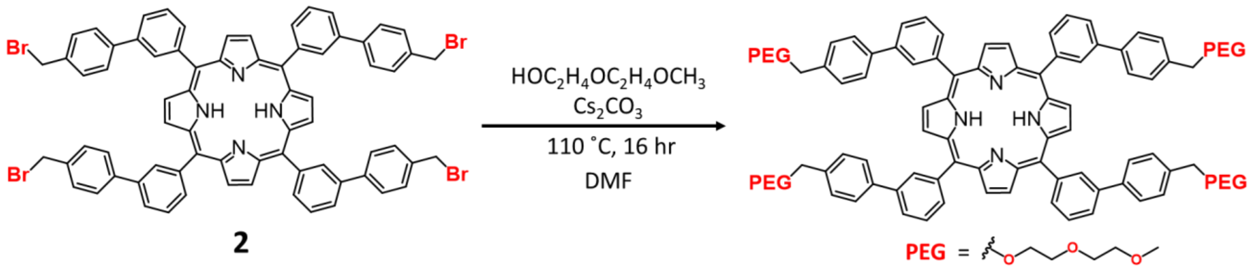

Synthesis of tetrakis-3-(4-(2-(2-methoxyethoxy)ethoxymethyl)phenyl)phenylporphyrin. In a 25 mL Schlenk flask, tetrakis-(3-(4-bromomethyl)phenyl)phenyl)porphyrin (0.050 g, 0.039 mmol) and cesium carbonate (1.0 g, 3.3 mmol) were dissolved in 10 mL of dry DMF under a nitrogen atmosphere. 2-(2-methoxyethoxy)ethanol (1.0 mL, 8.3 mmol) was added and the reaction mixture was heated to 55 °C for 16 h. The solvent was evaporated under vacuum, and the resulting residue was suspended in water, collected by vacuum filtration, washed with deionized water (2 x 100 mL) and dried under air to obtain a purple powder (0.049 g, 87 %). ^{1}H NMR (500 MHz, DMSO-d_6) δ 8.95 (s, 8H), 8.52 (br, 4H), 8.24(br, 4H), 8.16 (d, J= 8.00 Hz, 4H), 7.91 (br, 8H), 7.44 (br, 8H), 4.53 (br, 8H), 3.56 (m, 8H), 3.41 (m, 8H), 3.21 (m,12H), -2.83 (s, 2H). ^{13}C{^{1}H} NMR (125 MHz, (DMSO-d_6): δ 141.81, 138.62, 138.38, 137.96, 133.31, 132.45, 128.07, 126.86, 119.92, 71.54, 71.81, 69.57, 69.05, 57.97. ESI(+)-MS, **M** = $C_{92}H_{94}N_4O_{12}$: [**M**+H^{+}]$^{1+}$ Calculated: 1448.6946 m/z, Found: 1448.8075 m/z; [**M**+2H^{+}]$^{2+}$ Calculated: 375.8038 m/z, Found: 375.7990.

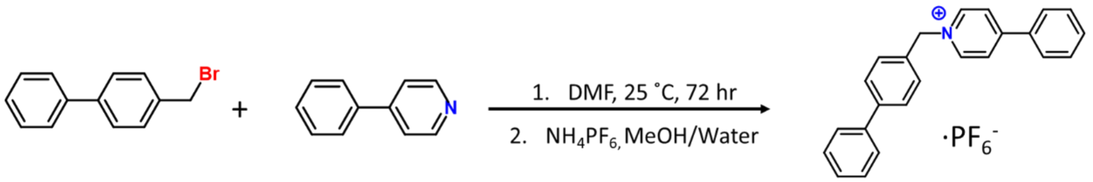

Synthesis of [*N*-(4-phenylbenzyl)-4-phenylpyridinium]PF$_6$. In a 50 mL round bottom flask, 4-phenylpyridine (244 mg, 1.57 mmol) and 4-phenylbenzyl bromide (776 mg, 3.14 mmol) were combined in 20 mL DMF and stirred at room temperature for 72 hours. The reaction was monitored by TLC until no more 4-phenylpyridine remained in solution. Solvent was removed under vacuum and the solid was further purified via hot recrystallization from 3 mL of DMF and 2 mL of toluene. The suspension was filtered and rinsed with diethyl ether (3 x 10 mL) and hexanes (3 x 10 mL). A while solid, [*N*-(4-phenylbenzyl)-4-phenylpyridinium]Br was obtained in an 82% yield (519 mg, 1.29 mmol). A salt exchange was performed on [*N*-(4-phenylbenzyl)-4-phenylpyridinium]Br to exchange the bromide for a hexafluorophosphate ion. In a 20 mL scintillation vial, 80 mg (198.8 µmol) was dissolved in MeOH (5 mL) and deionized water was added (2 mL) to decrease the solubility of the final product. The water/methanol solution was filtered though a 0.2 micron PFTE syringe filter to remove undissolved impurities. Then 100 mg of NH_4PF_6 was dissolved in 5 mL of deionized water and added to the scintillation vial to precipitate out [*N*-(4-phenylbenzyl)-4-phenylpyridinium]PF$_6$ as a white powder. The suspension was filtered and dried under vacuum to obtain 88 mg of product (94.6%).%). ^{1}H NMR (500 MHz, CD_3CN) δ 8.77 (d, J=6.46 Hz, 2H), 8.28 (d, J=6.52 Hz, 8H), 7.93 (d, J=7.63 Hz, zz8H), 7.76 (d, J=7.66 Hz, 2H), 7.67-7.64 (m, 5H), 7.55 (d, J=8.10 Hz, 2H), 7.49 (t, J=7.89 Hz, 2H), 7.41 (t, J=7.03 Hz, 1H), 5.74 (s, 2H), ^{13}C NMR (125 MHz, (DMSO-d_6): δ 155.15, 144.70, 141.07,139.14, 133.46, 133.41, 132.13, 129.60, 129.37, 128.96, 128.92, 128.14, 127.81, 127.40, 126.70, 124.92, 61.16. ESI(+)-MS: **M** = $C_{24}H_{20}N(PF_6)$; [**M** – PF_6^{-}]$^{1+}$ Calculated: 322.1596 m/z, Found: 322.1560 m/z.

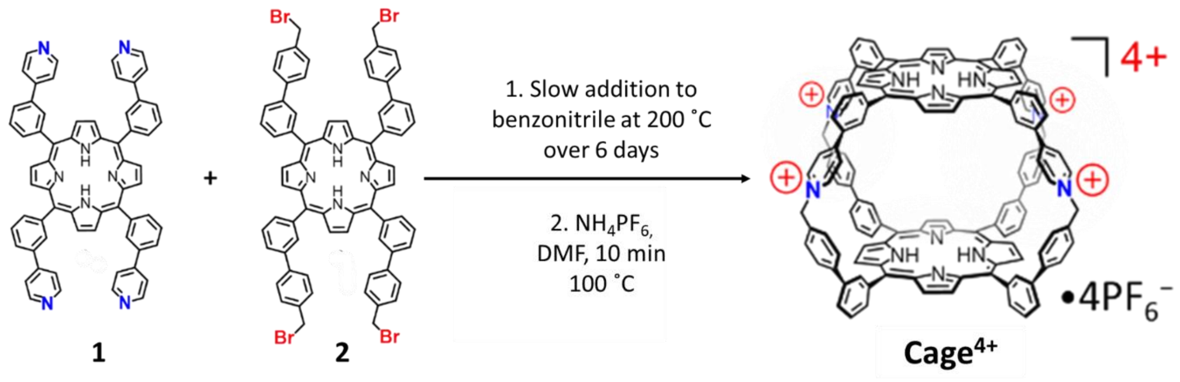

Synthesis of Cage•4PF$_6$. In a 1 L two-neck round-bottom flask, 500 mL of dry benzonitrile (PhCN) was sparged with N_2 for 20 min. A reflux condenser was then attached to one neck of the flask, and the other neck was fitted with a rubber septum that was secured with copper wire. The PhCN was heated under reflux (ca. 200 °C external temperature) with stirring. Syringe pumps were then used to slowly and simultaneously add 20 mL benzonitrile solutions of **2** (300 mg, 0.232 mmol) and **1** (214.6 mg, 0.232 mmol) over 5 days (0.16 mL/h for each solution). Heating was continued for one more day after completing the addition. The mixture was then allowed to cool to room temperature, and a dark solid was collected by vacuum filtration and washed with dichloromethane, acetone, water, and DMF until the washings were clear. The remaining solid was heated to 100 °C for 10 min in DMF containing excess ammonium hexafluorophosphate. Undissolved solids were removed by filtration and washed with DMF to extract additional product until the filtrate was clear. The filtrates were combined and solvent was removed by rotary evaporation to produce a green residue. The green solid was suspended in water, collected on a filter, and washed with hot water until changing color to purple. The resulting purple powder was dried under air to yield 330 mg of pure **Cage•**4PF$_6$ (57.4%). ^{1}H NMR (500 MHz, CD_3CN) δ 8.81 (s, 8H), 8.78 (s, 8H), 8.69 (d, J=7.10 Hz,8H), 8.42 (d, J=7.33 Hz, 4H), 8.28-8.26 (m, 16H), 8.25 (d, J=7.40 Hz, 4H), 8.06-8.01 (m, 12H), 7.90 (t, J=7.83 Hz, 4H), 7.83 (d, J=8.5 Hz, 8H), 7.52 (d, J=8.5 Hz, 8H), 5.86 (s, 8H), -3.22 (s, 2H), -3.24 (s, 2H). ^{1}H NMR (500 MHz, DMSO-d$_6$) δ 9.15 (d, J= 6.75 Hz, 8H), 8.82 (s, 8H), 8.77 (s, 8H), 8.52 (d, J=6.75 Hz, 8H), 8.48 (d, J=7.71 Hz, 4H), 8.40 (d, J=7.61 Hz, 4H), 8.35 (s, 4H), 8.24 (d, J=7.31 Hz, 4H), 8.12-8.03 (m, 8H), 7.95-7.88 (m, 8H), 7.80 (d, J= 8.07 Hz, 8H), 7.57 (d, J=8.07), 6.01 (s, 8H), -3.31 (s, 2H), - 3.32 (s, 2H). ^{13}C{^{1}H} NMR (125 MHz, CD_3CN): δ 144.98, 143.55, 143.00, 139.68, 137.95, 137.75, 134.42, 134.30, 134.12, 132.71, 130.41, 129.59, 129.13, 128.58, 128.31, 127.61, 127.08, 120.47, 119.72, 64.09. ^{13}C{^{1}H} NMR (125 MHz, DMSO-d$_6$): δ 156.05, 144.60, 142.15, 141.63, 138.48, 136.98, 133.74, 133.61, 133.51, 133.18, 129.17, 128.43, 128.13, 127.81, 126.88, 126.34, 119.55, 119.02, 62.40. ESI(+)-MS, **M** = $C_{136}H_{92}N_{12}(PF_6)_4$: [**M** –2PF$_6^-$]$^{2+}$ Calculated: 1091.8426 m/z, Found: 1091.8270 m/z; [**M** – 3PF$_6^-$]$^{3+}$ Calculated: 679.5736 m/z, Found: 679.5664 m/z; [**M** – 4PF$_6^-$]$^{4+}$ Calculated: 473.4392 m/z, Found: 473.4401 m/z.

Gram-scale procedure: The above procedure was modified for larger scale synthesis of **Cage•**4PF$_6$ by using of a 2 L flask, twice the volume of PhCN (1 L), and faster addition (0.8 mL/h) of the porphyrin components dissolved in two separate 40 mL portions of PhCN. 1.75 g of **2** (1.36 mmol) and 1.25 g of **1** (1.36 mmol) were used to obtain 1.35 g of **Cage•**4PF$_6$ (40.3 %).

General Procedure for Anion Exchange: In a 20 mL scintillation vial, **Cage•**4PF$_6$ (10 mg, 4.04 µmol) was dissolved in a minimal amount of acetonitrile (5 mL). 10 mL of a saturated aqueous solution of the sodium salt of the desired anion (NH_4BF_4 or $NaBPh_4$) was added until a red-brown precipitate formed. The solid was centrifuged, filtered, and washed with deionized water (2 x 125 mL) to remove excess salt (88% for the BF_4^- salt, 90% for the BPh_4^- salt).

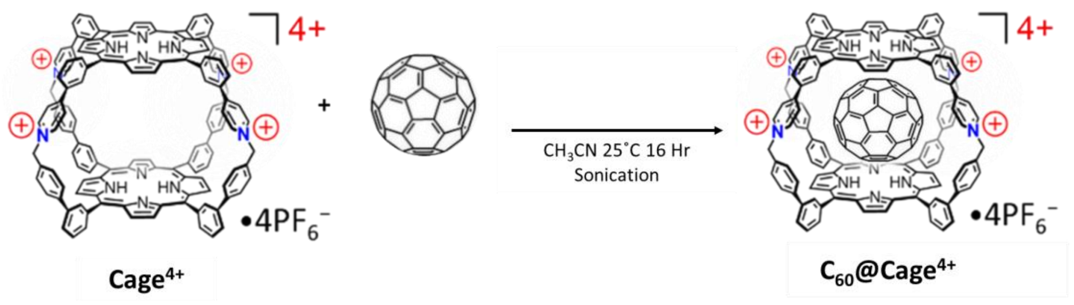

Preparation of [C_{60}@Cage]•4PF$_6$. In a 4 mL scintillation vial, **Cage**•4PF$_6$ (11 mg, 4.5 µmol) was dissolved in 2 mL of MeCN and C_{60} fullerene (10 mg, 14 µmol) was added to the purple solution before sonicating for 16 h. The resulting dark-brown solution was filtered through a 0.2 micron PTFE filter and the solvent was removed under vacuum to yield a brown solid (14 mg, 99 %). ^{1}H NMR (500 MHz, CD$_3$CN) δ 8.79 (s, 8H), 8.73 (s, 8H), 8.66 (d, J=7.33 Hz, 8H), 8.63 (d, 7.39 Hz, 4H), 8.36 (d, J=7.37, 4H), 8.28 (d, J= 8.00 Hz, 4H),8.18 (d, J=7.28, 8H), 8.08 (t, J=7.77 Hz, 4H),8.05 (d, J=8.08 Hz, 4H), 7.95 (br, 4H), 7.90 (t, J= 7.74 Hz, 4H), 7.86 (br, 4H), 7.34 (d, J= 8.46, 8H), 5.83 (s, 8H), -3.10 (s, 2H), -3.13 (s, 2H). ^{1}H NMR (500 MHz, DMSO-d$_6$) δ 9.08 (d, J=6.78 Hz, 8H), 8.84 (s, 8H), 8.70-8.65 (m, 12H), 8.43 (d, J=8.08 Hz, 4H), 8.39 (d, J=6.94 Hz, 8H), 8.34 (d, J=7.43 Hz, 4H) 8.16 (t, J= 7.43 Hz, 4H), 8.10 (d, J=8.4 Hz, 4H), 7.93 (t, J=7.43 Hz, 4H) 7.90 (s, 4H), 7.75 (s, 4H), 7.65 (d, J=8.40Hz, 8H), 7.26 (d,J=8.40 Hz, 8H), 6.01 (s, 8H), -3.19 (m, 4H). ^{13}C{^{1}H} NMR (125 MHz, CD$_3$CN): δ 158.39, 145.49, 144.13, 143.44, 142.92, 140.15, 140.40, 139.19, 134.20, 133.80, 133.71, 132.90, 129.91, 129.23, 126.86, 120.86, 120.07, 64.04. ^{13}C{^{1}H} NMR (125 MHz, DMSO-d$_6$): δ 156.50, 145.66, 143.15, 142.95, 142.44, 141.02, 139.35, 138.19, 137.18, 133.91, 133.76, 133.48, 133.13, 132.78, 129.26, 128.82, 128.48, 128.35, 128.23, 12.03, 126.14, 120.03, 119.44, 62.55. ESI(+)-MS, M = $C_{196}H_{92}N_{12}(PF_6)_4$: [**M** – 2PF$_6^-$]$^{2+}$ Calculated: 1452.3426 m/z, Found: 1452.3020 m/z; [**M** – 3PF$_6^-$]$^{3+}$ Calculated: 919.9070 m/z, Found: 919.8859 m/z; [**M** – 4PF$_6^-$]$^{4+}$ Calculated: 653.6770 m/z, Found: 653.6892 m/z.

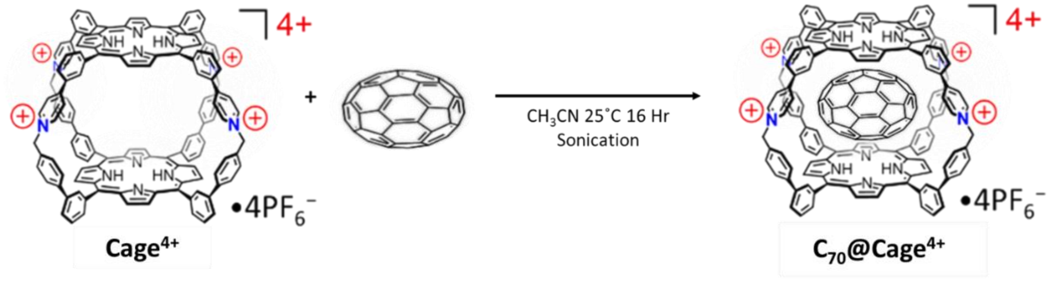

Preparation of [C_{70}@Cage]•4PF$_6$: In a 4 mL scintillation vial, **Cage**•4PF$_6$ (11 mg, 4.5 µmol) was dissolved in 2 mL of MeCN, and C_{70} fullerene (11 mg, 13 µmol) was added to the purple solution before sonicating for 16 h. The resulting dark-brown solution was filtered through a 0.2 micro PTFE filter and the solvent removed under vacuum to yield a brown solid (14.7 mg, 99.7%). ^{1}H NMR (500 MHz, CD$_3$CN) δ 8.76 (d, J= 7.06 Hz, 8H), 8.71 (s, 8H),8.66 (s, 8H), 8.61 (d, J=7.25, 4H), 8.35 (d, J=7.02 Hz, 4H), 8.31-8.25 (m, 12H) 8.13 (m, 4H), 8.10- 8.02 (m, 12H), 7.90 (t, J= 8.37 Hz, 4H), 7.81 (d, J=8.76, 8H), 7.44 (d, J= 8.76, 8H), 5.88 (s, 8H), -3.55 (s, 2H), -3.58 (s, 2H). ^{1}H NMR (500 MHz, DMSO-d$_6$) δ 9.18 (d, J= 5.22 Hz, 8H), 8.75 (s, 8H), 8.67 (d,

J=7.05 Hz, 8H), 8.60 (s, 8H), 8.50-8.40 (m, 12H), 8.31 (d, J=6.48 Hz, 4H), 8.15 (t, J= 6.77 Hz, 4H), 8.13-8.05 (m, 8H), 7.95 (s, 4H), 7.91 (t, J=7.48 Hz, 4H), 7.75 (d, J=7.48 Hz, 8H), 7.40 (d, J=7.48 Hz, 8H), 6.06 (s, 8H), -3.65 (m, 4H). $^{13}C\{^1H\}$ NMR (125 MHz, CD_3CN): δ 145.32, 143.93, 143.23, 142.82, 140.26, 138.98, 137.54, 134.02, 133.98, 133.64, 133.51, 129.71, 129.41, 129.03, 128.82, 128.43, 127.50, 126.69, 120.68, 119.87, 63.81. $^{13}C\{^1H\}$ NMR (125 MHz, DMSO-d_6): δ 155.81, 148.23, 145.22, 144.35, 144.17, 142.36, 141.86, 140.80, 140.48, 137.65, 136.59, 133.34, 133.14, 133.07, 132.87, 132.66, 128.86, 128.37, 128.24, 128.03, 127.90, 126.52, 125.83, 125.80, 119.44, 118.87, 62.23. ESI(+)-MS, **M** = $C_{206}H_{92}N_{12}(PF_6)_4$: [**M**– $2PF_6^-$]$^{2+}$ Calculated: 1512.3426 m/z, Found: 1512. 2781 m/z; [**M** – $3PF_6^-$]$^{3+}$ Calculated: 959.9070 m/z, Found: 959.8658 m/z; [**M**– $4PF_6^-$]$^{4+}$ Calculated: 683.6892 m/z, Found: 683.6636 m/z.

3. NMR Characterization

Instrumentation: Nuclear magnetic resonance (NMR) spectra were measured using a Bruker AVANCE Neo spectrometer or a Varian VNMR spectrometer, both with a 500 MHz working frequency for ^1H nuclei. ^1H NMR spectra were referenced to the residual proteo signal of the solvent (^1H δ 1.94 ppm for CD_2HCN in CD_3CN; ^1H δ 2.50 ppm for $(CD_3)(CD_2H)SO$ in DMSO-d_6; ^1H δ 7.26 ppm for $CHCl_3$ in $CDCl_3$). $^{13}C\{^1H\}$ NMR in DMSO-d_6 spectra were acquired with 256 scans and a 2 s relaxation delay, $^{13}C\{^1H\}$ NMR in CD_3CN were acquired with 10000 scans and a 4 s relaxation delay.

3a. ^1H and $^{13}C\{^1H\}$ NMR Characterization

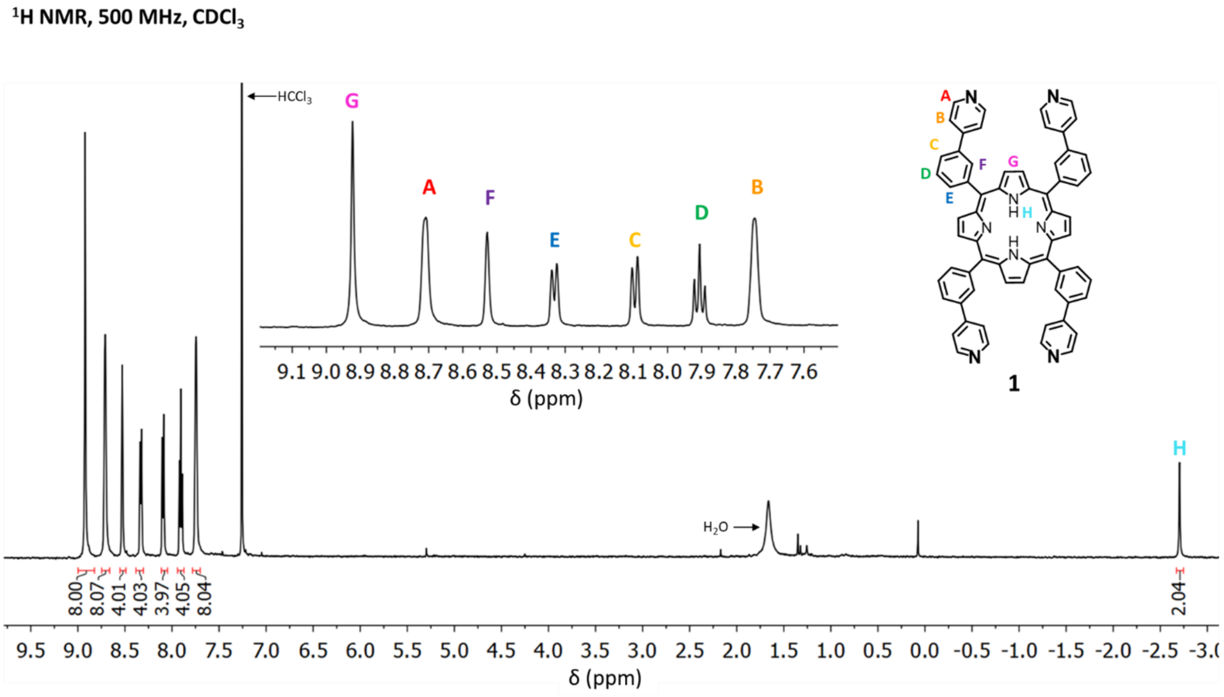

Figure S1. ^1H NMR spectrum of **1** (298K, $CDCl_3$, 500 MHz).

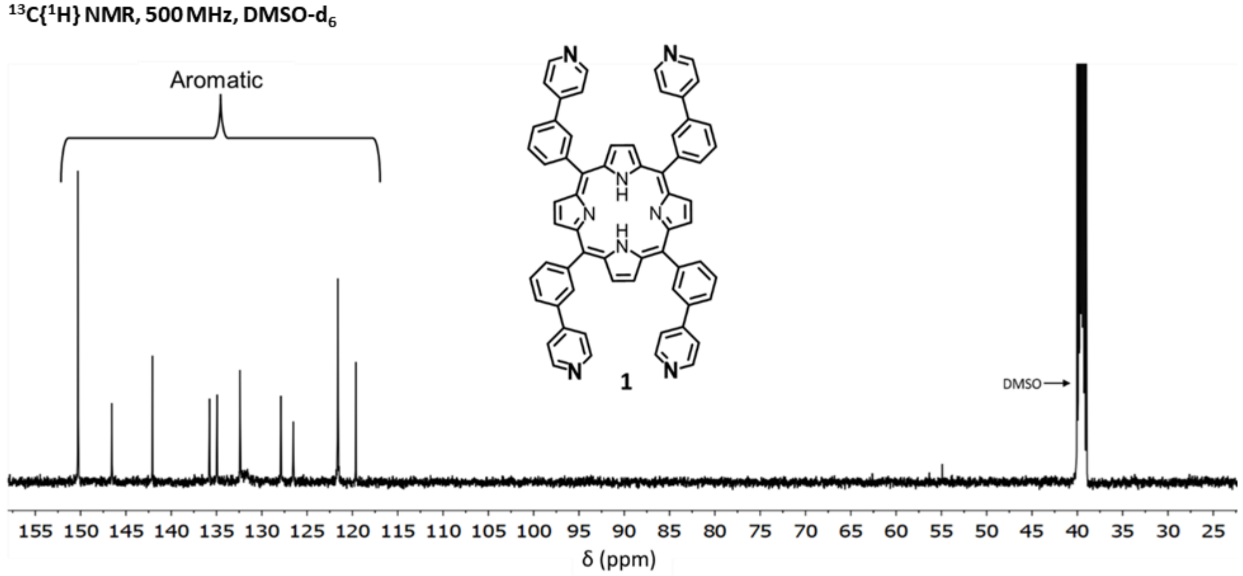

Figure S2. $^{13}C\{^{1}H\}$ NMR spectrum of **1** in DMSO-d_6 . Note that resonances for the quaternary carbons on the porphyrin macrocycle were not observed.

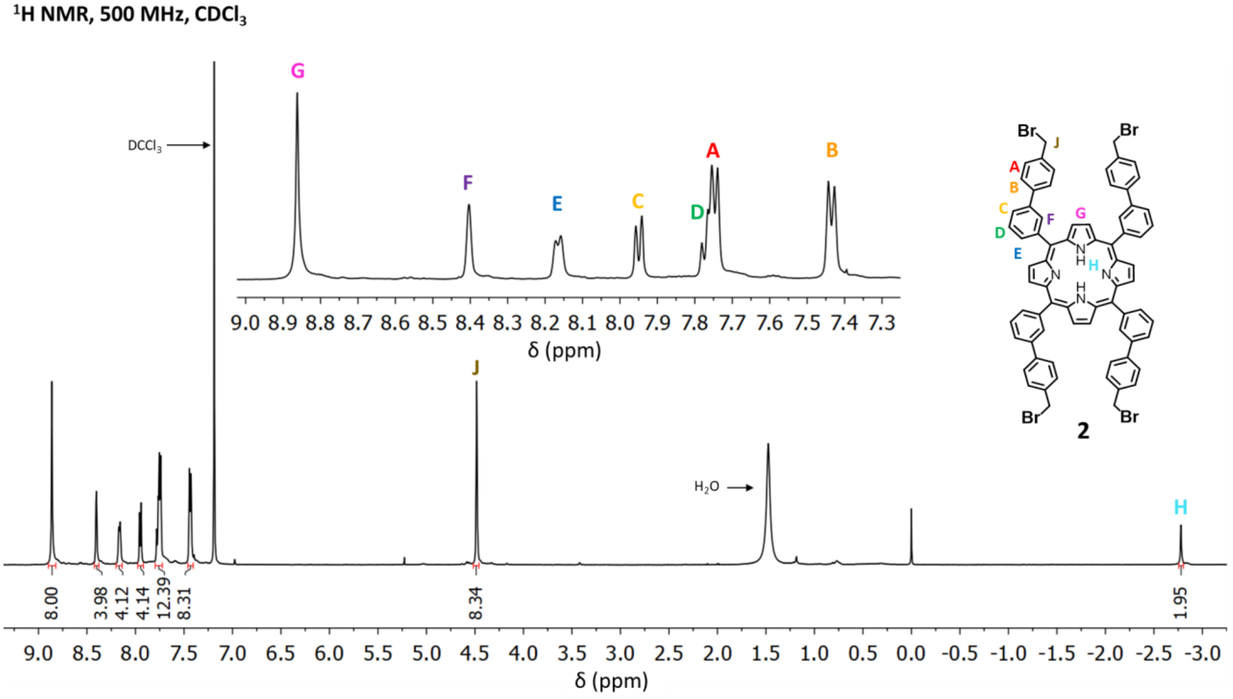

Figure S3. ^{1}H NMR spectrum of **2** (298K, $CDCl_3$, 500 MHz).

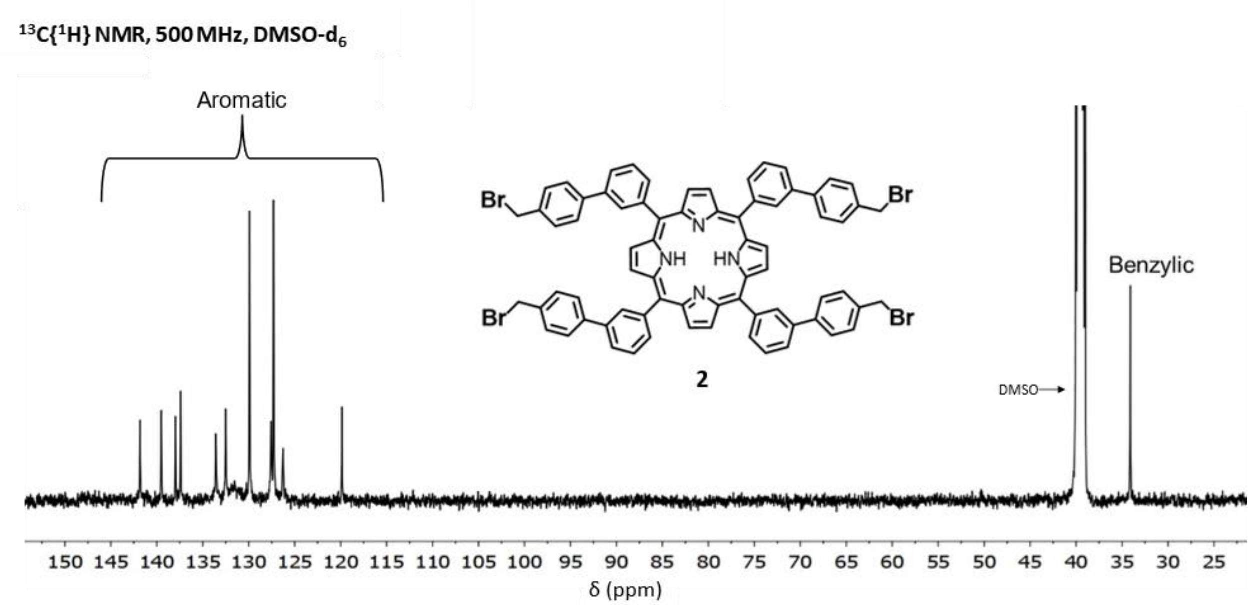

Figure S4. $^{13}C\{^1H\}$ NMR spectrum of **2** in DMSO-d$_6$. Note that some resonances for quaternary carbons on the porphyrin macrocycle were not observed.

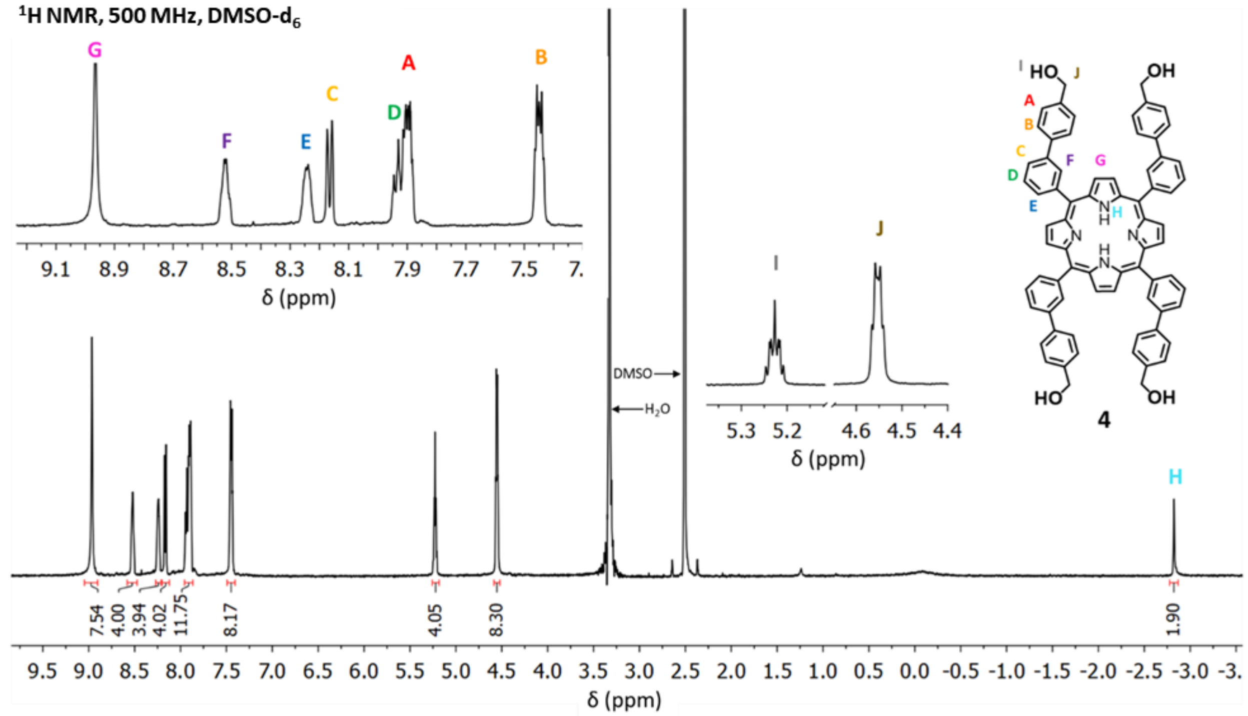

Figure S5. ^1H NMR spectrum of **4** (298K, DMSO-d$_6$, 500 MHz).

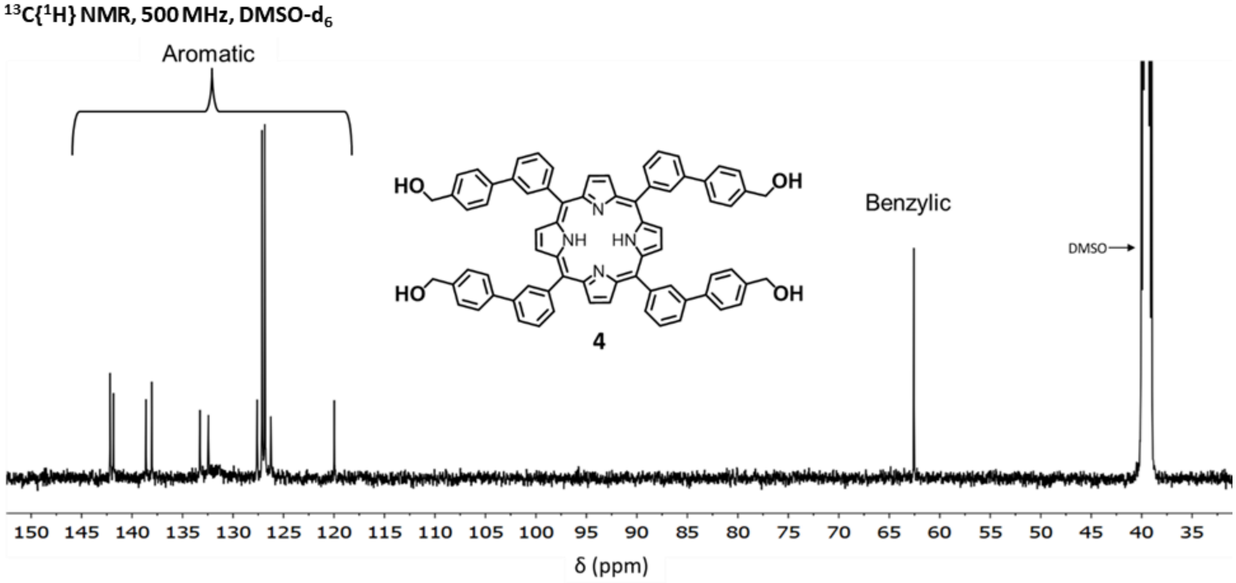

Figure S6. $^{13}C\{^1H\}$ NMR spectrum of **4** in DMSO-d$_6$. Note that some resonances for quaternary carbons on the porphyrin macrocycle were not observed.

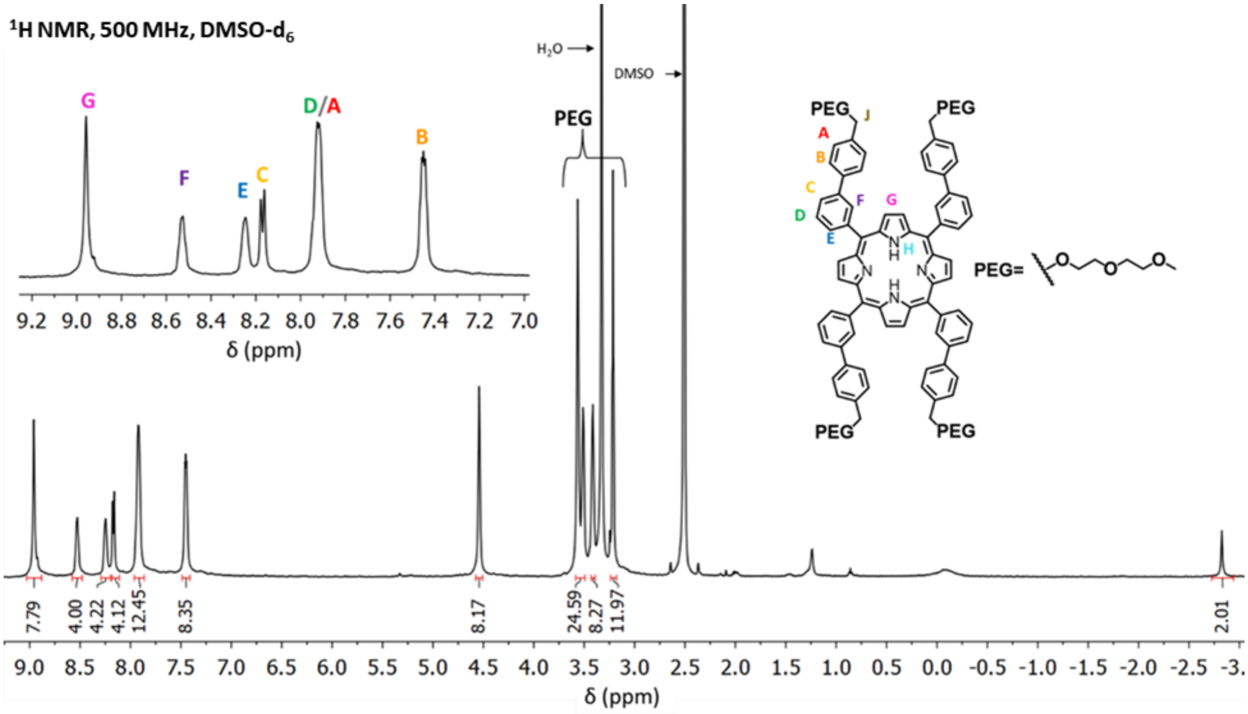

Figure S7. ^1H NMR spectrum of tetrakis-3-(4-(2-(2-methoxyethoxy)ethoxymethyl)phenyl)phenyl-porphyrin (298K, DMSO-d$_6$, 500 MHz).

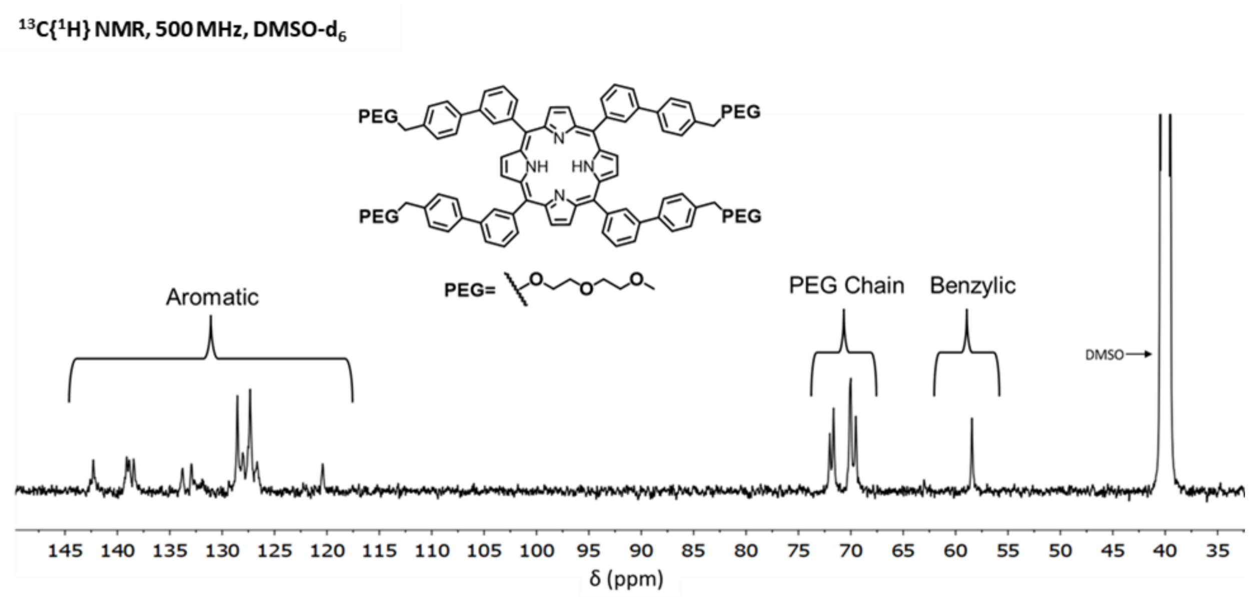

Figure S8. $^{13}C\{^{1}H\}$ NMR spectrum of tetrakis-3-(4-(2-(2-methoxyethoxy)ethoxymethyl)phenyl)phenyl-porphyrin in DMSO-d6 . Note that resonances for the quaternary carbons of the porphyrin macrocycle were not observed.

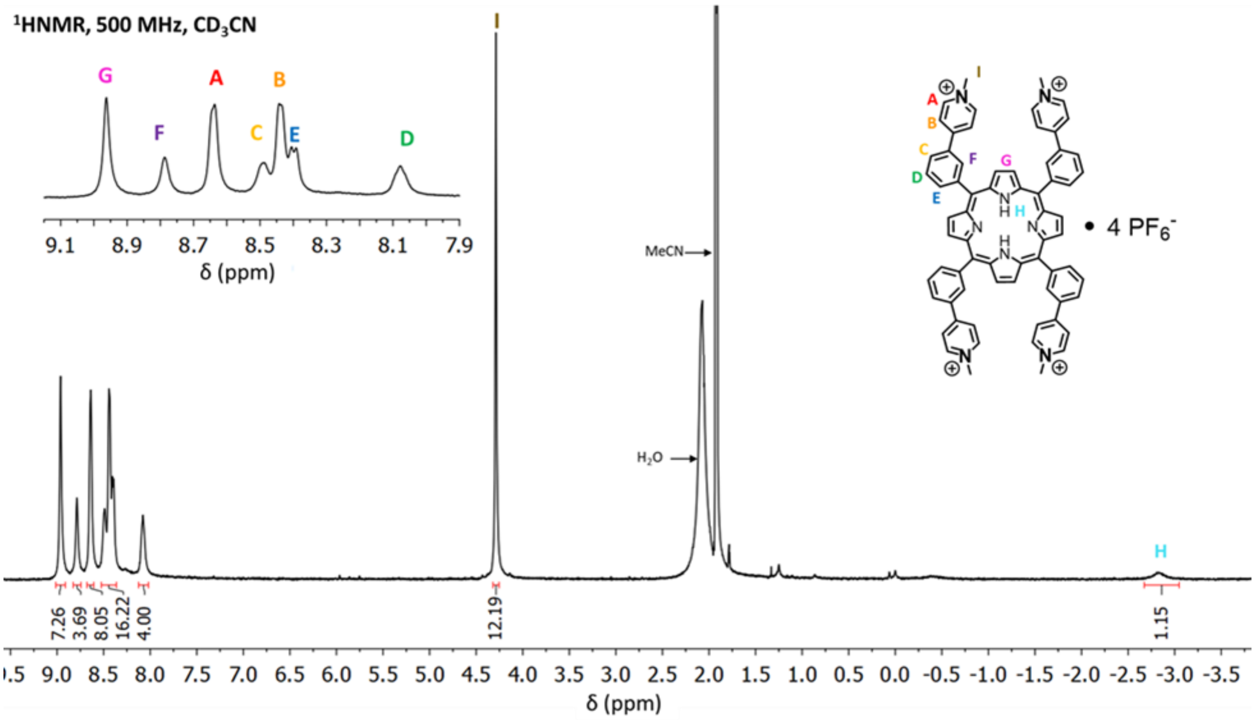

Figure S9. ^{1}H NMR spectrum of [tetrakis-3-(*N*-methyl-4-pyridinium)phenylporphyrin]•4PF6 (298K, CD_3CN, 500 MHz).

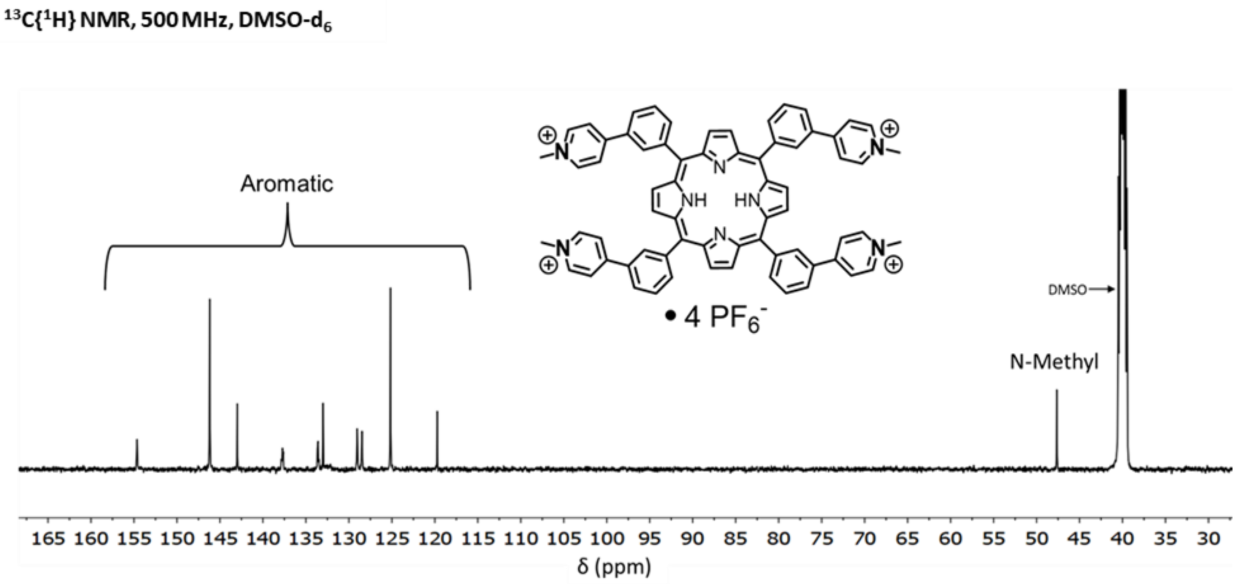

Figure S10. $^{13}C\{^1H\}$ NMR spectrum of [tetrakis-3-(*N*-methyl-4-pyridinium)phenylporphyrin]•4PF_6 in DMSO-d_6. Note that resonances for the quaternary carbons of the porphyrin macrocycle were not observed.

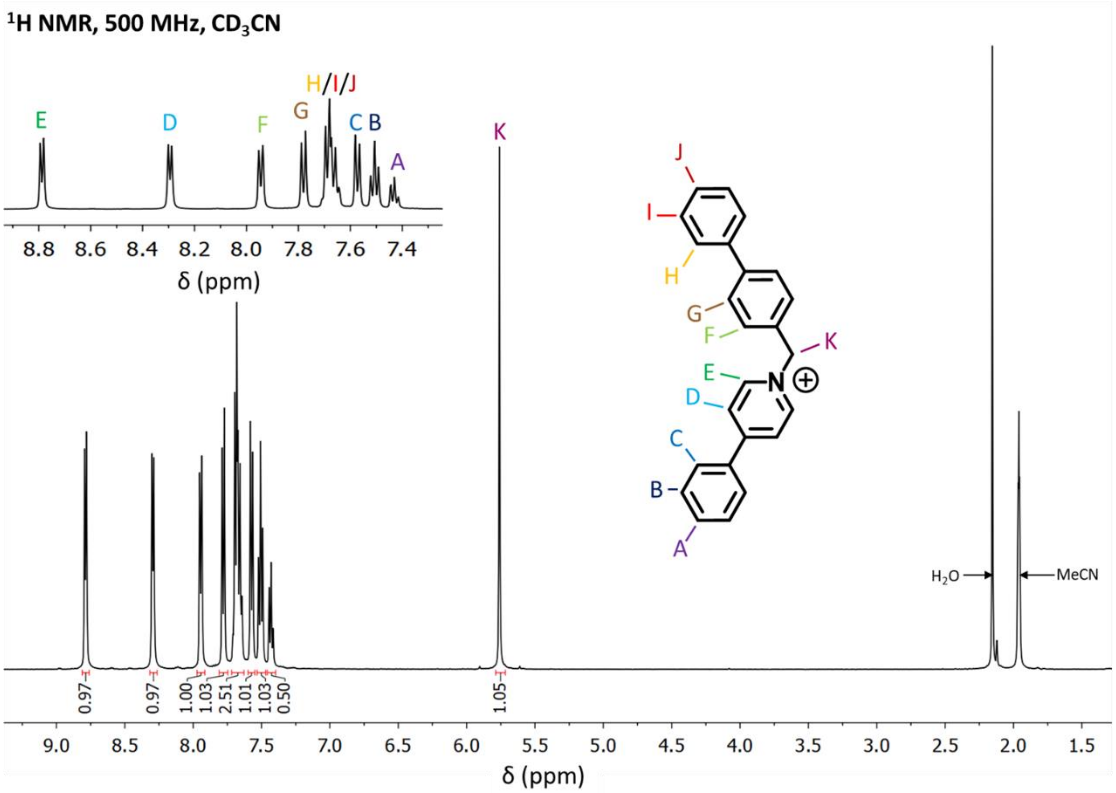

Figure S11. ^1H NMR spectrum of [*N*-(4-phenylbenzyl)-4-phenylpyridinium]PF_6 (298K, DMSO-d_6, 500 MHz).

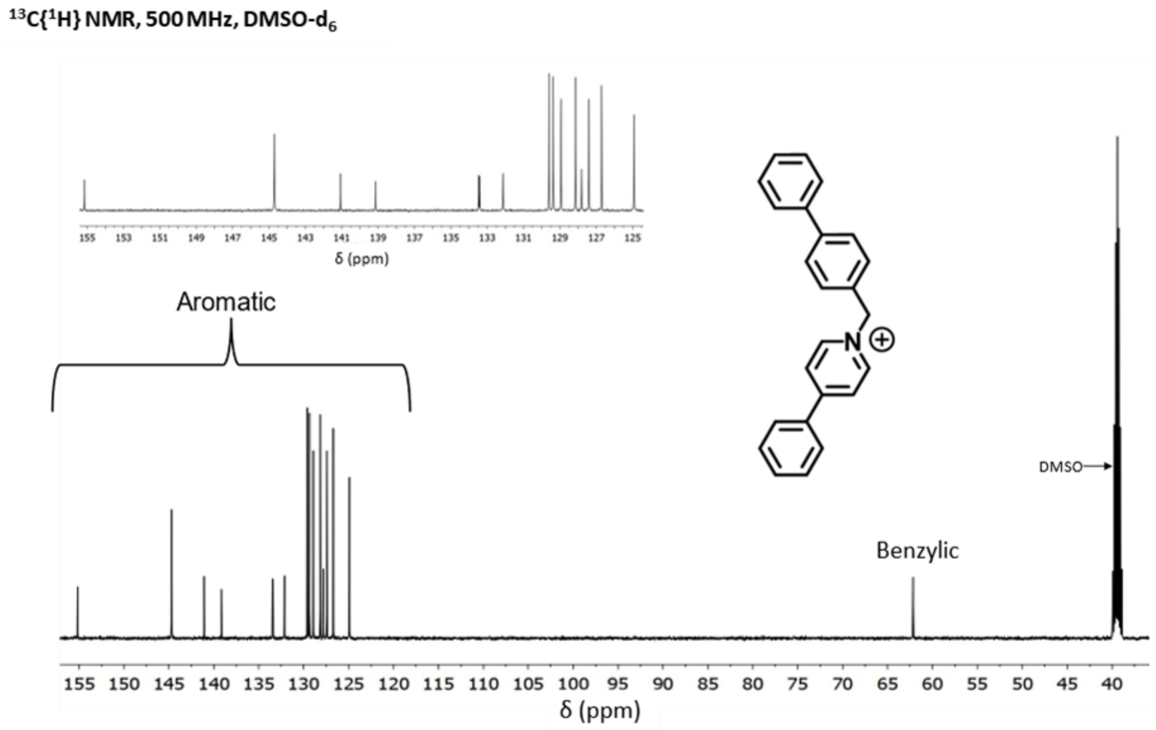

Figure S12. $^{13}C\{^{1}H\}$ NMR spectrum of [*N*-(4-phenylbenzyl)-4-phenylpyridinium]PF_6 in DMSO-d_6.

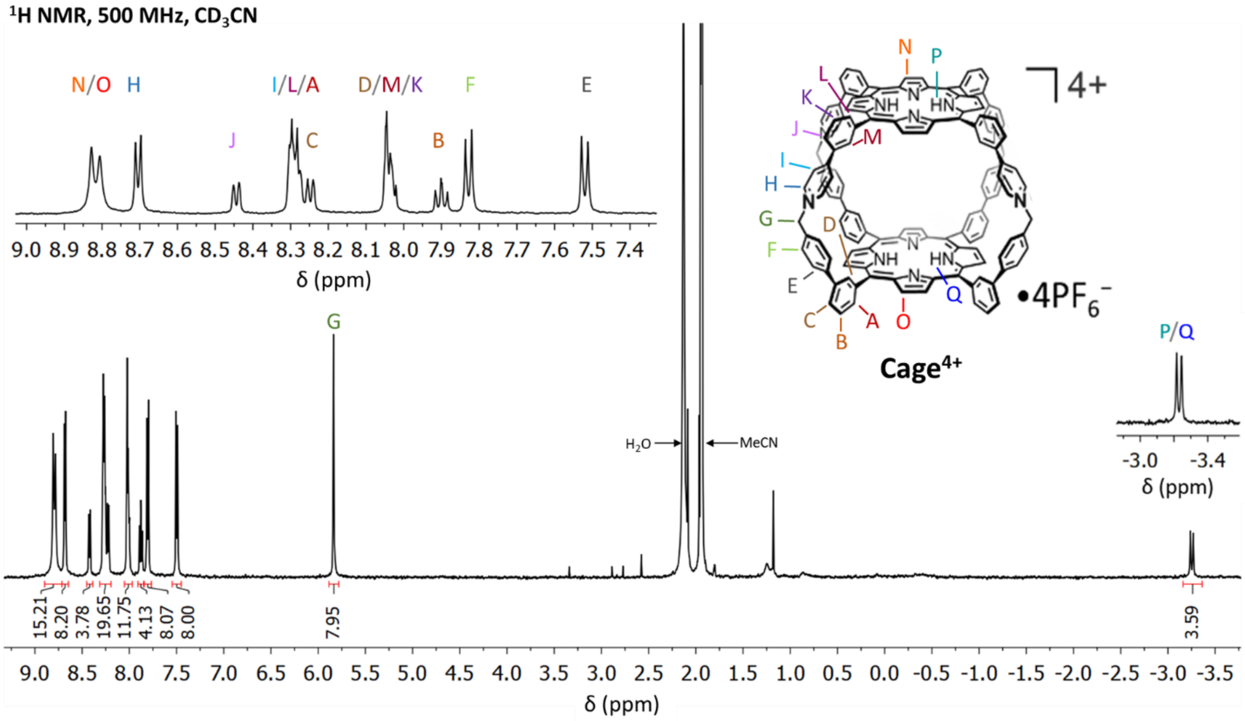

Figure S13. ^{1}H NMR spectrum of **Cage**•$4PF_6$ (298K, CD_3CN, 500 MHz).

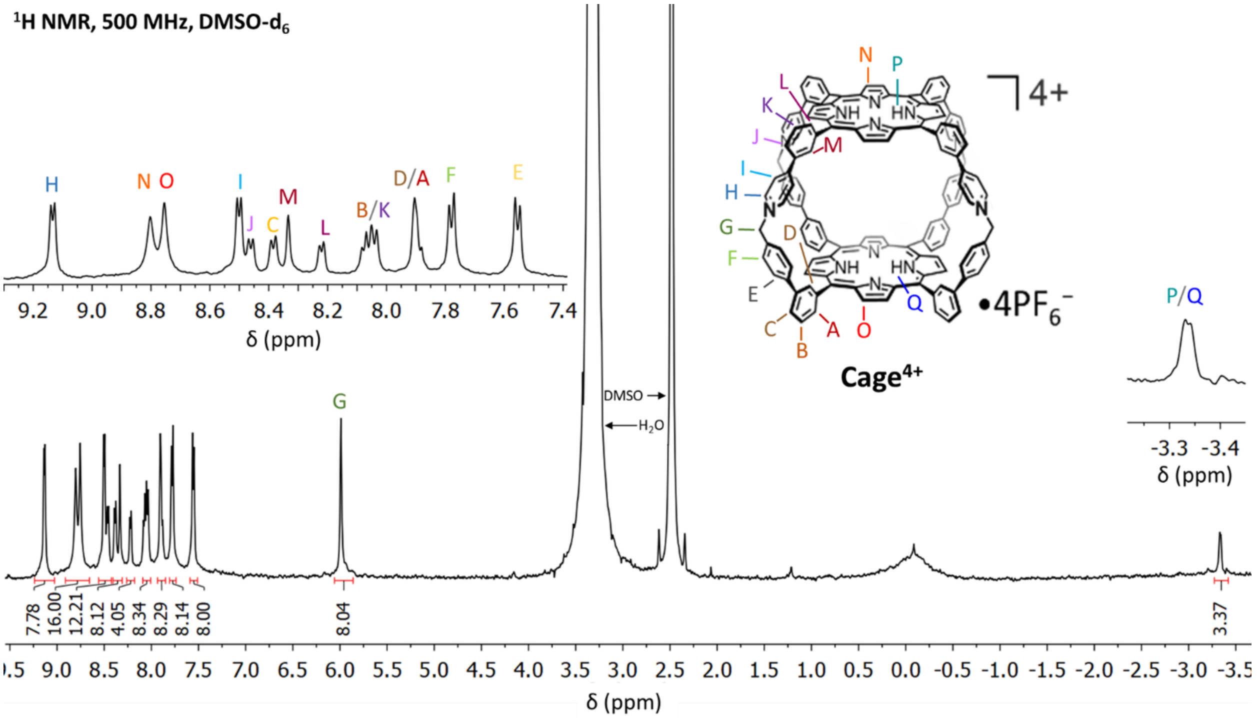

Figure S14. ^{1}H NMR spectrum of **Cage**•4PF$_6$ (298 K, DMSO-d$_6$, 500 MHz).

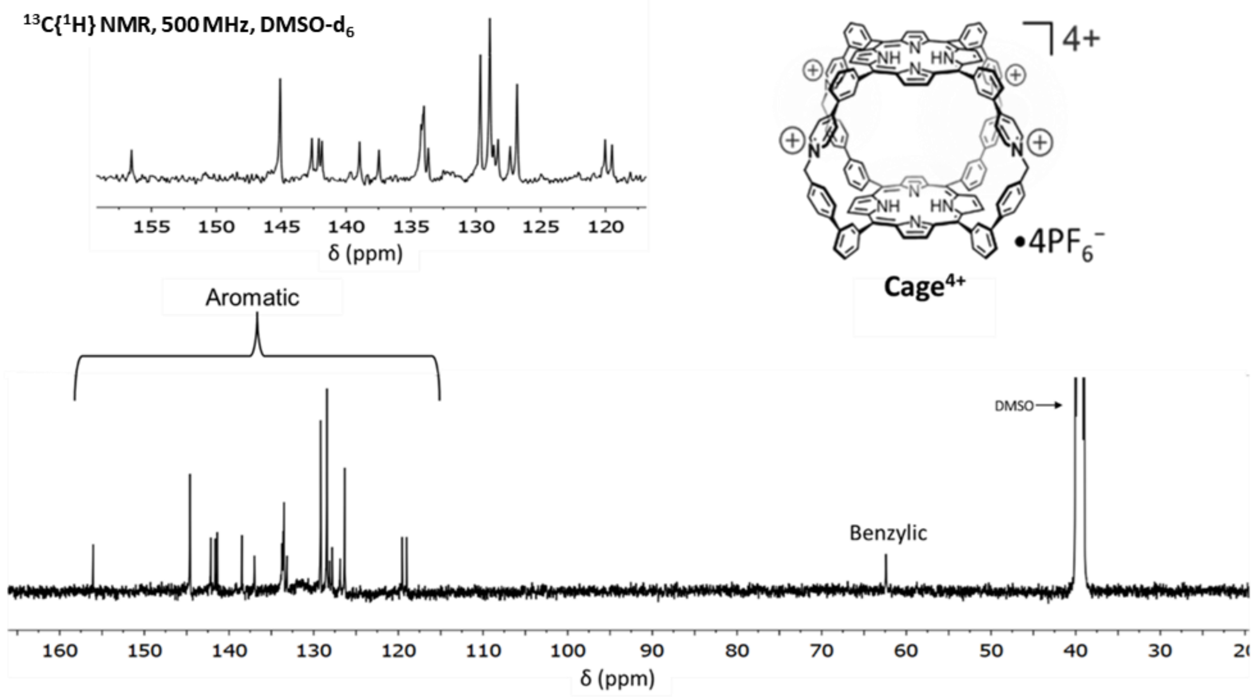

Figure S15. ^{13}C{^{1}H} NMR spectrum of **Cage**•4PF$_6$ in DMSO-d$_6$. Note that some resonances for quaternary carbons were not observed.

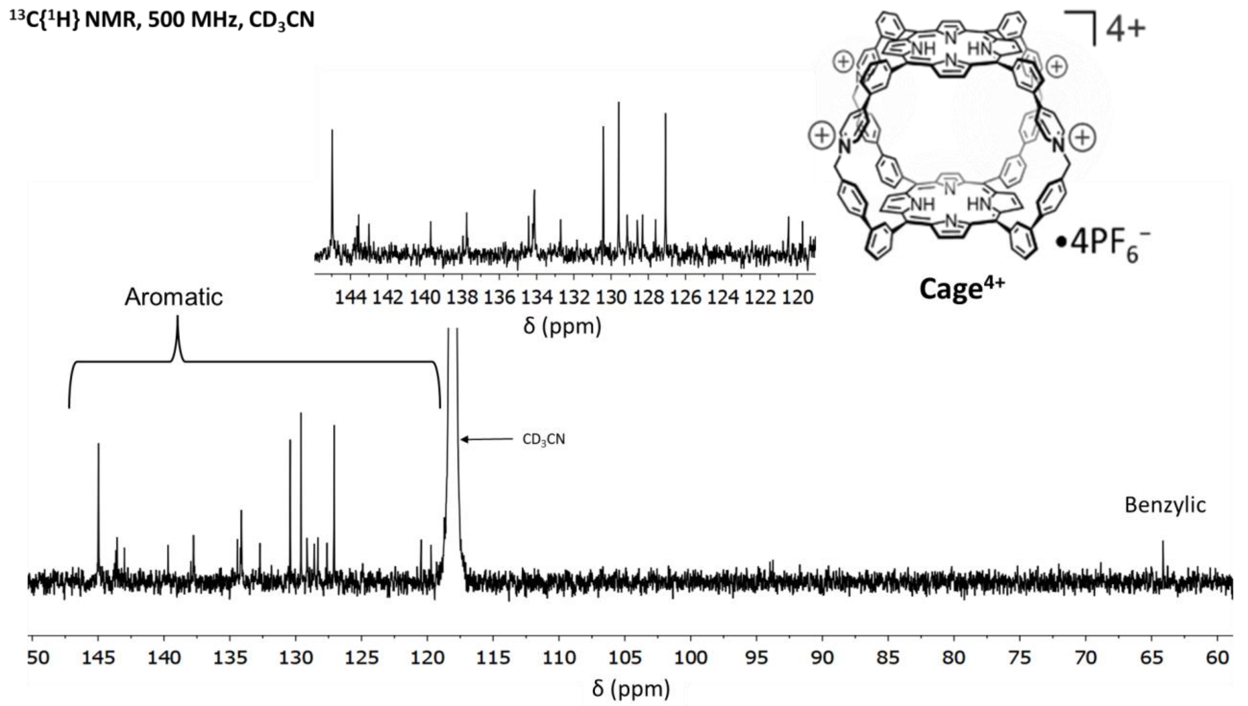

Figure S16. $^{13}C\{^{1}H\}$ NMR spectrum of **Cage**•4PF_6 in CD_3CN. Note that some resonances for quaternary carbons were not observed.

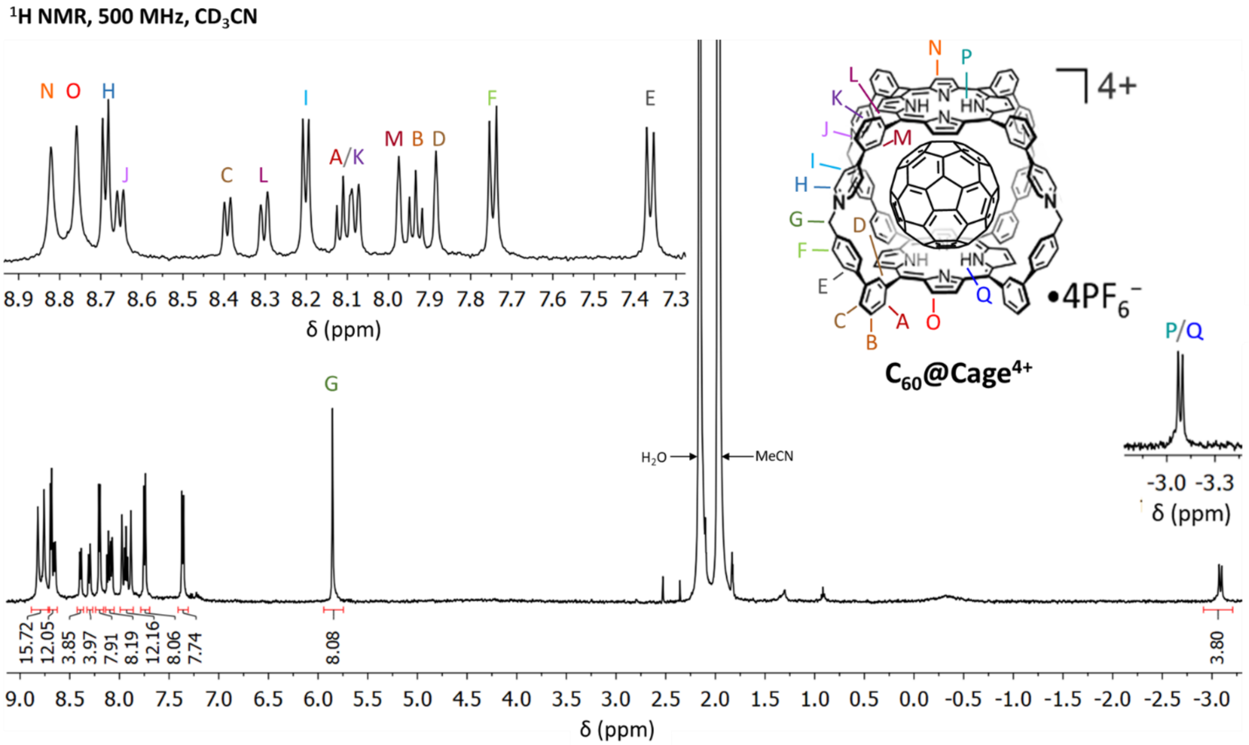

Figure S17. ^{1}H NMR spectrum of **[C_{60}@Cage]**•4PF_6 (298K, CD_3CN, 500 MHz).

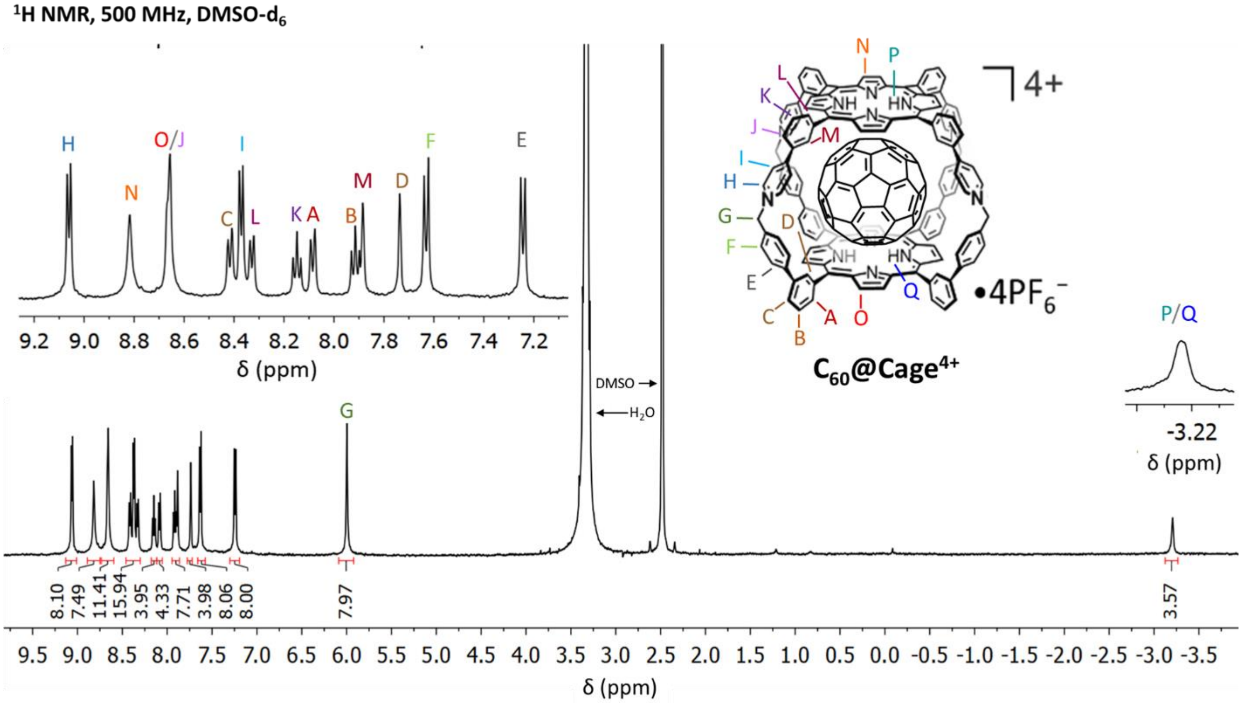

Figure S18. ^{1}H NMR spectrum of **[C_{60}@Cage]**•$4PF_6$ (298K, DMSO-d_6, 500 MHz).

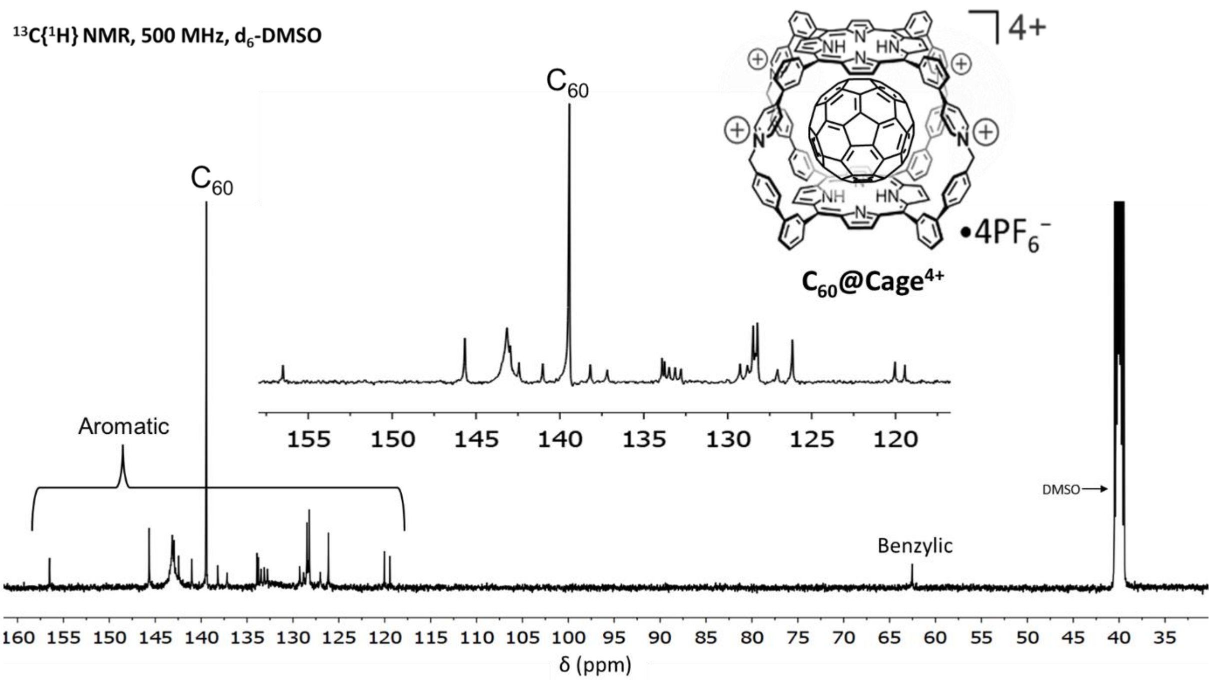

Figure S19. $^{13}C\{^{1}H\}$ NMR spectrum of **[C_{60}@Cage]**•$4PF_6$ in DMSO-d_6. Note that the resonances for the quaternary carbon resonances of the porphyrin macrocycles were not observed.

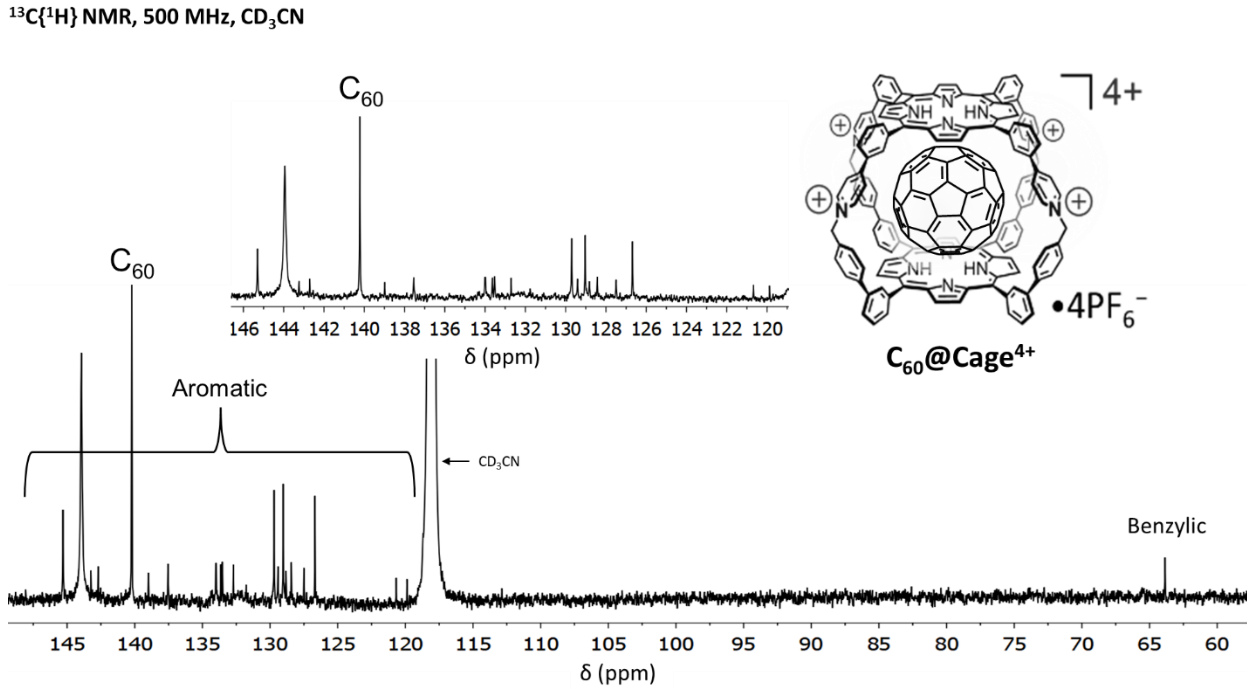

Figure S20. $^{13}C\{^1H\}$ NMR spectrum of **[C_{60}@Cage]**•$4PF_6$ in CD_3CN. Note that the resonances for the quaternary carbons of the porphyrin macrocycles were not observed.

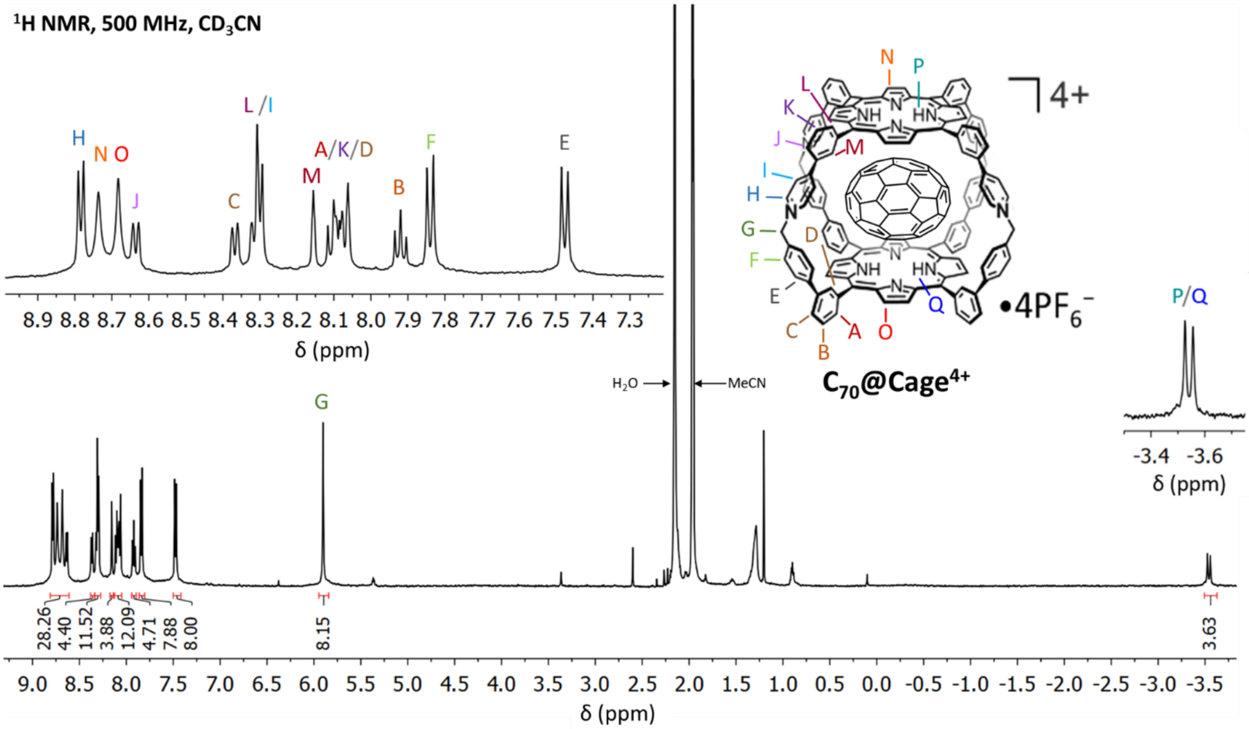

Figure S21. ^1H NMR spectrum of **[C_{70}@Cage]**•$4PF_6$ in CD_3CN (298K, 500 MHz).

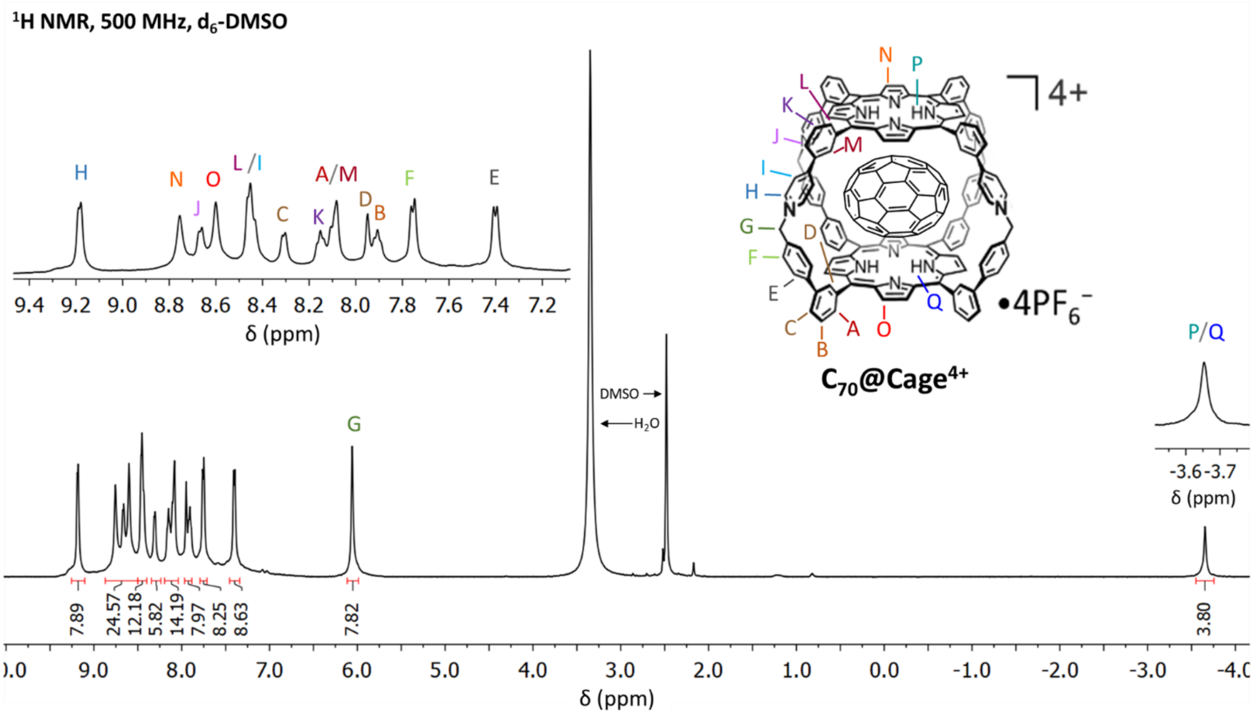

Figure S22. ^{1}H NMR spectrum of **[C$_{70}$@Cage]**•4PF$_6$ in DMSO-d$_6$ (298K, 500 MHz).

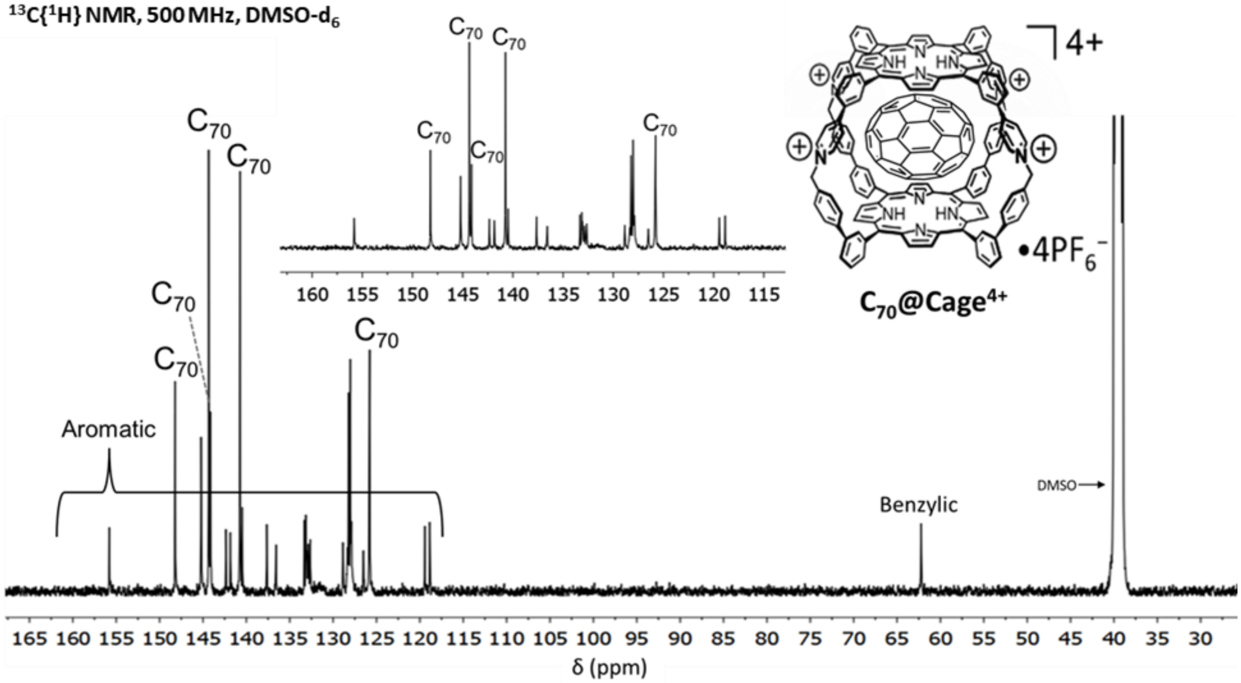

Figure S23. ^{13}C{^{1}H} NMR spectrum of **[C$_{70}$@Cage]**•4PF$_6$ in DMSO-d$_6$. Note that resonances for the quaternary carbons of the porphyrin macrocycles were not observed.

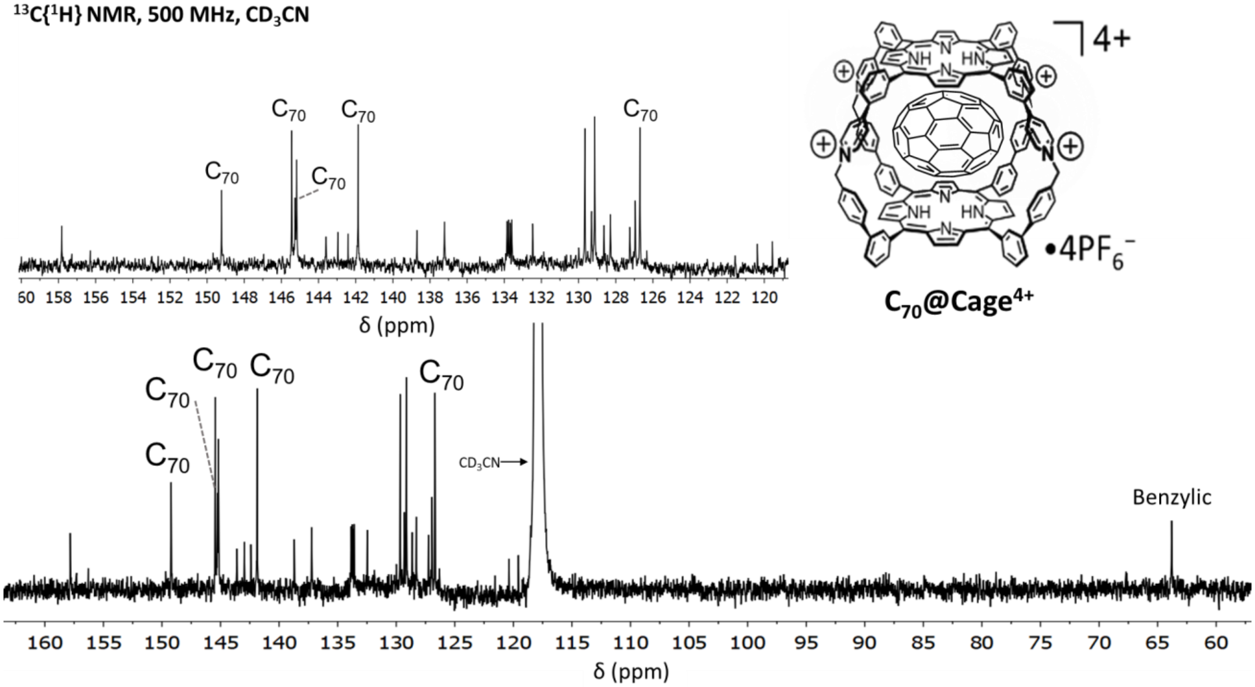

Figure S24. $^{13}C\{^{1}H\}$ NMR spectrum of **[C_{70}@Cage]**•$4PF_6$ in CD_3CN. Note that the resonances for the quaternary carbon resonances of the porphyrin macrocycles were not observed.

3b. Diffusion Ordered (DOSY) ^{1}H NMR Spectroscopy Measurements

Diffusion order spectroscopy measurements were conducted on a Bruker AVANCE Neo spectrometer using a wait time of 0.04 s. Samples were prepared at roughly 6 mM in 0.6 mL DMSO-d_6. Raw data was processed using Dynamic Center for TopSpin 4.0.7. The decay of each resonance was monitored to determine the diffusion constant (*D*) for each proton corresponding to **Cage^{4+}**. The diffusion constants of **Cage^{4+}** and host-guest complexes were determined by averaging all *D* values for proton resonances. Average diffusion constants were used in conjunction with the Stokes-Einstein equation to calculation effective hydrodynamic radii in solution.

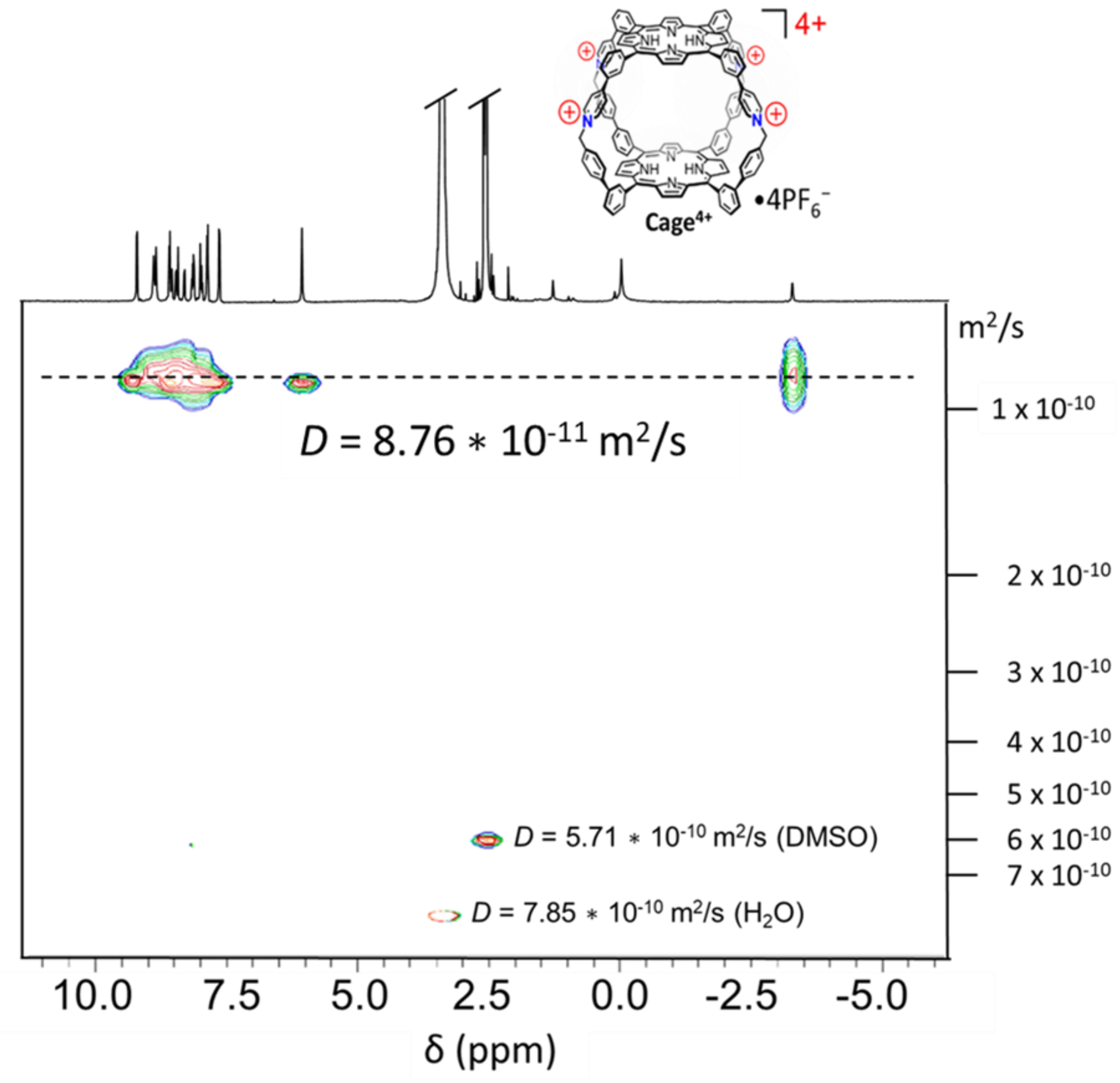

Figure S25. Diffusion ordered (DOSY) ^{1}H NMR spectrum of **Cage**•4PF_6 in DMSO-d_6. The experimental diffusion constant corresponds to a spherical hydrodynamic radius of 12.3 Å.

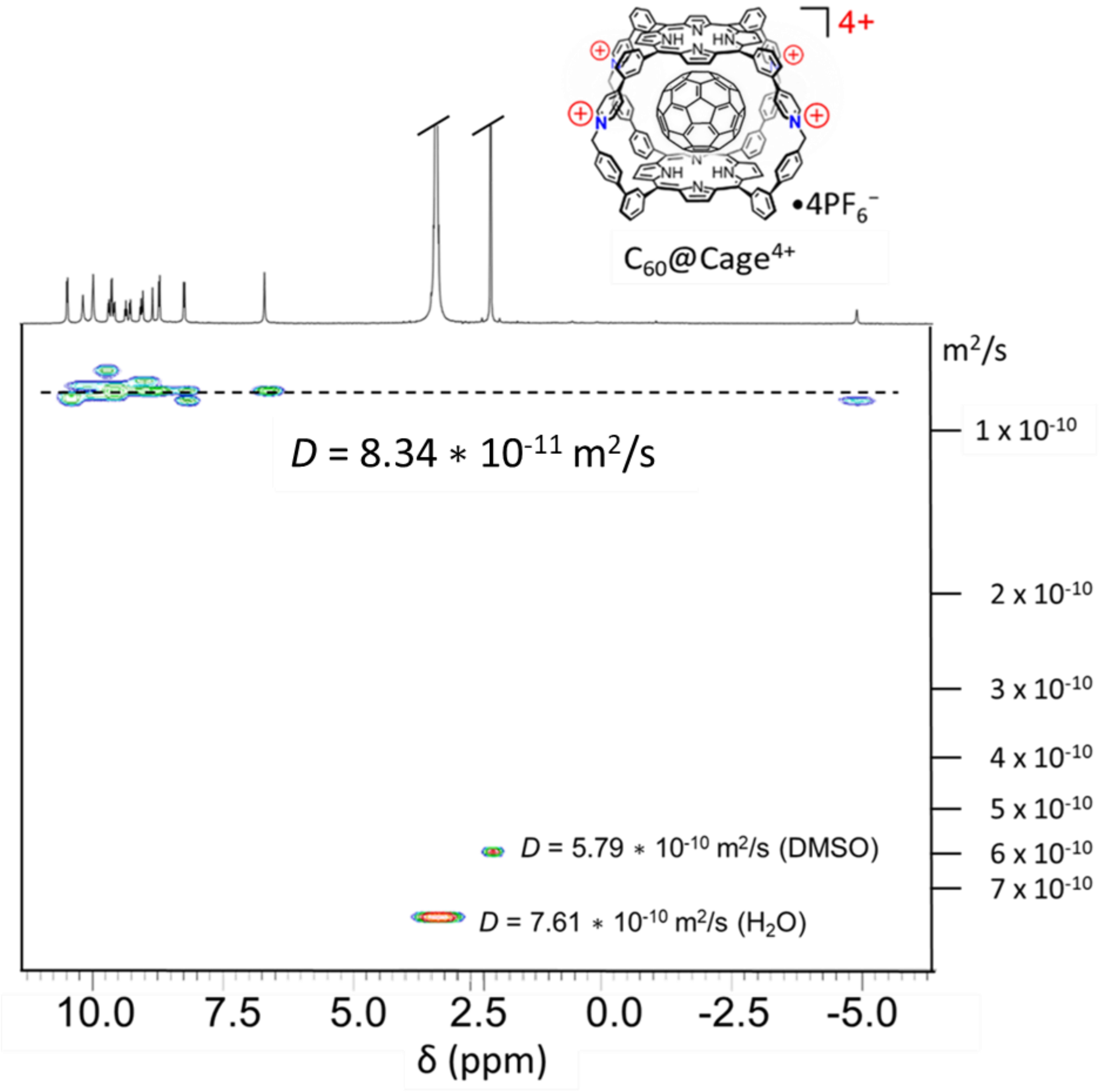

Figure S26. Diffusion ordered (DOSY) ^{1}H NMR spectrum of **[C_{60}@Cage]**•$4PF_6$ in DMSO-d_6. The experimental diffusion constant corresponds to a spherical hydrodynamic radius of 13.1 Å.

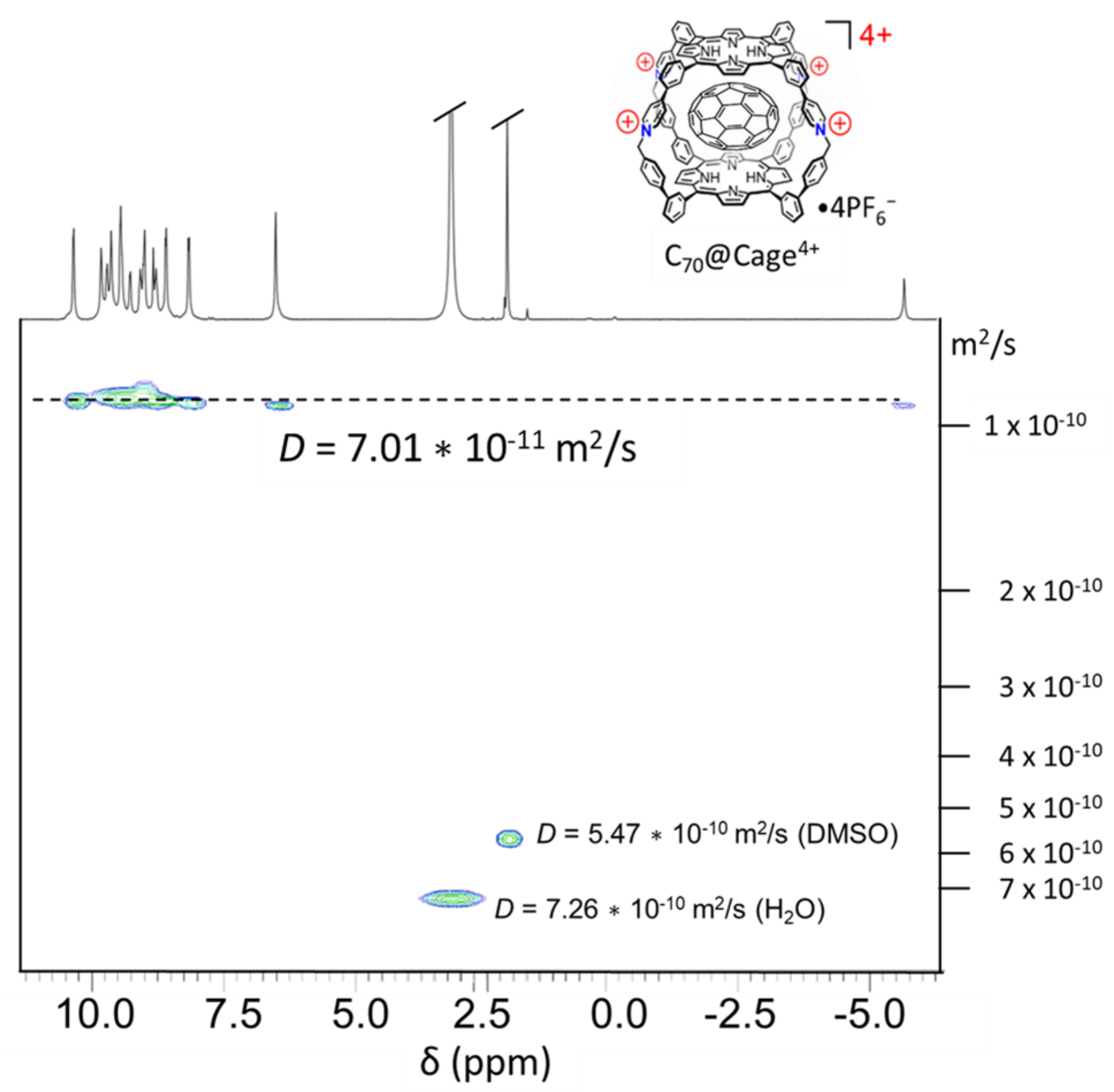

Figure S27. Diffusion ordered (DOSY) ^{1}H NMR spectrum of **[C_{70}@Cage]**•$4PF_6$ in DMSO-d_6. The experimental diffusion constant corresponds to a spherical hydrodynamic radius of 15.7 Å.

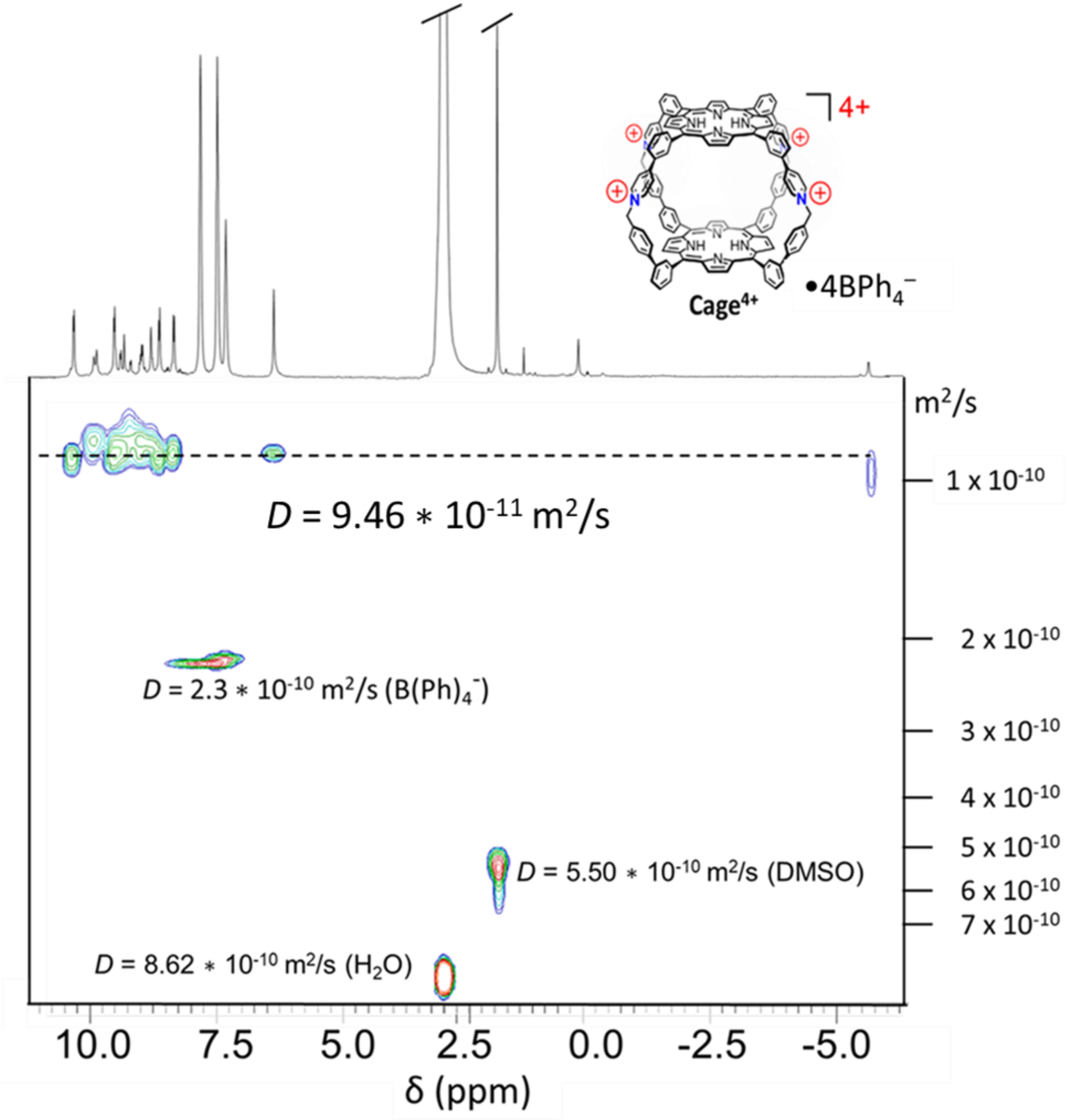

Figure S28. Diffusion ordered (DOSY) ^{1}H NMR spectrum of **Cage**•4BPh$_4$ in DMSO-d$_6$. The experimental diffusion constant corresponds to a spherical hydrodynamic radius of 11.7 Å.

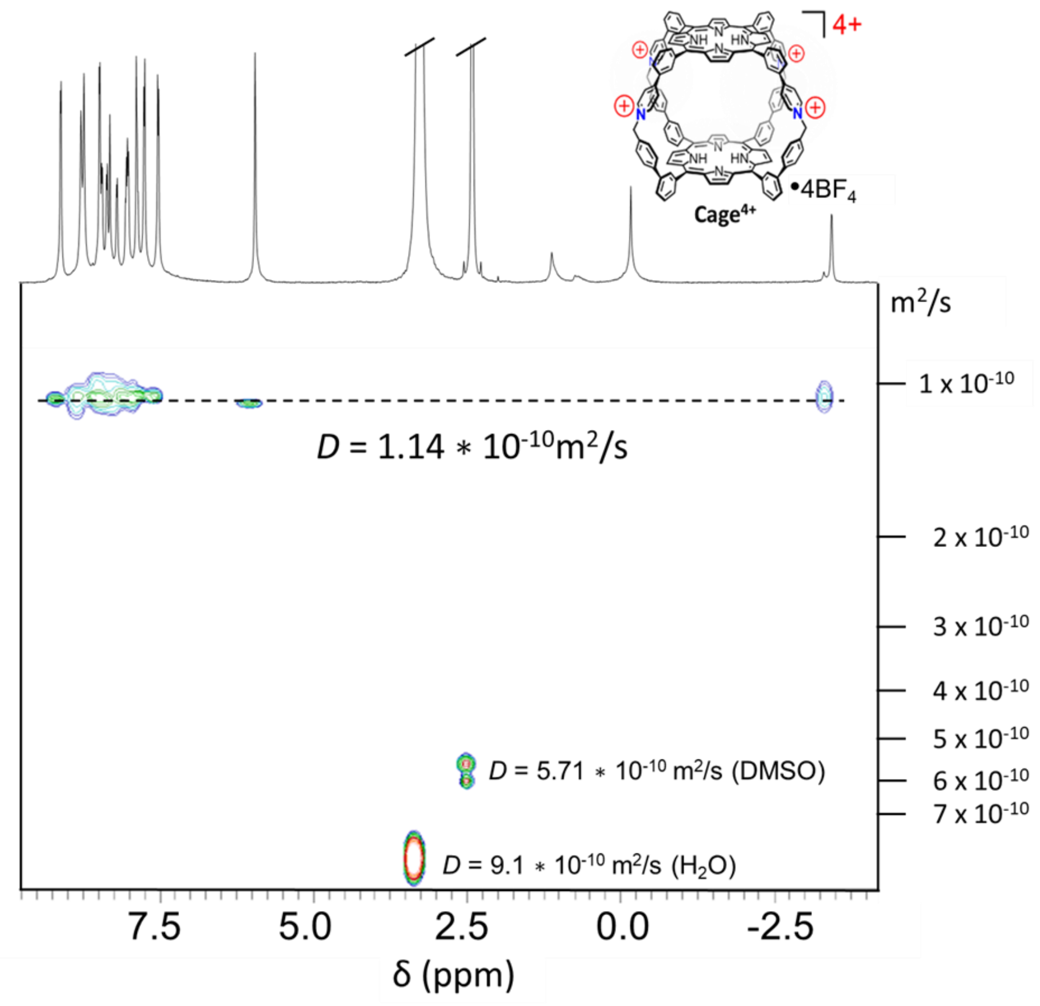

Figure S29. Diffusion ordered (DOSY) ^{1}H NMR spectrum of **Cage**•$4BF_4$ in DMSO-d_6. The experimental diffusion constant corresponds to a spherical hydrodynamic radius of 9.7 Å.

3c. NMR Binding and Competition Studies.

The association constant for the uptake of C_{60} in **Cage^{4+}** in CD_3CN was estimated by observing the complete disappearance of the ^{1}H NMR resonances of **Cage^{4+}** after sonicating an excess of the fullerene as a suspension in a saturated CD_3CN solution of **Cage**•$4PF_6$ (0.65 mM). Thus, 0.65 mM was taken as the concentration of the host-guest complex **C_{60}@Cage^{4+}**, and the reported concentration of C_{60} in acetonitrile (0.56 μM) was taken as the concentration of free C_{60} in solution. Serial dilutions of a CD_3CN solution of **Cage**•$4PF_6$ were used to determine a limit of ^{1}H NMR detection of 1.94 μM for the cage, which was taken as the maximum possible concentration of free host in solution under experimental conditions. Together, these concentrations provide an association constant of ≥6.0 x 10^{8} M^{-1} for **C_{60}@Cage^{4+}** in acetonitrile.

Binding competition experiments between C_{60} and C_{70} were performed by tracking the ^{1}H NMR resonances of empty **Cage^{4+}**, **C_{60}@Cage^{4+}**, and **C_{70}@Cage^{4+}** in CD_3CN after sonicating a 10:1 mixture of the fullerenes in a CD_3CN solution of the host (see Figure S30).

The association of the fulleride anions C_{60}^{-} and C_{60}^{2-} in **Cage^{4+}** was measured by titrating DMF solutions of the $Cp*_2Co^+$ salts of the fullerides into solutions of **Cage**•4PF_6 in CD_3CN under an N_2 atmosphere. Despite signal broadening caused by the paramagnetic guests, new ^{1}H NMR resonances for the host-guest complexes were clearly observable as distinct from those of the empty host (Figures S31 and S32), indicating slow exchange of the guests. The benzylic CH resonances of the host and host-guest complexes were used for determining the ratio of empty vs. occupied host. The total concentration of the fulleride guest in solution was determined by integration of the resonance of the $Cp*_2Co^+$ countercations (1.7 ppm), and the concentration of free guest was determined by subtracting the concentration of host-guest complex. The complex [**C_{60}^{2-}@Cage**]•2PF_6 has a reduced solubility in the CD_3CN, resulting in some precipitation of this complex during the titration, so a sealed capillary of ferrocene in CD_3CN was used as an internal standard to provide accurate quantification of the concentration of the host-guest complex over the course of the titration. Association constants for C_{60}^{-} and C_{60}^{2-} in **Cage^{4+}** were determined by integration of the spectra at the mid-point of each titration, at which point the concentrations of free and occupied host are nearly equal.

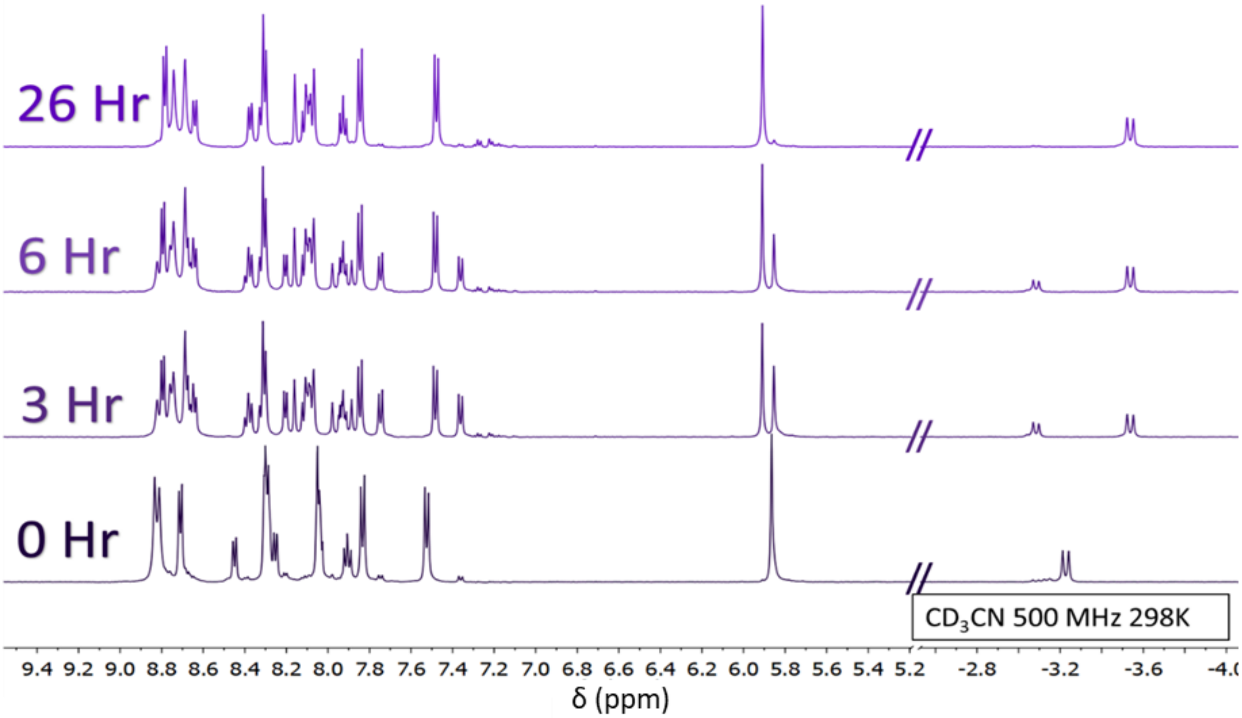

Figure S30. ^{1}H NMR spectra of a saturated solution of **Cage**•4PF_6 after adding an excess of C_{60} and C_{70} (10:1 ratio) and sonicating for the indicated times. A mixture of **[C_{60}@Cage^{4+}]** and **[C_{70}@Cage^{4+}]** is initially formed, but converts almost completely to **[C_{70}@Cage^{4+}]** after 26 h of sonication.

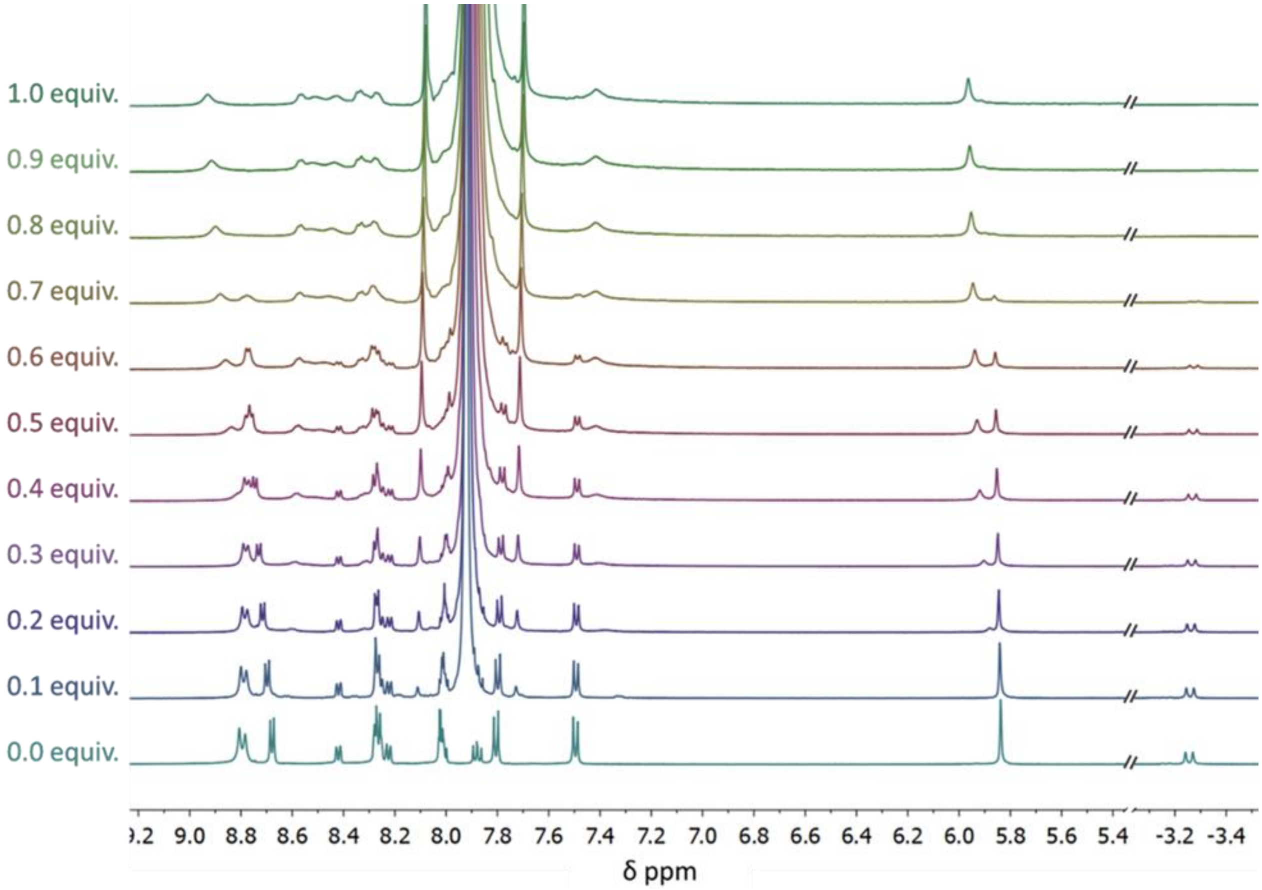

Figure S31. Titration of a DMF solution of C_{60}^{-} (4.04 mM) as its $Cp^*_2Co^+$ salt into a solution of **Cage**•$4PF_6$ in CD_3CN (0.673 mM, 0.404 μmol in 0.6 mL CD_3CN).

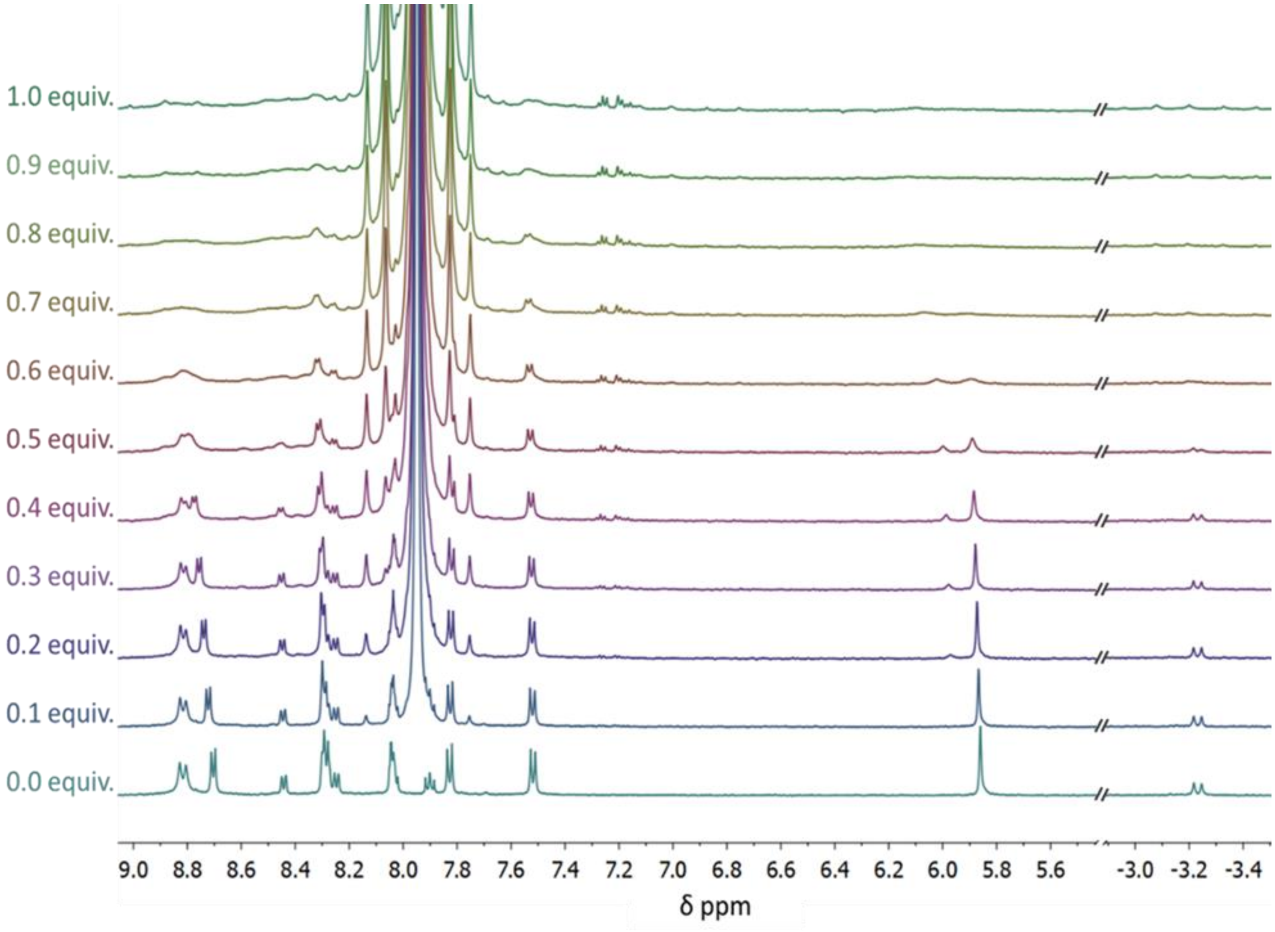

Figure S32. Titration of a DMF solution of C_{60}^{2-} (4.04 mM) as its $Cp^{*}_2Co^+$ salt into a solution of **Cage^{4+}** in CD_3CN (0.673 mM, 0.404 µmol in 0.6 mL CD_3CN).

4. Mass Spectrometry

4a. ESI-MS characterization of Cage^{4+} and Host-Guest Complexes

High-resolution mass spectra were obtained using a Waters Xevo G2-XS QTof mass spectrometer using an ESI source and positive ion detection. Samples of the host-guest complexes in MeCN were introduced by direct infusion (5 µL/min). A standard of Leu-Enkephalin (Waters, Milford, MA) was injected in tandem with the sample for in-process calibration. In general, capillary and sample cone voltages were kept between 2 and 3 V, and 120 and 140 V, respectively, to optimize signal to noise ratio for free **Cage^{4+}** and host-guest species. Trifluoroacetic acid was included in samples as an H^+ source for acquiring the mass spectra of neutral porphyrins.

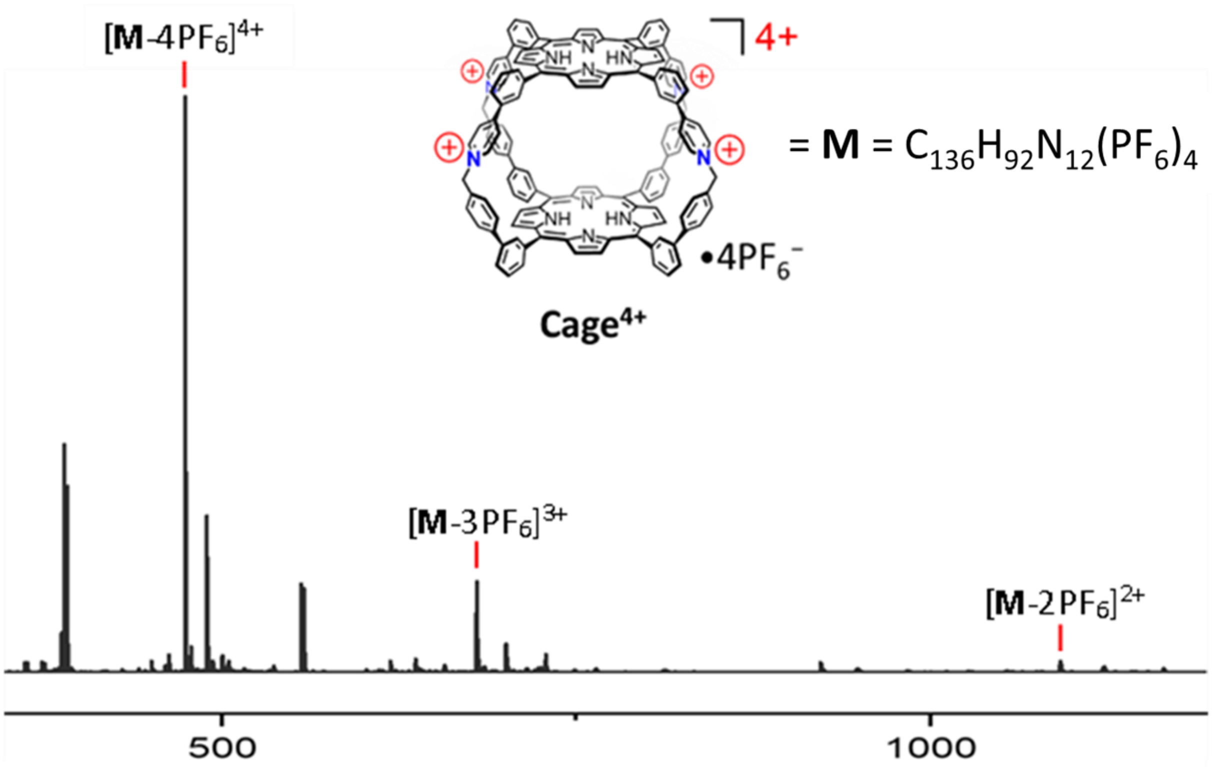

Figure S33. ESI(+)-MS spectrum of **Cage**•4PF$_6$ in CH$_3$CN showing successive loss of PF$_6^-$ from **Cage^{4+}**.

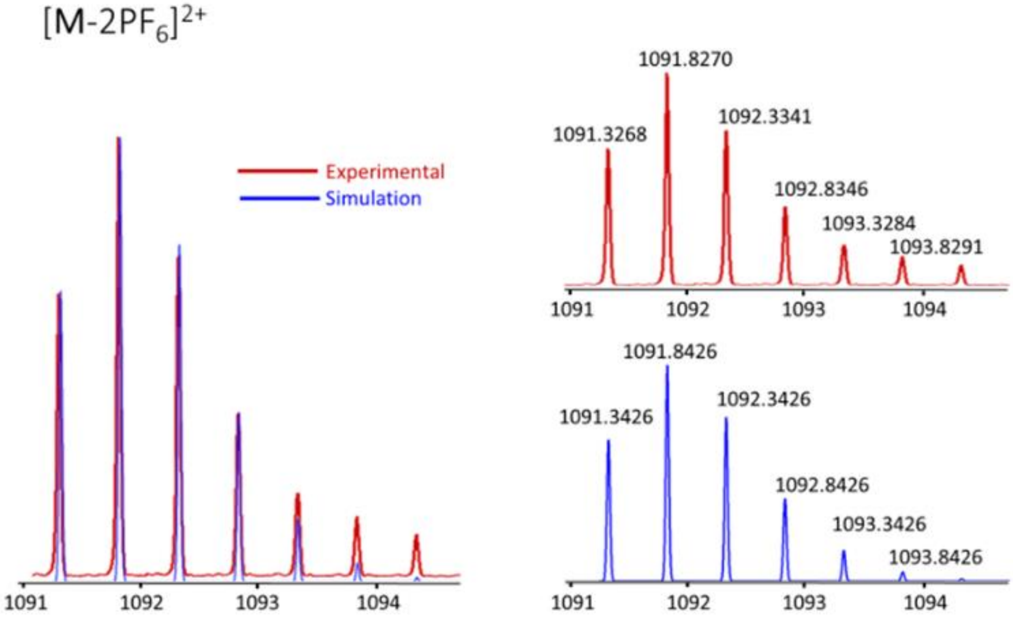

Figure S34. Partial ESI(+)-MS spectrum of **Cage**•4PF$_6$ in CH$_3$CN showing the loss of two PF$_6$ anions, resulting in the species [**Cage**•2PF$_6$]$^{2+}$.

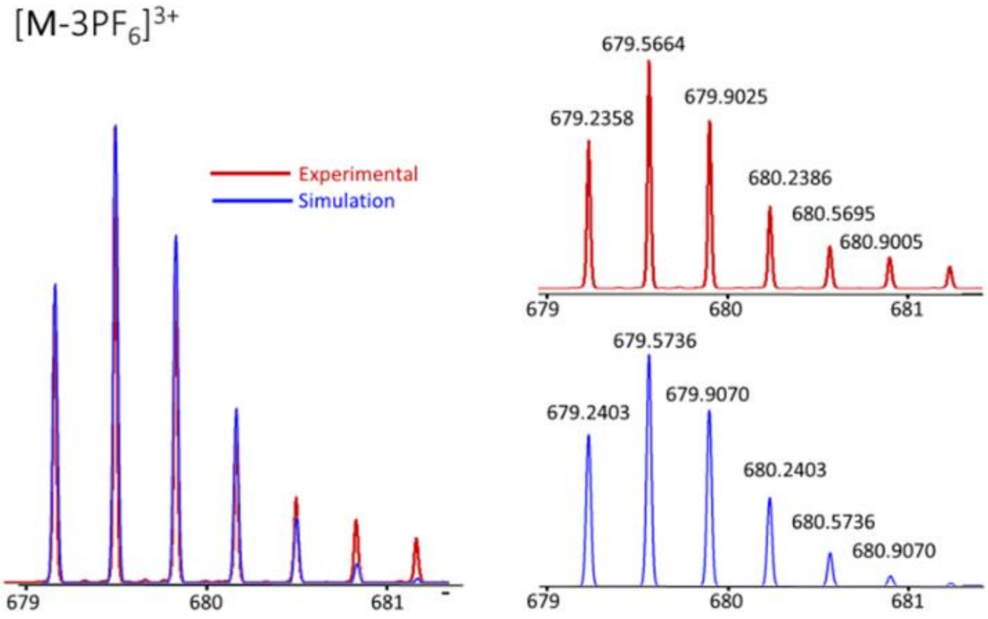

Figure S35. Partial ESI(+)-MS spectrum of **Cage**•4PF_6 in CH_3CN showing the loss of three PF_6 anions, resulting in the species [**Cage**•PF_6]$^{3+}$.

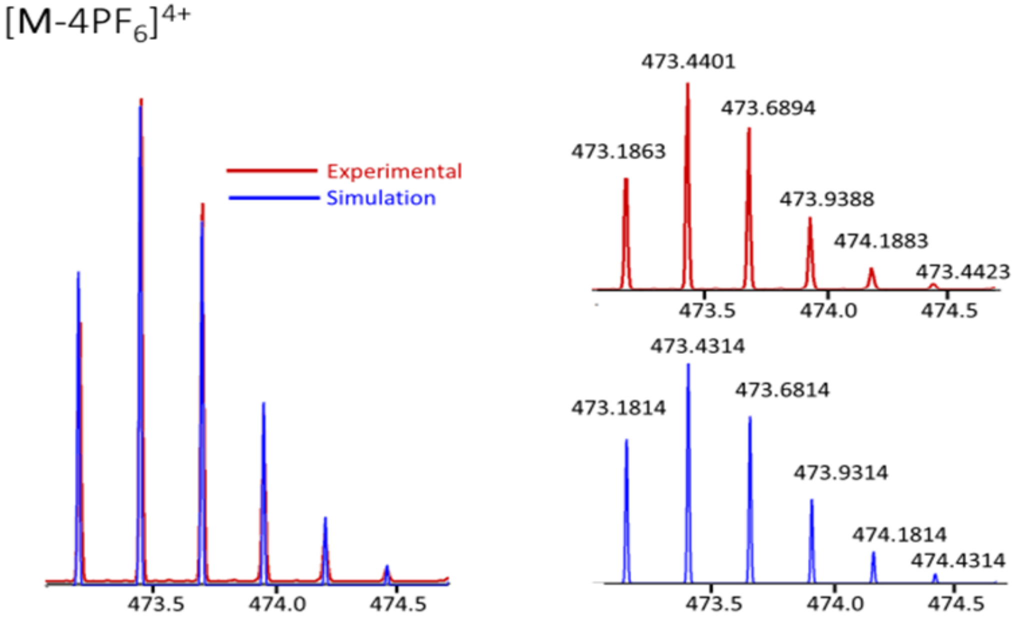

Figure S36. Partial ESI(+)-MS spectrum of **Cage**•4PF_6 in CH_3CN showing the loss of four PF_6 anions, resulting in the species [**Cage**]$^{4+}$.

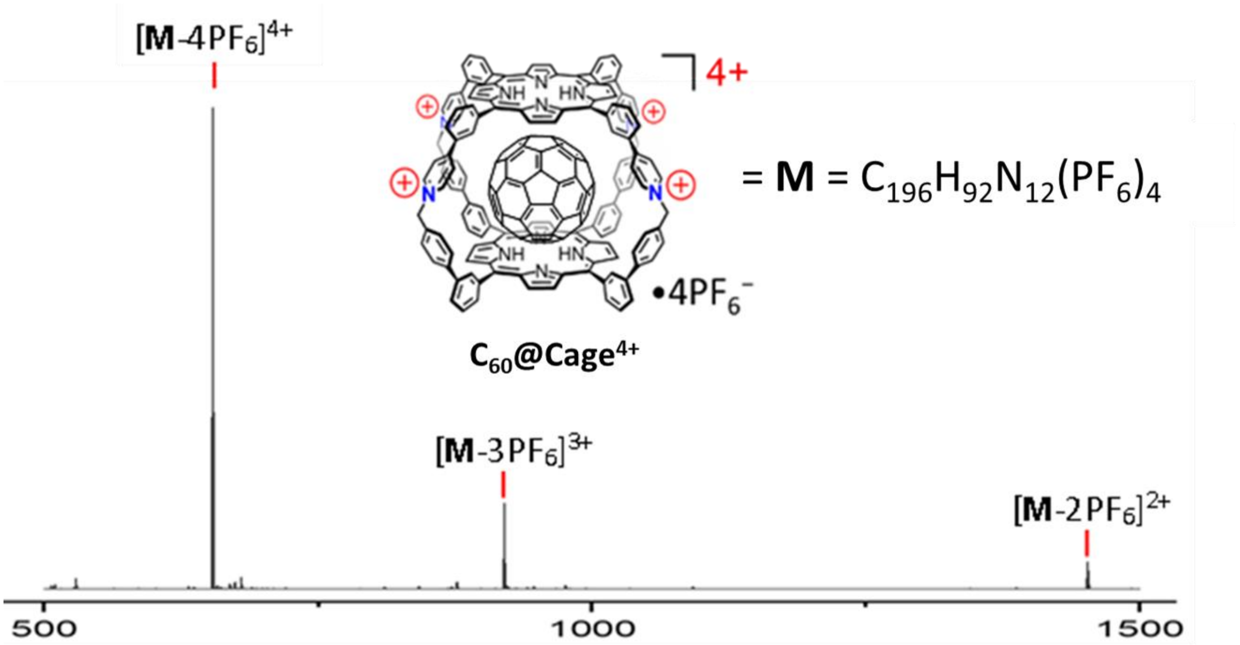

Figure S37. ESI(+)-MS spectrum of **C_{60}@Cage**•4PF_6 in CH_3CN showing successive loss of PF_6 anions from the host-guest complex.

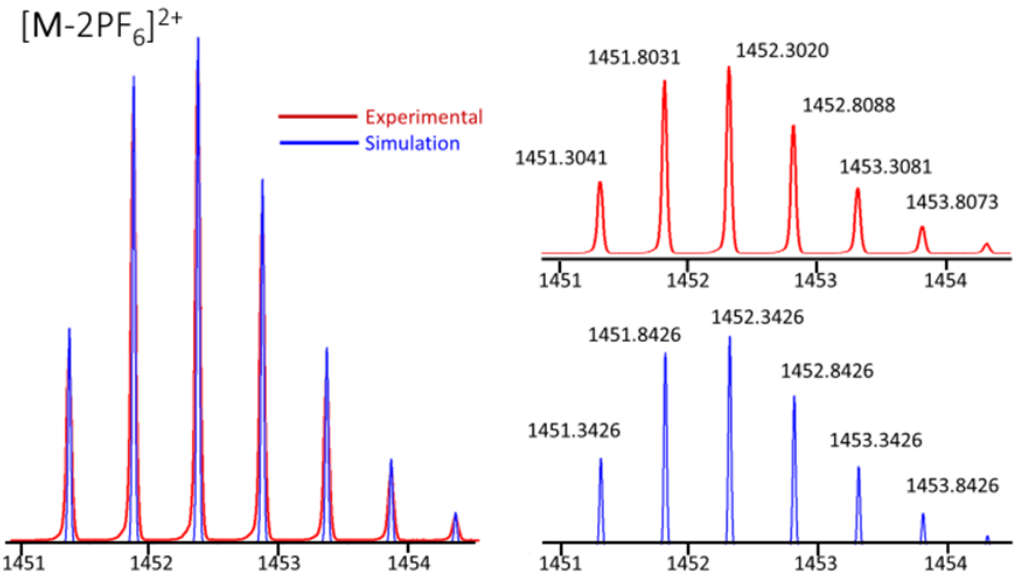

Figure S38. Partial ESI(+)-MS spectrum of **[C_{60}@Cage]**•4PF_6 in CH_3CN showing the loss of two PF_6 anions, resulting in the species **[C_{60}@Cage**•2PF_6**]**$^{2+}$.

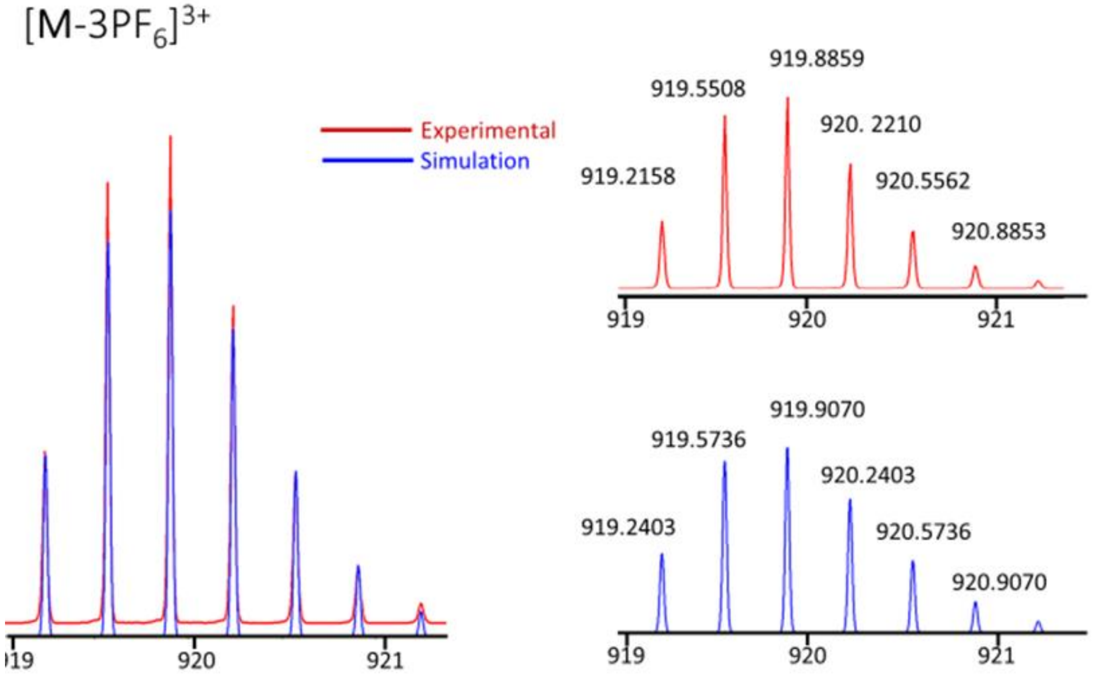

Figure S39. Partial ESI(+)-MS spectrum of **[C_{60}@Cage]**•4PF_6 in CH_3CN showing the loss of three PF_6 anions, resulting in the species **[C_{60}@Cage•**PF_6 **]**$^{3+}$.

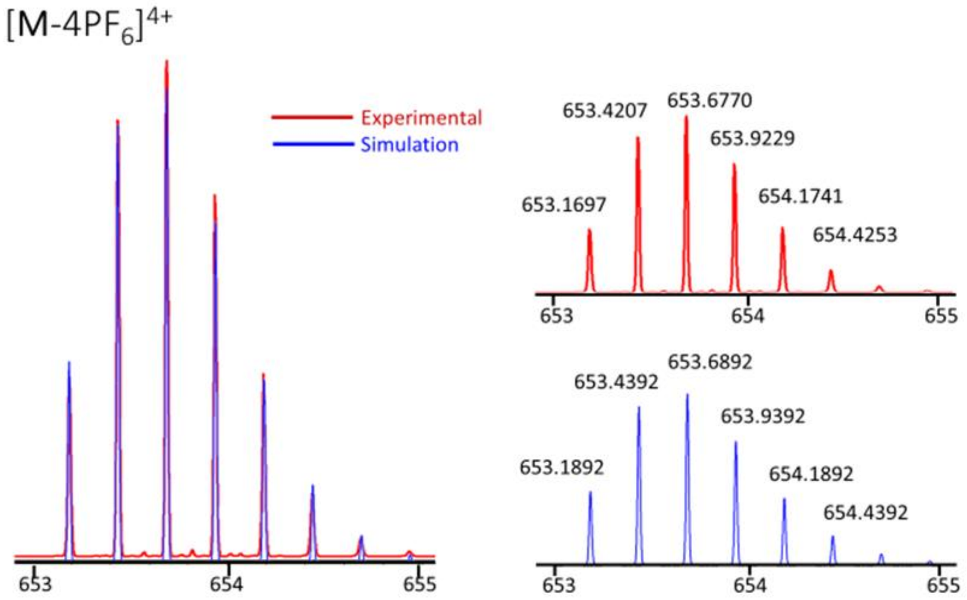

Figure S40. Partial ESI(+)-MS spectrum of **[C_{60}@Cage]**•4PF_6 in CH_3CN showing the loss of four PF_6 anions, resulting in the species [**C_{60}@Cage**]$^{4+}$.

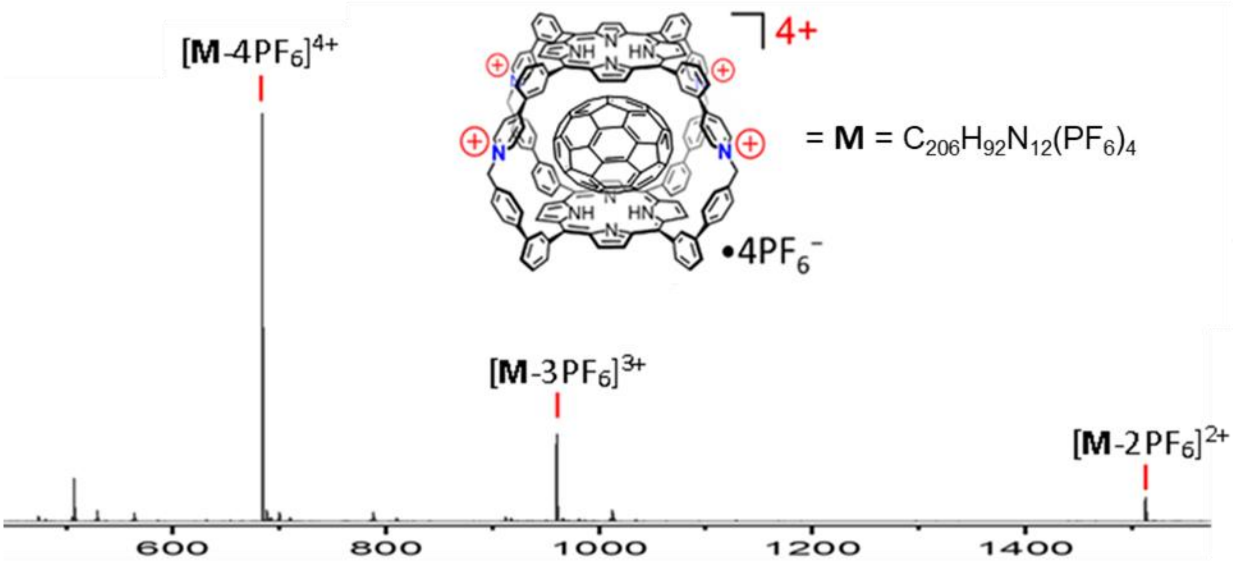

Figure S41. ESI(+)-MS spectrum of **[C_{70}@Cage]**•4PF_6 in CH_3CN showing successive loss of PF_6 anions from the host-guest complex.

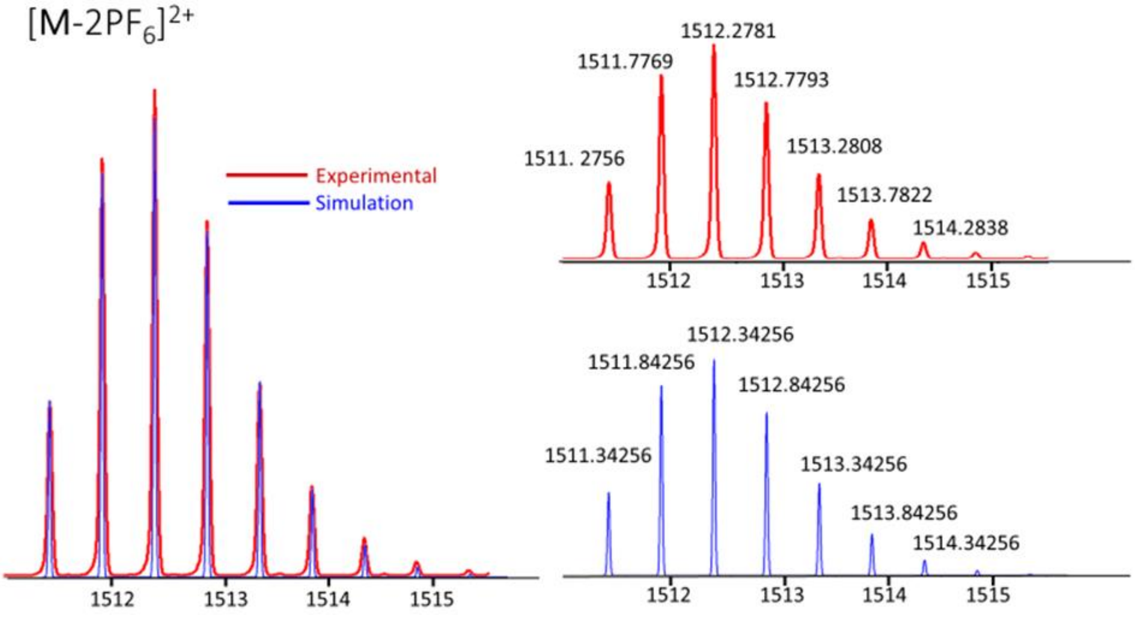

Figure S42. Partial ESI(+)-MS spectrum of **[C_{70}@Cage]**•4PF_6 in CH_3CN showing the loss of two PF_6 anions, resulting in the species [**C_{70}@Cage**•2PF_6]$^{2+}$.

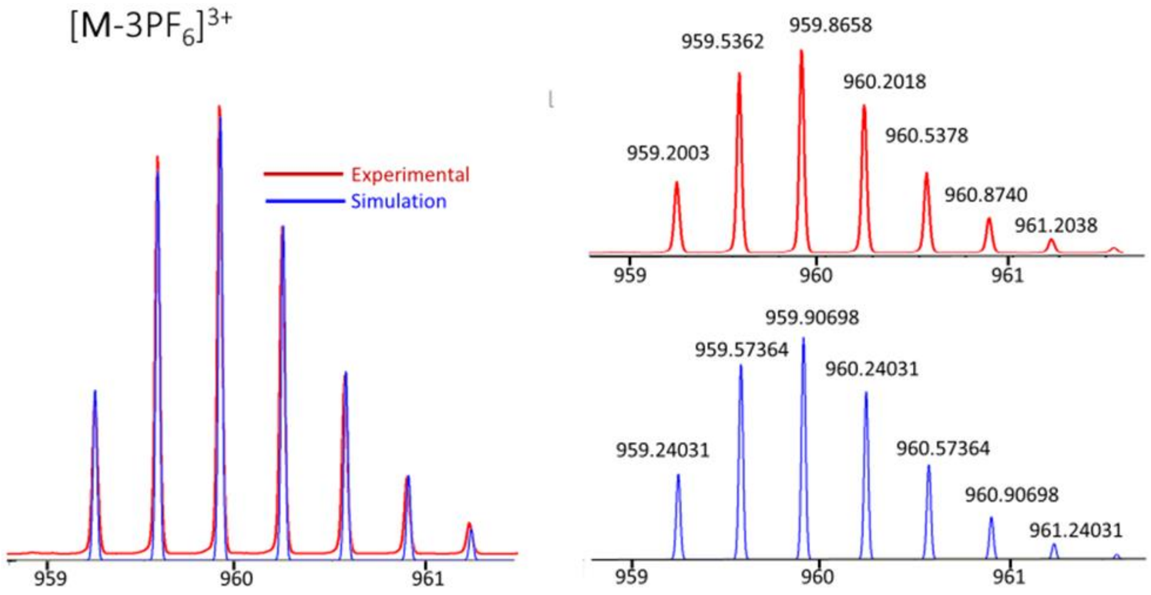

Figure S43. Partial ESI(+)-MS spectrum of **C_{70}@Cage**•4PF_6 in CH_3CN showing the loss of three PF_6 anions, resulting in the species [**C_{70}@Cage**•PF_6]$^{3+}$.

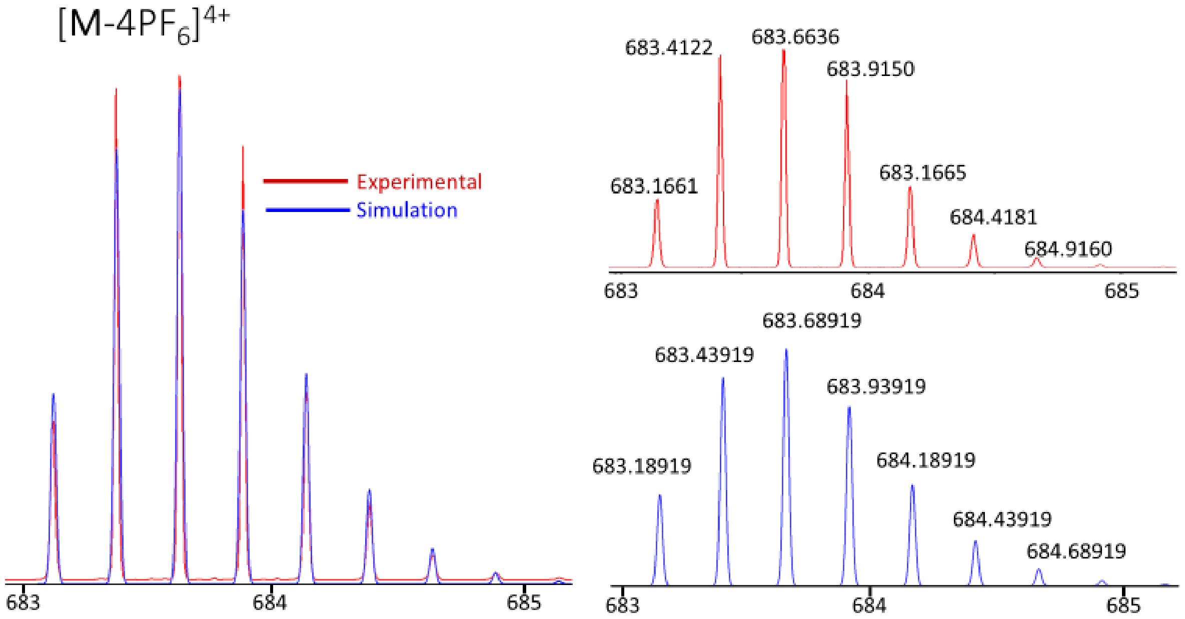

Figure S44. Partial ESI(+)-MS spectrum of **[C_{70}@Cage]**•4PF_6 in CH_3CN showing the loss of four PF_6 anions, resulting in the species [**C_{70}@Cage**]$^{4+}$.

4b. Travelling Wave Ion Mobility (TWIM) Mass Spectrometry

TWIM-MS was performed on a Waters SYNAPT G2-SI qTOF instrument using a ion mobility separation (IMS) wave height of 43.1 V and a wave velocity of 4.6 m/s in a helium collision cell. Data was analyzed and figures were prepared using Drift Scope.

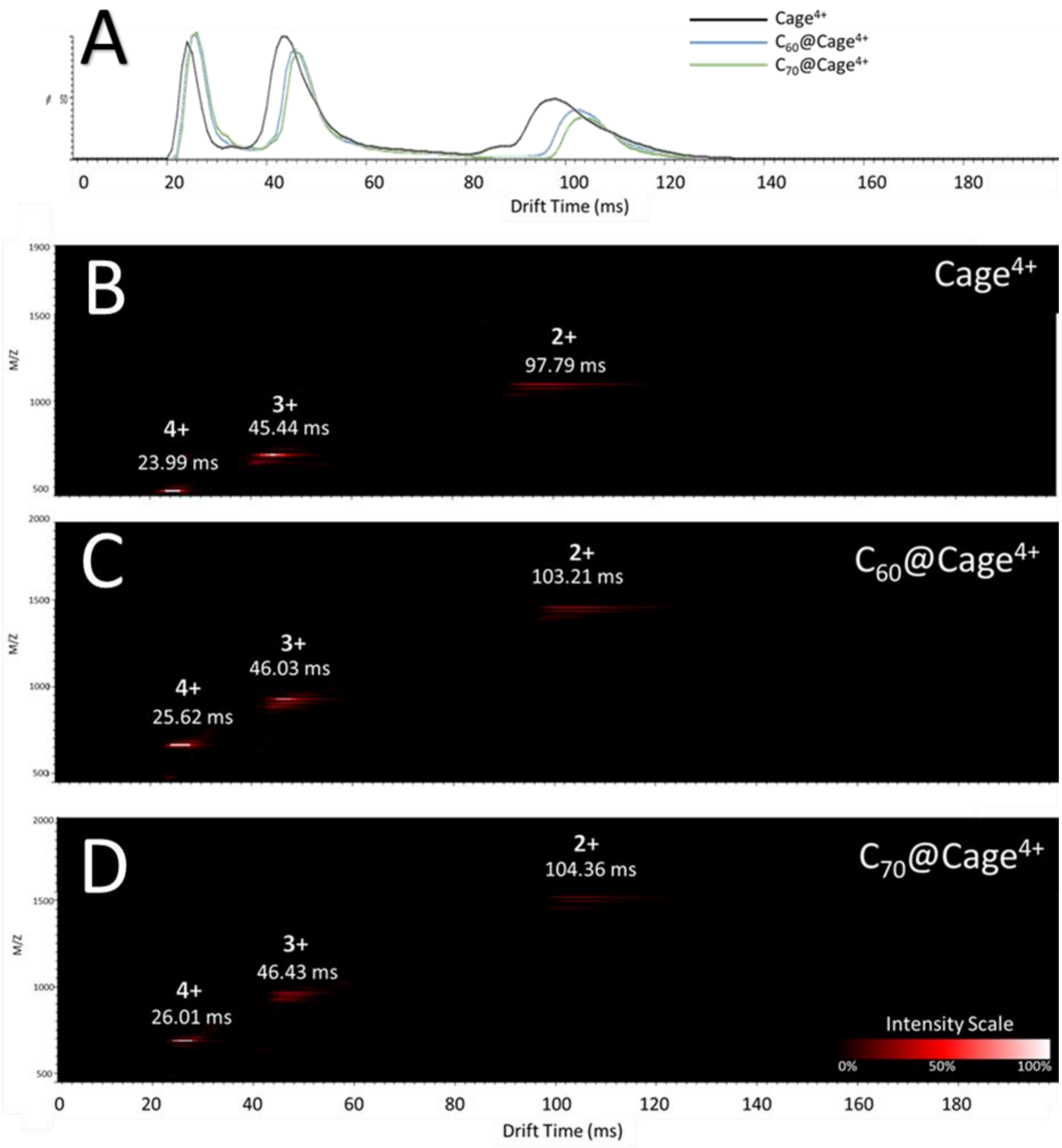

Figure S45. (A) Travelling wave chromatograms showing drift times for **Cage^{4+}** (black), **C_{60}@Cage^{4+}** (blue), and **C_{70}@Cage^{4+}** (green). The host-guest complexes exhibit slightly longer drift times than the unfilled host, indicating a slightly larger cross-sectional area for these complexes. The identity of the ions entering the detector was confirmed by (B-D) the correlation of the M/Z values to the corresponding drift times.

5. Electrochemical Studies

Cyclic voltammetry measurements were recorded using a CH Instruments 600E potentiostat. Dry DMF was used as the solvent, and 0.1 M tetrabutylammonium hexafluorophosphate ($TBAPF_6$) was used as a supporting electrolyte. All measurements were recorded using a 3 mm glassy carbon working electrode, a platinum wire counter electrode, and a silver wire pseudo-reference electrode which was confined in a polypropylene body that provided contact with the analyte solution via a porous zeolite bead or a glass frit. Potentials were referenced to the $Fc^{+/0}$ couple of an internal ferrocene standard or by calibrating the pseudo-reference electrode vs. the $Fc^{+/0}$ couple of an external solution of ferrocene immediately before use (note: keeping the pseudo-reference electrode isolated from the bulk sample prevents significant drift of the reference potential over numerous CV experiments). For clarity, we present the CVs that do not include the $Fc^{+/0}$ wave. Positive feedback iR compensation was applied during all CV experiments. The 2nd cycle out of three is presented unless otherwise noted.

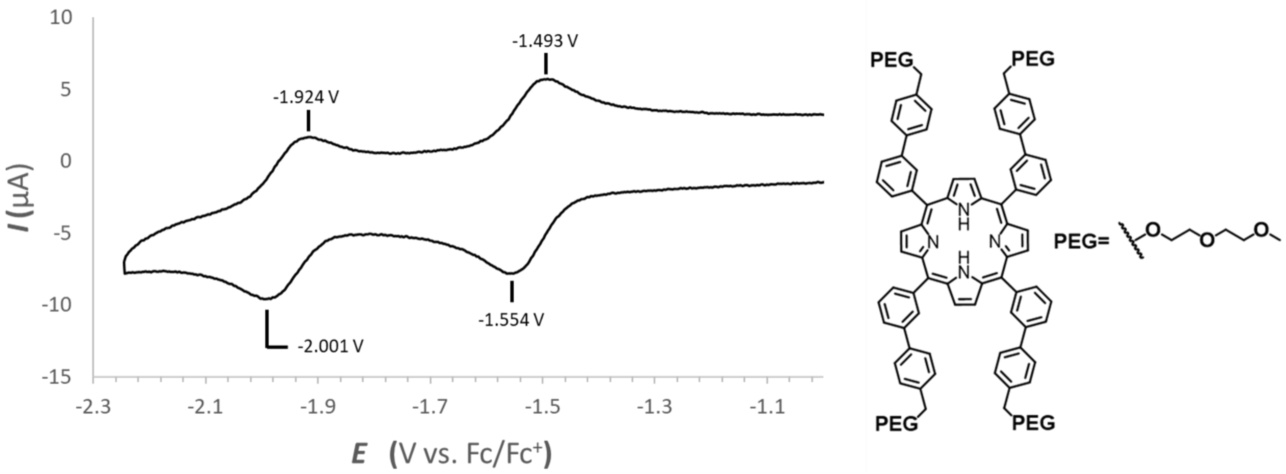

Figure S46. Cyclic voltammogram of tetrakis-3-(4-(2-(2-methoxyethoxy)ethoxymethyl)phenyl)phenyl-porphyrin (0.1 mM) recorded at 2 V/s in DMF. The two reversible redox couples correspond to porphyrin-centered 1 e^- redox processes ($E_{1/2}$ = -1.52 V, -1.96 V vs. $Fc^{+/0}$).

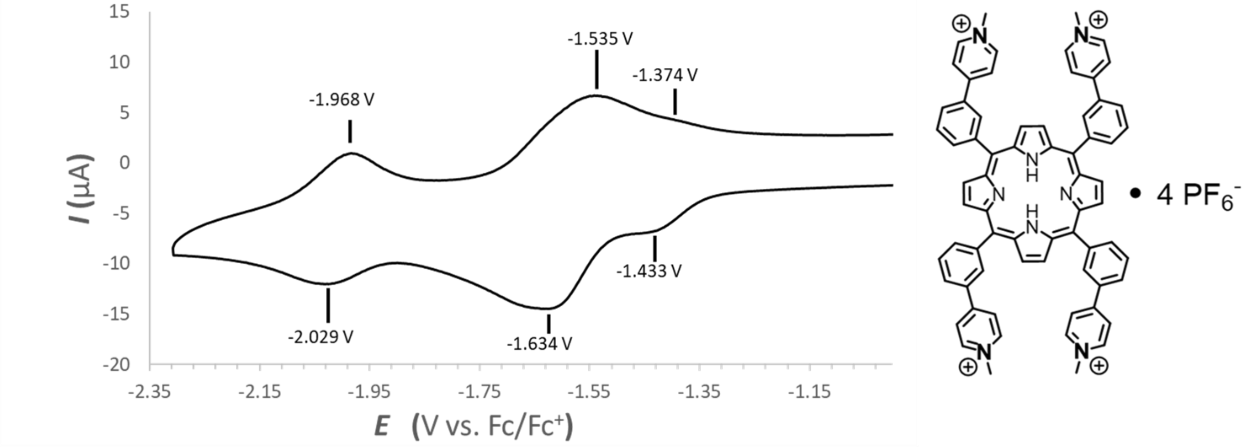

Figure S47. Cyclic voltammogram of [tetrakis-3-(*N*-methyl-4-pyridyl)phenylporphyrin]•4PF_6 (0.1 mM) recorded at a scan rate of 2 V/s in DMF. Reversible porphyrin-centered reductions occur at $E_{1/2}$ = -1.40 V and -2.00 V vs $Fc^{0/+}$, while the *N*-methyl-4-pyridinium groups exhibit a quasireversible redox couple at $E_{1/2}$ = -1.58 V that partially overlaps with the more positive porphyrin-centered redox feature.

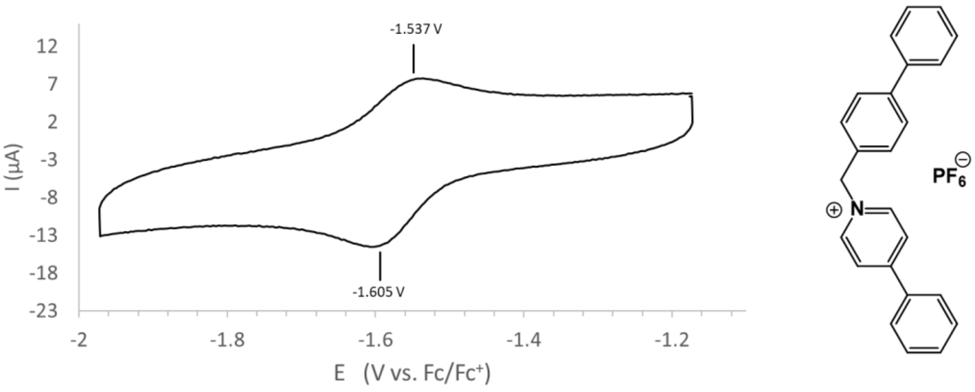

Figure S48. Cyclic voltammogram of [*N*-(4-phenylbenzyl)-4-phenylpyridinium]PF_6 (0.4 mM) recorded in DMF at a scan rate of 2 V/s.

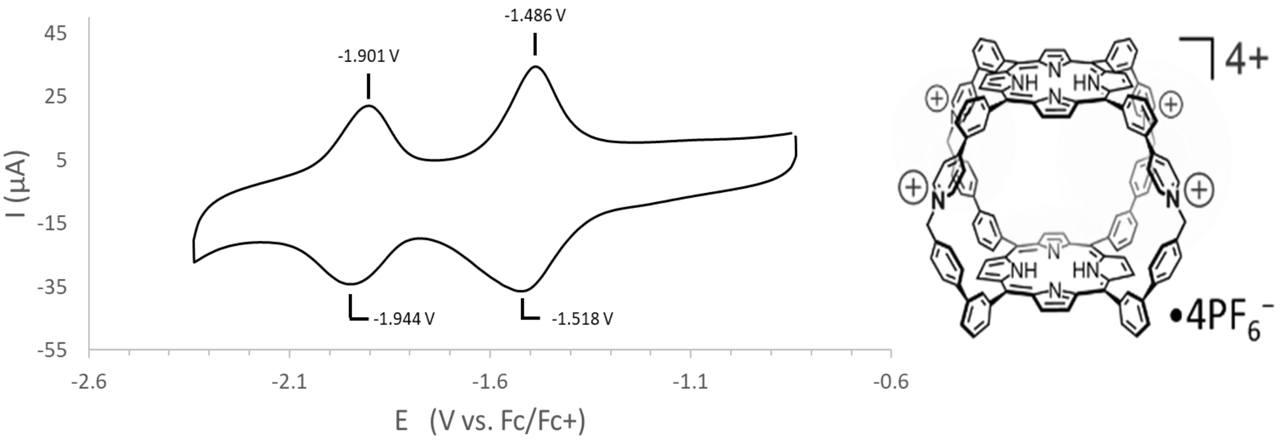

Figure S49. Cyclic voltammogram of **Cage**•4PF_6 (0.1 mM) recorded at a scan rate of 2 V/s in DMF. See main text for discussion of redox features.

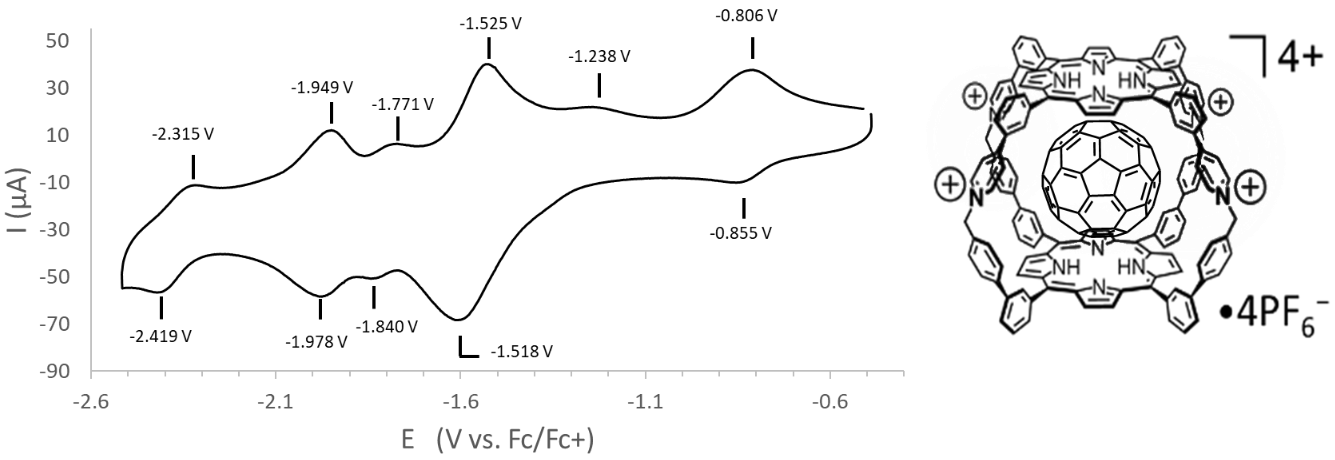

Figure S50. Cyclic voltammogram of **[C_{60}@Cage]**•4PF_6 (0.1 mM) recorded at a scan rate of 2 V/s in DMF. See main text for discussion of redox features.

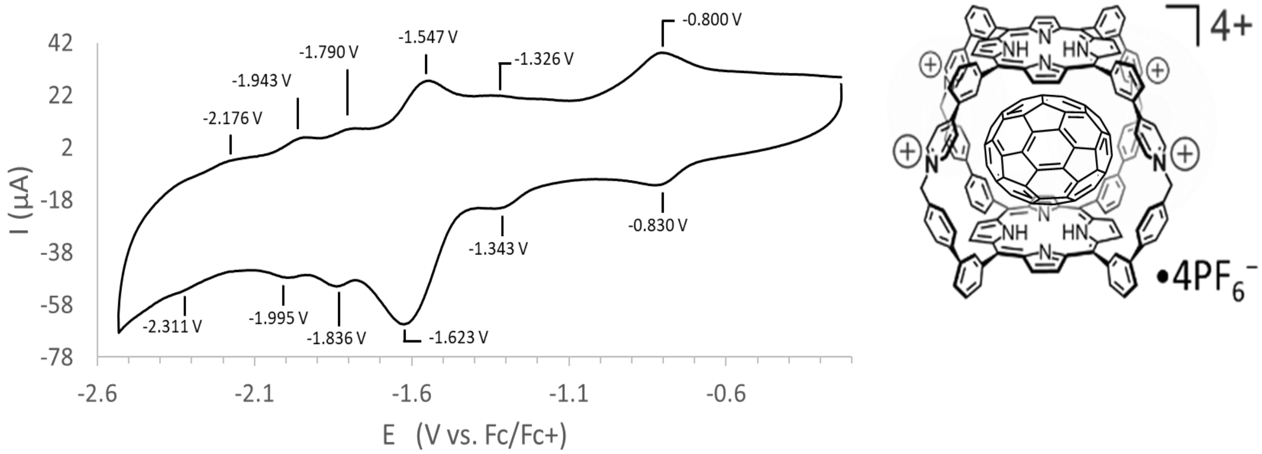

Figure S51. Cyclic voltammogram of **[C_{70}@Cage]**•4PF_6 (0.1 mM) recorded at a scan rate of 2 V/s in DMF. See main text for discussion of redox features.

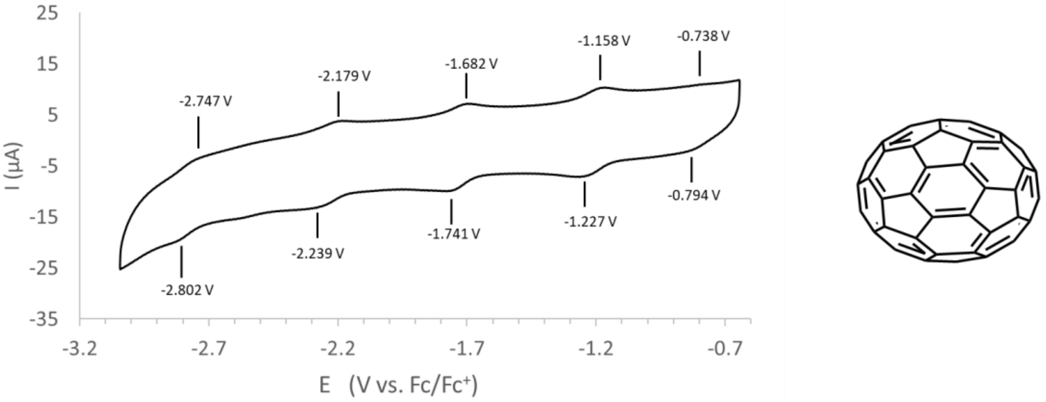

Figure S52. Cyclic voltammogram of C_{70}^- (0.1 mM) recorded at a scan rate of 2V/s in DMF. Based on a reported procedure for analyzing the redox features of C_{60} in polar solvents,[9] the soluble C_{70}^- anion was prepared in situ via controlled-potential electrolysis (E_{app} = −1.07 V vs $Fc^{0/+}$) on a stirred suspension of C_{70} in DMF. The cyclic voltammogram was then recorded at a fast enough scan rate to avoid precipitation of the insoluble neutral fullerene.

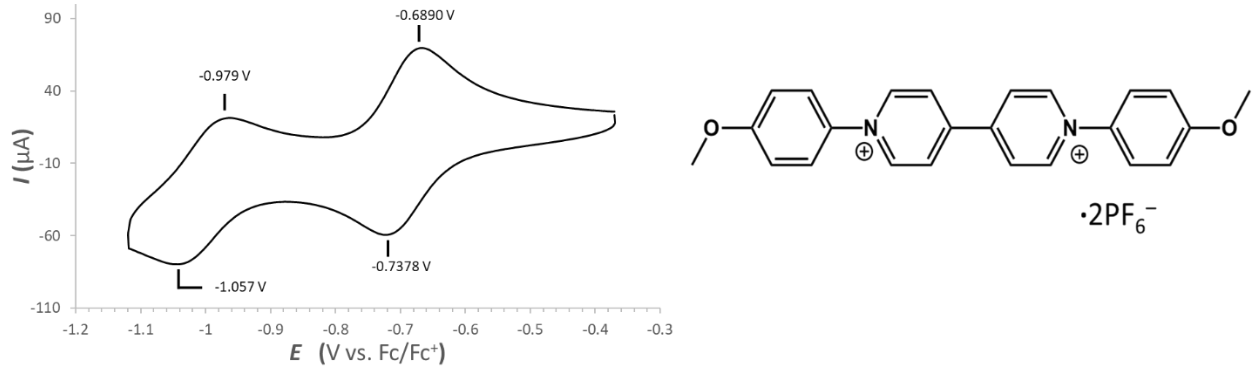

Figure S53. Cyclic voltammogram of methoxyphenyl viologen (1 mM) recorded in DMF at a scan rate of 2 V/s.

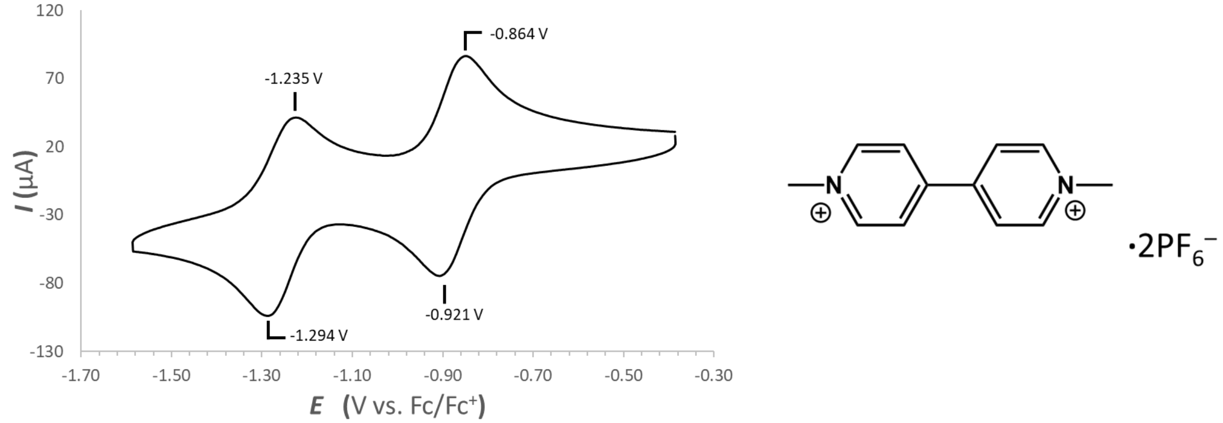

Figure S54. Cyclic voltammogram of methyl viologen (1 mM) recorded in DMF at a scan rate of 2 V/s.

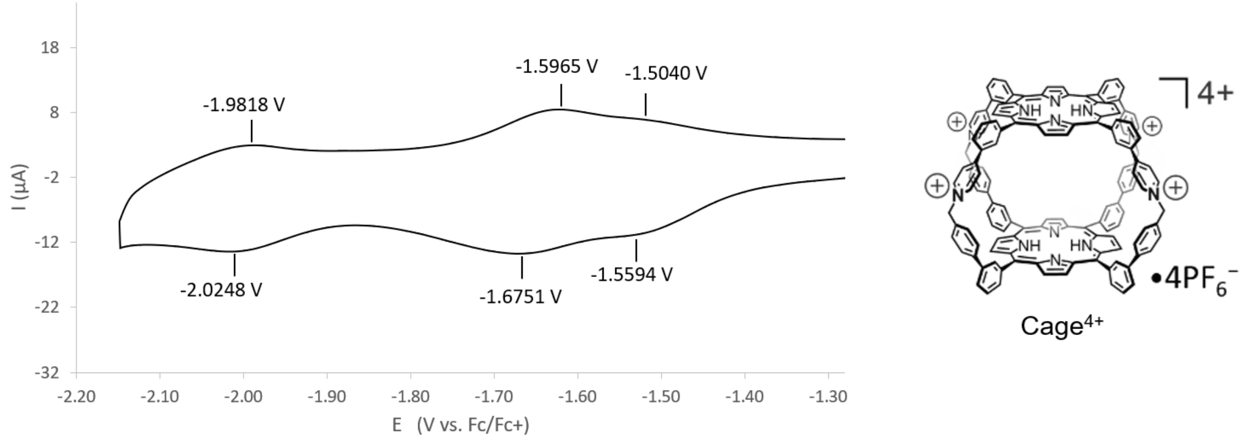

Figure S55: Cyclic voltammogram of **Cage**•4PF$_6$ (0.1 mM) recorded at a scan rate of 1 V/s in benzonitrile. Note that the porphyrin and pyridinium centered reductions are better separated than was observed for experiments performed in DMF.

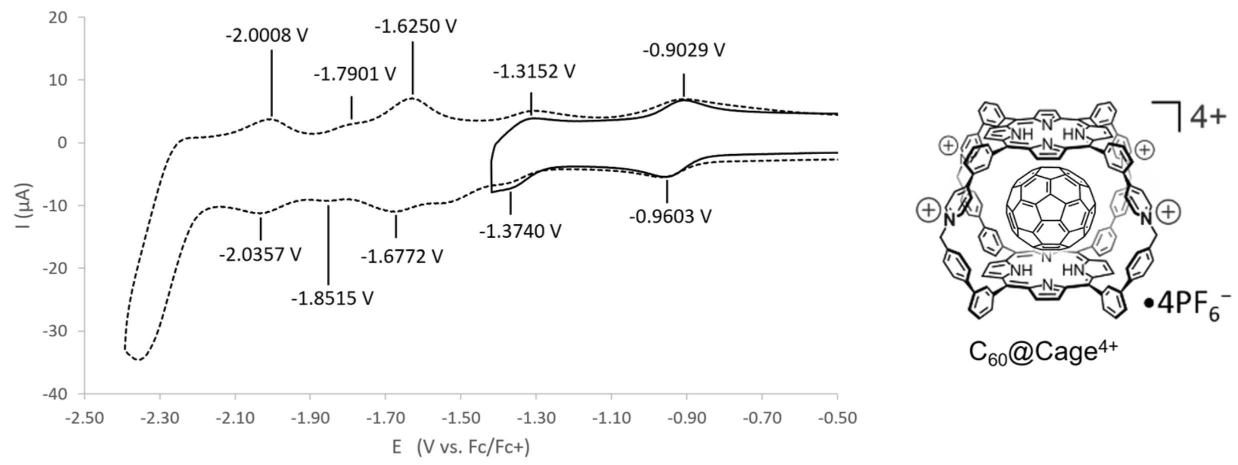

Figure S56. Cyclic voltammogram of **[C_{60}@Cage]**•4PF_6 (0.1 mM) in benzonitrile recorded at a scan rate of 1 V/s. Note that the first two fullerene-centered reductions show good reversibility, and the $C_{60}^{1-/2-}$ couple is better defined than was observed using DMF as the solvent.

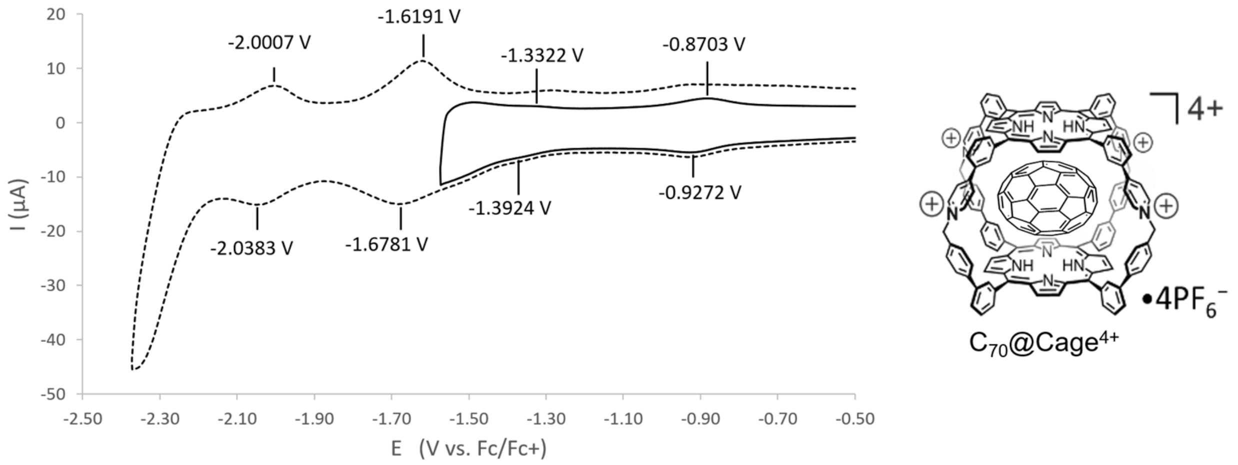

Figure S57. Cyclic voltammogram of **[C_{70}@Cage]**•4PF_6 (0.1 mM) in benzonitrile recorded at a scan rate of 1 V/s. As seen for data collected in DMF, the first fullerene-centered reduction shows good reversibility within a potential window that avoids reduction of **Cage^{4+}**. However, the second fullerene-centered reduction is less well-defined than seen in DMF, even when reduction of **Cage^{4+}** is avoided.

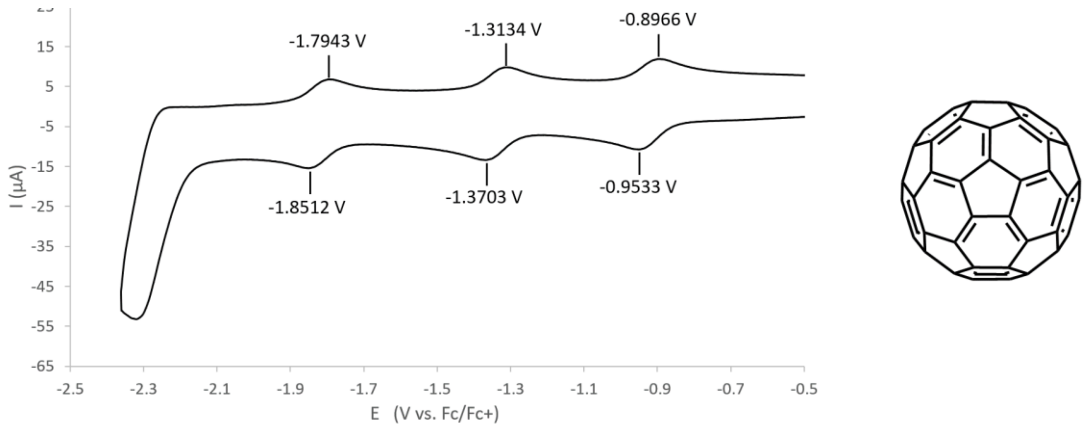

Figure S58. Cyclic voltammogram of C_{60} (0.1 mM) recorded at a scan rate of 1V/s in benzonitrile.

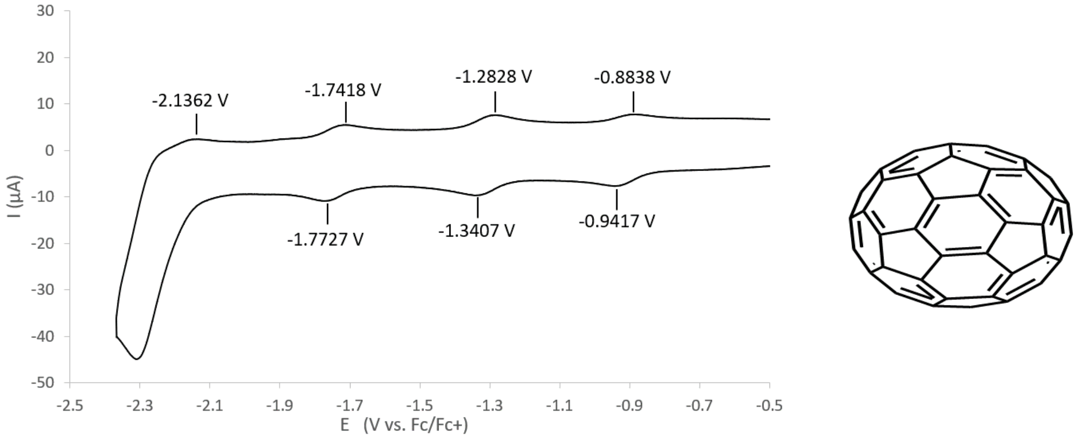

Figure S59. Cyclic voltammogram of C_{70} (0.1 mM) recorded at a scan rate of 1V/s in benzonitrile.

	**1st Fullerene Reduction**	**2nd Fullerene Reduction**	**Pyridinium-Linker Reduction**	**3rd Fullerene Reduction**	**2nd Cage Reduction**
C_{60}	-0.93 V	-1.34 V	—	-1.82 V	—
C_{70}	-0.91 V	-1.31 V	—	-1.76 V	—
**C_{60}@Cage^{4+}**	-0.93 V	-1.34 V	-1.65 V	-1.82 V	-2.02 V
**C_{70}@Cage^{4+}**	-0.90 V	-1.36 V	-1.65 V	Not obs.	-2.02 V
**Cage^{4+}**	—	—	-1.64 V	—	-2.00 V

Table S1. Comparison of redox potentials for free fullerene, **Cage^{4+}**, and host-guest complexes in benzonitrile. Encapsulation within **Cage^{4+}** minimally perturbs the redox potentials of both fullerenes. Similarly, the fullerenes do not have a significant impact on the cage-centered reduction events. These small changes in potentials in both DMF and PhCN suggests solvents do not play a significant role in mediating the electrostatic interaction between reduced fullerenes and **Cage^{4+}**.

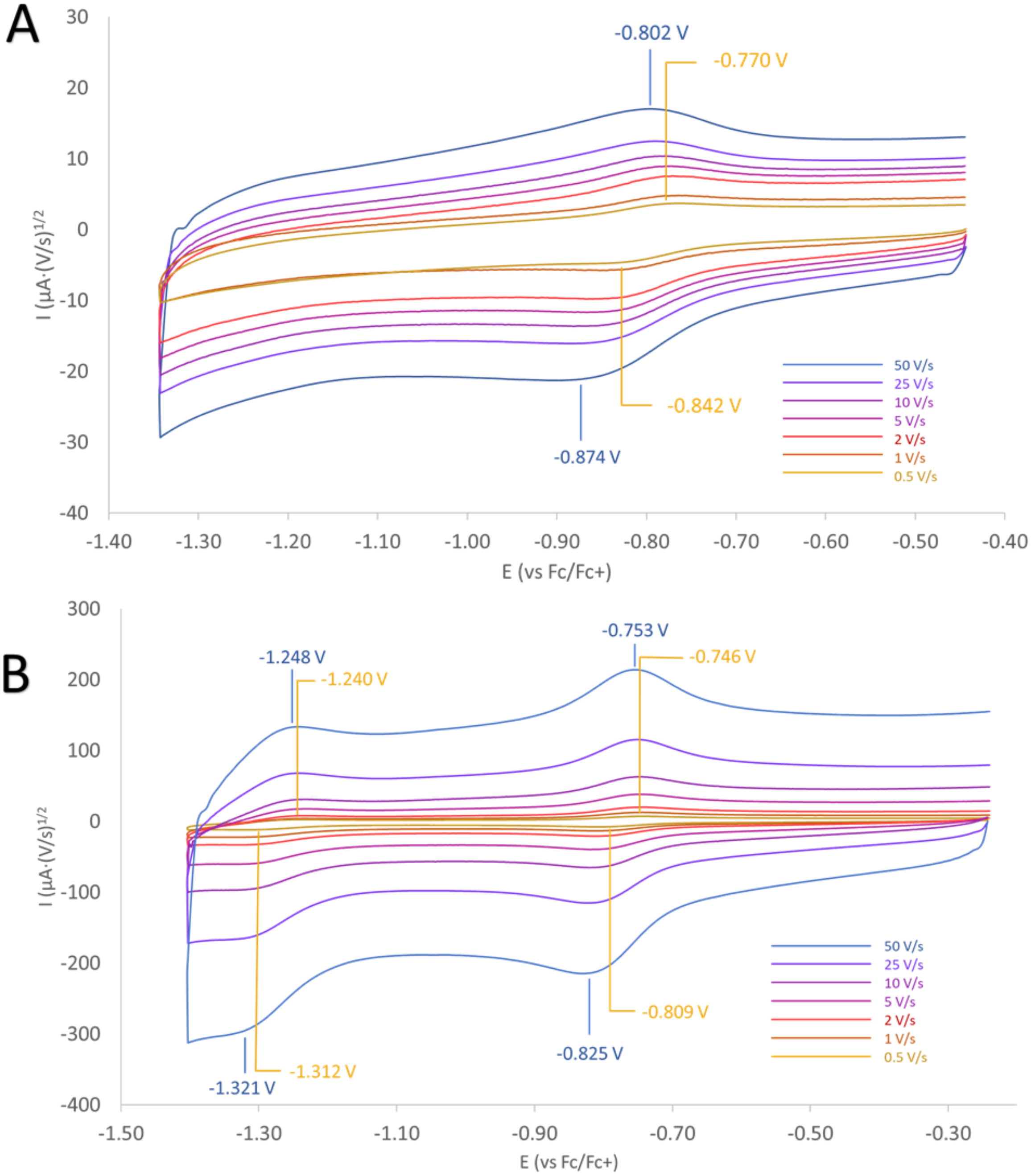

Figure S60. Cyclic voltammogram displaying (A) the first fullerene-centered reduction of **C_{60}@Cage^{4+}** (0.1 mM), and (B) the first two fullerene-centered reductions of **C_{70}@Cage^{4+}** (0.1 mM) in DMF at scan rates from 0.5 to 50 V s^{-1}.

6. UV-vis-NIR Spectroscopy

UV-vis-NIR spectra were recorded at room temperature using a Shimadzu UV-2600i spectrophotometer equipped with a an ISR-2600Plus integrating sphere detector that provides a 220 – 1400 nm wavelength range for solution absorbance measurements. Samples for UV-vis-NIR spectroscopy were prepared at an analyte concentration of 0.125 mM and measured in 1 mm path length quartz cuvettes.

For experiments examining complexes of fulleride anions bound in **$Cage^{4+}$**, the fulleride@**$Cage^{4+}$** complexes were prepared in situ from deoxygenated solutions of fullerene@**$Cage^{4+}$** by addition of $Cp^*{}_2Co$ as a stock solution (160 mM) in C_6D_6.

For assessing the ability of different strength reducing agents to reduce encapsulated C_{60}, two viologens, methyl viologen (MV^{2+}) and methoxyphenyl viologen (MPV^{2+}), were treated with Cp_2Co to generate the radical-cation states of the viologens, which were then examined as chemical reductants for accessing the first fullerene-centered reduction of **C_{60}@$Cage^{4+}$**. Stock solutions of each viologen radical cation were prepared via the addition of Cp_2Co in benzene (27.8 M) to viologen solutions in DMF (7.6 mM for MPV^{2+}, 15.1 mM for MV^{2+}). To avoid over-reduction of the viologens and ensure no unreduced Cp_2Co remained in solution, 0.8 equiv of Cp_2Co was used for each viologen. Reduced viologen solutions were then added to solutions of **C_{60}@$Cage^{4+}$** in DMF (0.125mM) in order to introduce 1 equiv of reduced viologen per host-guest complex. Cuvettes sealed under a nitrogen atmosphere were used to perform UV-vis absorbance experiments to monitor the reduction of the fullerene.

To assess the association of C_{60}^{2-} and C_{70}^{2-} in the reduced **$Cage^{2-}$** state of the host, 0.50 mM solutions of **C_{60}@$Cage^{4+}$** and **C_{70}@$Cage^{4+}$** in MeCN were treated with 8 equiv of $Cp^*{}_2Co$ that was added as a stock solution (170 mM) in benzene in an N_2 atmosphere glovebox. For both complexes, a precipitate formed immediately upon adding the reductant and the resulting suspension was stirred for 15 min before filtering through a 0.22 micron PTFE filter into a quartz cuvette. The samples were sealed under N_2 with a PTFE stopper and UV-vis-NIR spectra were measured immediately after removing the cuvettes from the glovebox. The spectra confirmed complete disappearance of signals corresponding to the host, enabling quantification of the amount of free C_{60}^{2-} or C_{70}^{2-} remaining in solution.

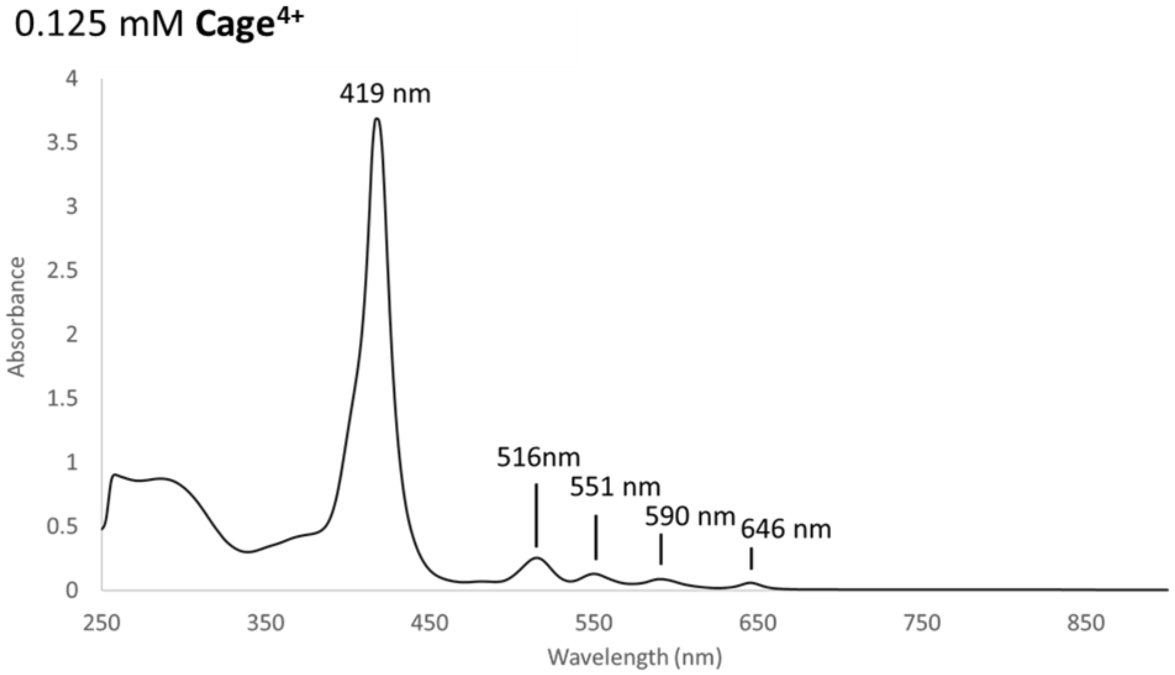

Figure S61. UV-vis-NIR spectra of **Cage**•4PF_6 (0.125 mM) in DMF.

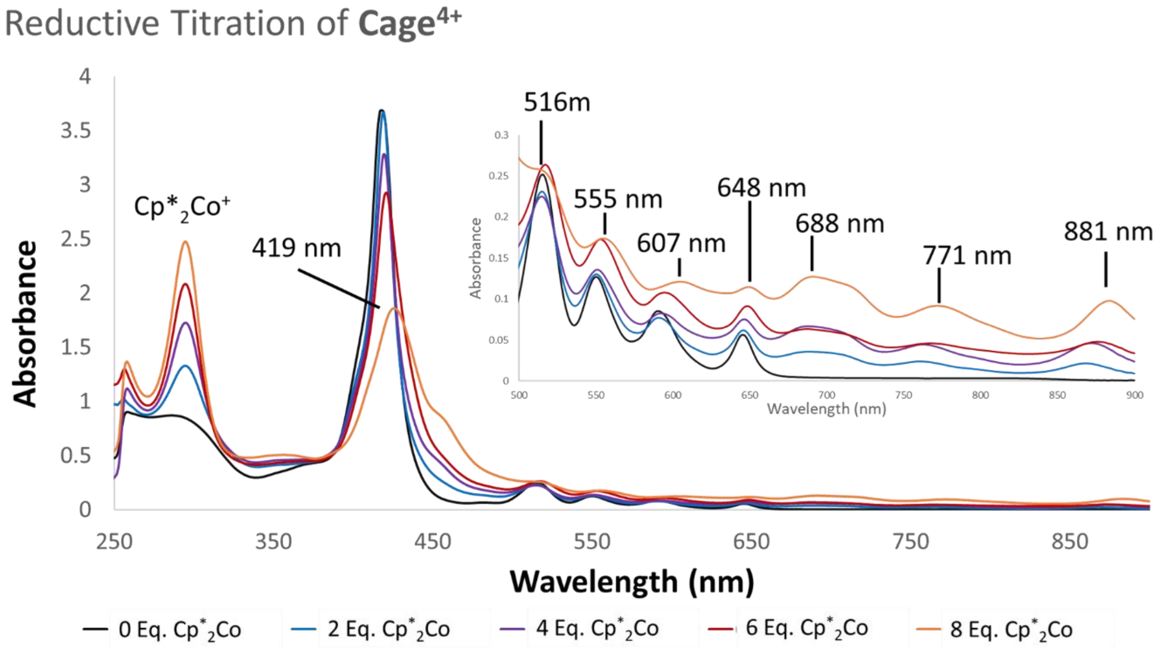

Figure S62. UV-vis-NIR spectra of **Cage**•4PF_6 (0.125 mM) upon addition of 0 – 8 equiv of Cp^*_2Co in 2 equiv increments. The Soret band and q-peaks of the fully reduced **Cage**$^{4-}$ are labelled, as is the absorbance of Cp^*Co^+ produced as a byproduct of the Cp^*_2Co reductant

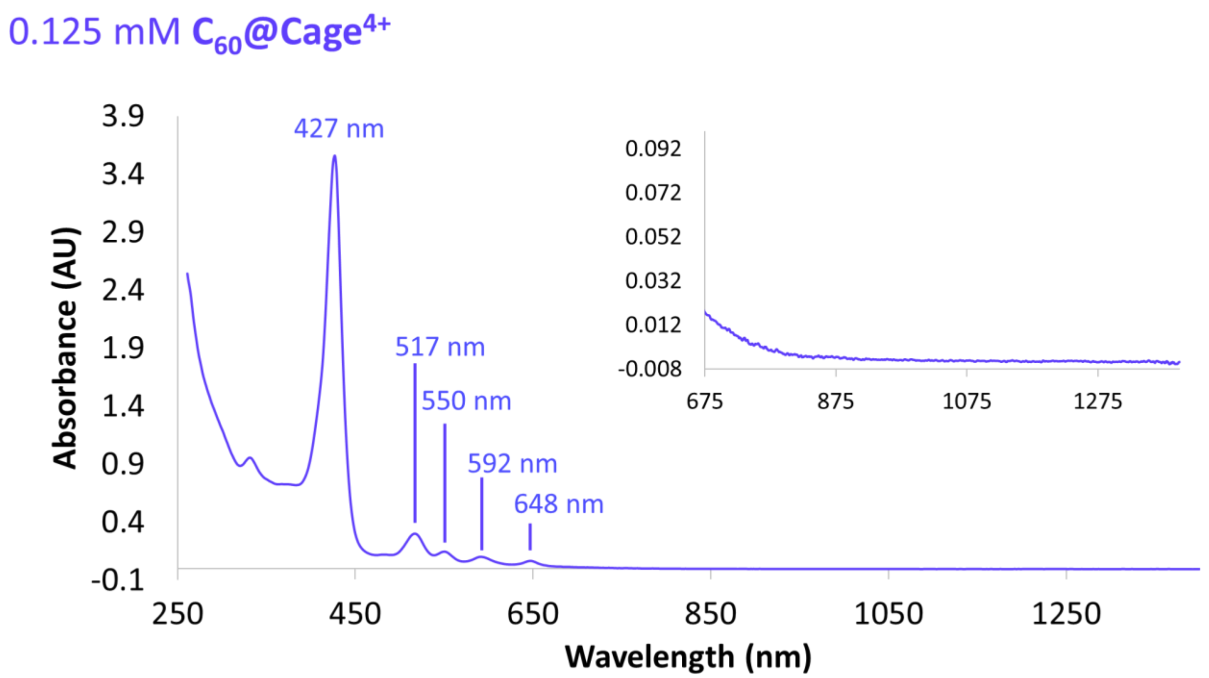

Figure S63. UV-vis-NIR spectrum of **[C_{60}@Cage]**•4PF_6 (0.125 mM).

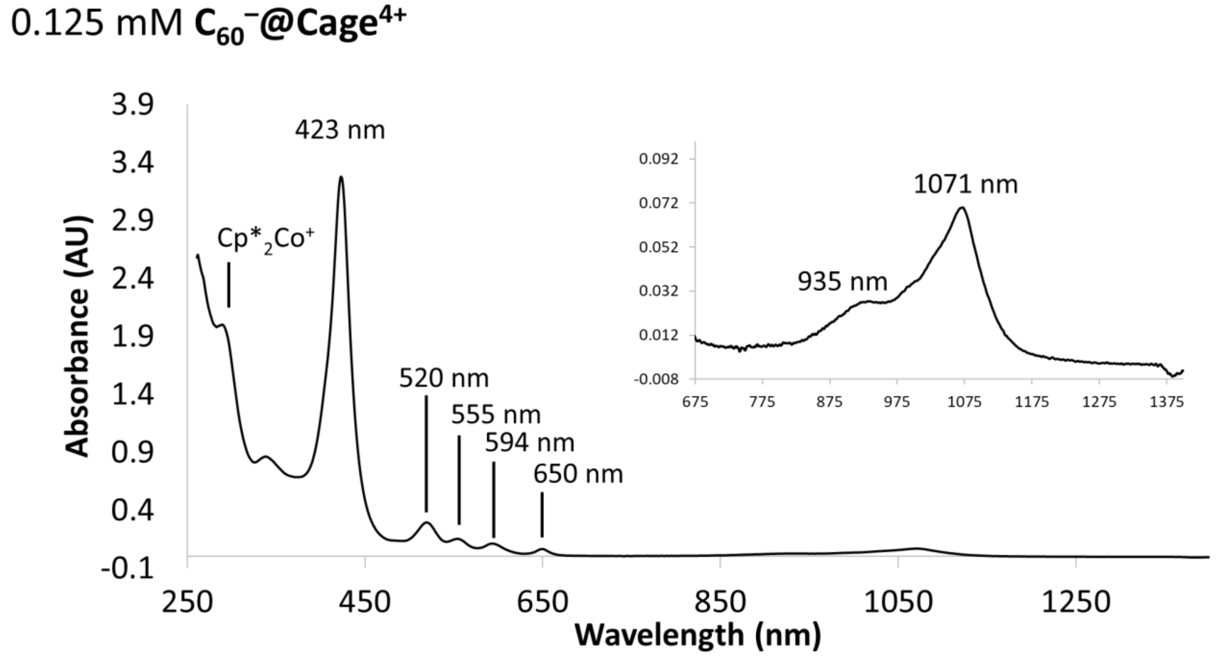

Figure S64. UV-vis-NIR spectrum of **C_{60}^{-}@Cage^{4+}** (0.125 mM) generated by addition of 1 equiv Cp*$_2$Co to a solution of **[C_{60}@Cage]**•4PF_6 in DMF. The inset shows absorbances attributed to the C_{60}^{-} anion.

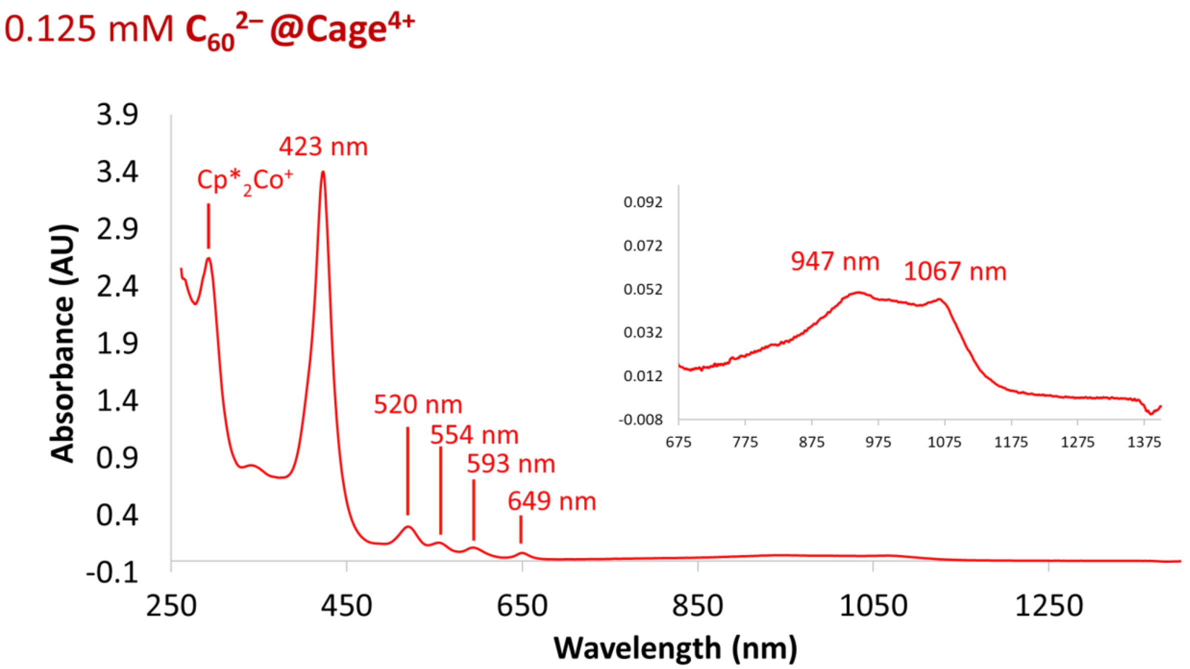

Figure S65. UV-vis-NIR spectrum of **C_{60}^{2-}@Cage^{4+}** (0.125 mM) generated by addition of 2 equiv Cp*$_2$Co to a solution of **[C_{60}@Cage]**•4PF_6 in DMF. The inset shows absorbances attributed to the C_{60}^{2-} dianion.

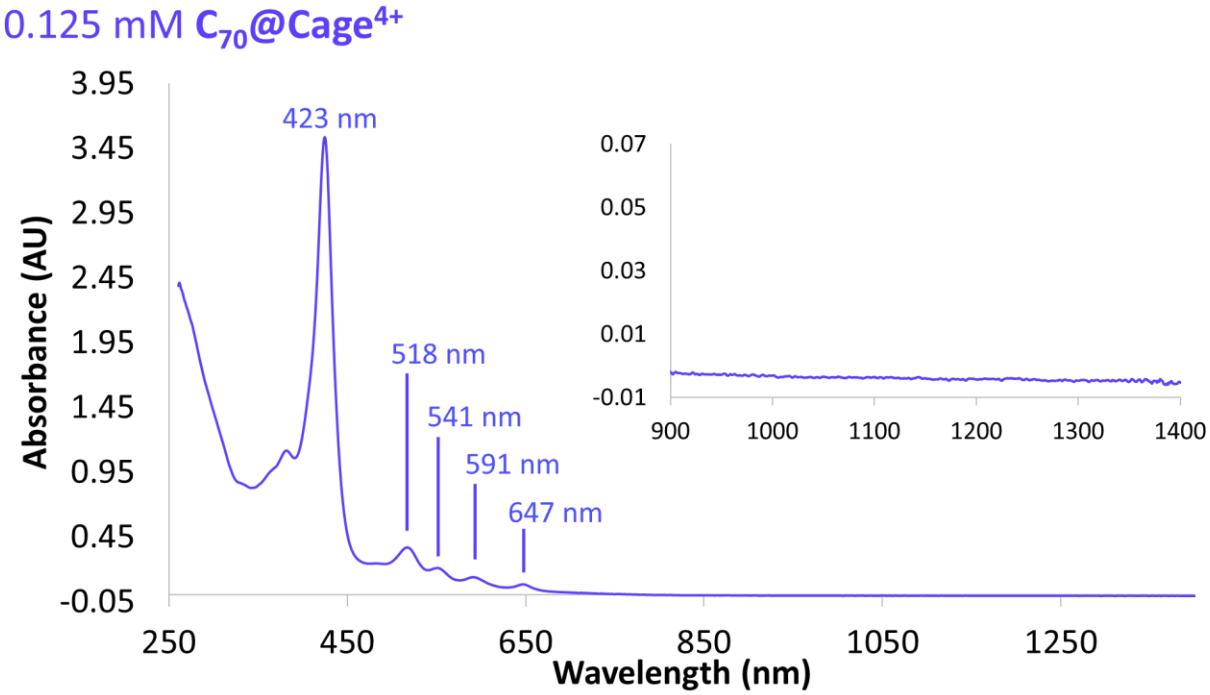

Figure S66. UV-vis spectrum of **[C_{70}@Cage]**•4PF_6 (0.125 mM) in DMF.

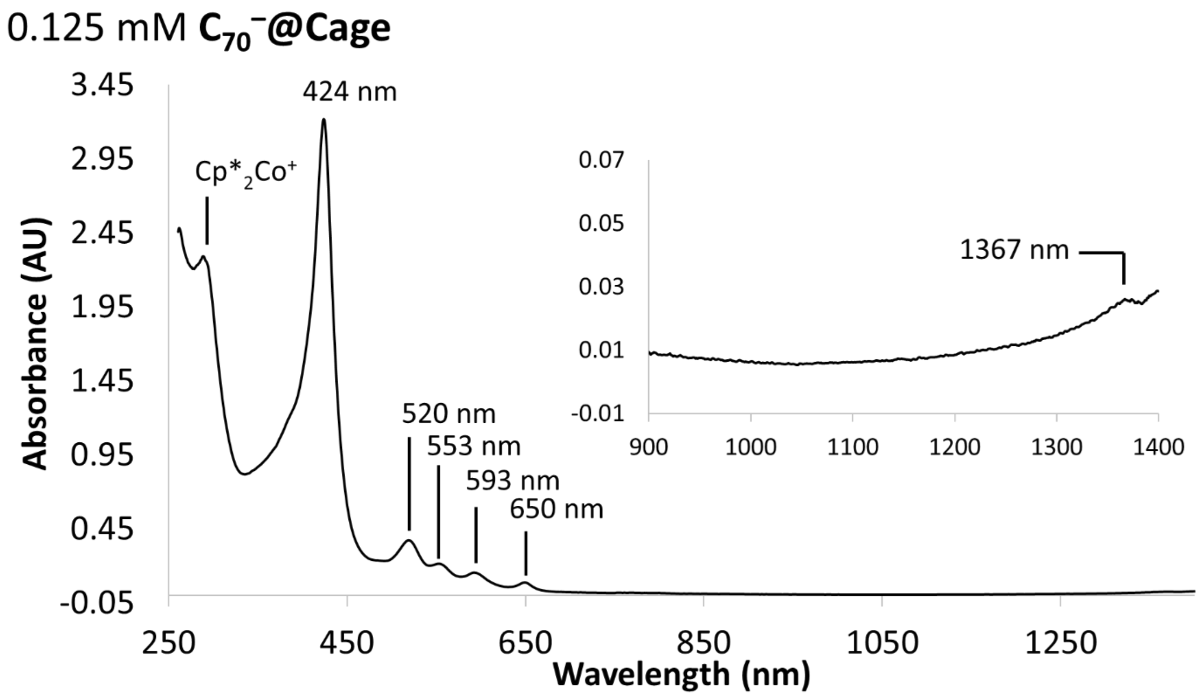

Figure S67. UV-vis-NIR spectrum of **C_{70}^{-}@Cage^{4+}** (0.125 mM) generated by addition of 1 equiv Cp^*_2Co to a solution of **[C_{70}@Cage]**•$4PF_6$ in DMF. The inset shows absorbances attributed to the C_{70}^{-} anion.

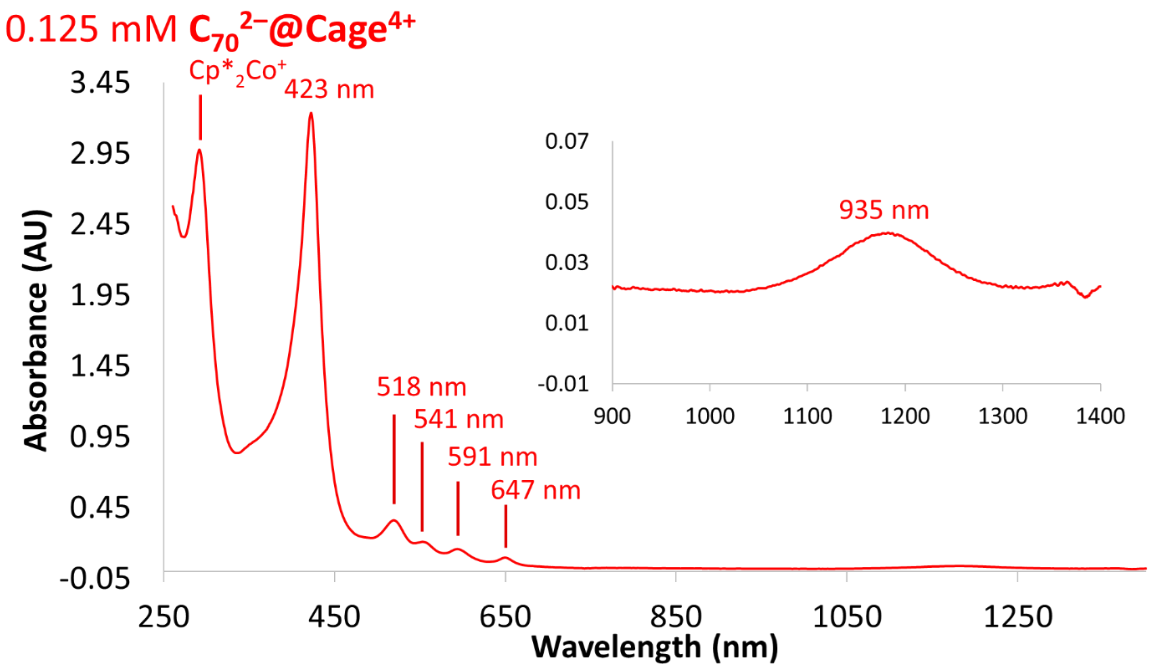

Figure S68. UV-vis-NIR spectrum of **C_{70}^{2-}@Cage^{4+}** (0.125 mM) generated by addition of 2 equiv Cp^*_2Co to a solution of **[C_{70}@Cage]**•$4PF_6$ in DMF. The inset shows absorbances attributed to the C_{70}^{2-} anion.

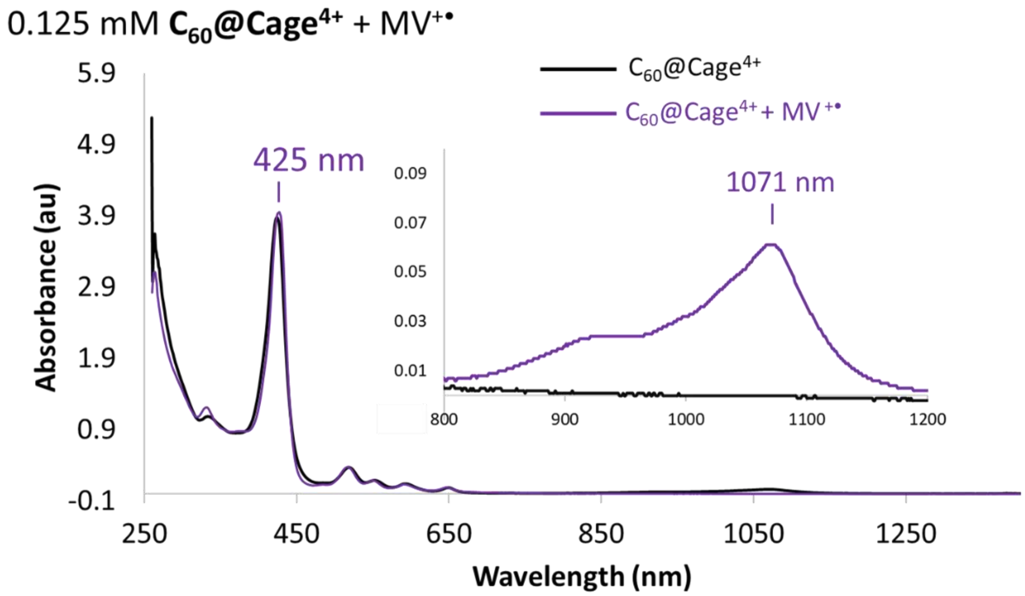

Figure S69. UV-vis-NIR spectra of a solution of **[C_{60}@Cage]**•4PF_6 before and after treatment with 1 equiv of the methyl viologen radical cation ($MV^{+•}$), which has a reducing strength of ca. −0.89 V vs. $Fc^{+/0}$ in DMF as determined by examining the $MV^{2+}/MV^{+•}$ redox couple by cyclic voltammetry (see Figure S54).

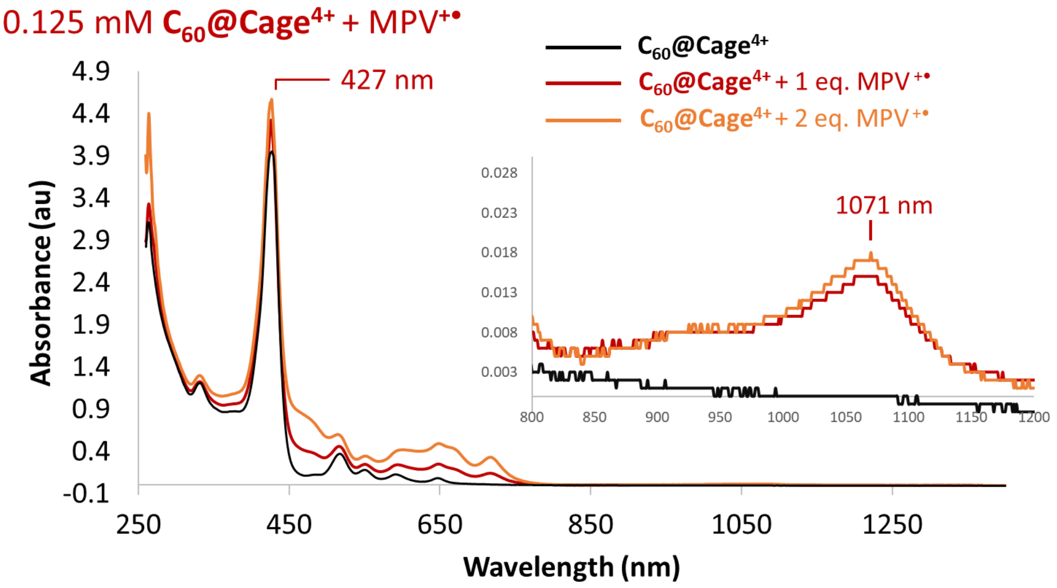

Figure S70. UV-vis-NIR spectra of a solution of **[C_{60}@Cage]**•4PF_6 before and after treatment with 1 and 2 equiv of the methoxyphenyl viologen radical cation ($MPV^{+•}$), which has a reducing strength of ca. −0.71 V vs. $Fc^{+/0}$ in DMF as determined by examining the $MPV^{2+}/MPV^{+•}$ redox couple by cyclic voltammetry (see Figure S53). Partial reduction of the C_{60} guest is evident from the NIR absorption band with l_{max} = 1071 nm, but the intensity of this absorption is much lower than that observed upon reduction of **C_{60}@Cage^{4+}** with Cp^*_2Co or $MV^{+•}$ (see Figures S58 and S63), confirming that the encapsulated guest has a reduction potential that is not perturbed much from that of unencapsulated C_{60} (-0.82 V vs. $Fc^{+/0}$ in DMF).

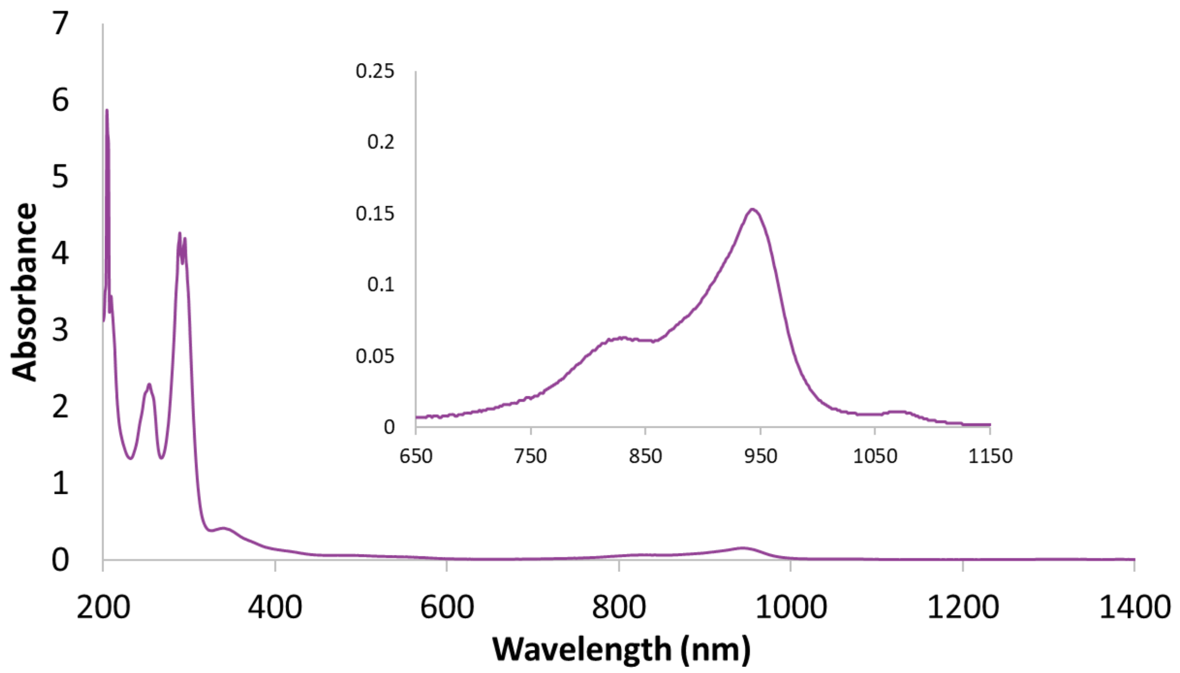

Figure S71. UV-Vis-NIR spectrum of C_{60}^{2-} remaining in solution after adding 8 equiv of Cp^*_2Co to a solution of **C_{60}@Cage^{4+}** (0.50 mM) in MeCN. A 0.11 mM concentration of the fulleride was determined based on the absorption at λ_{max} = 944 nm (ε = 14,000 M^{-1}), suggesting 22% of the fullerene remained in solution.

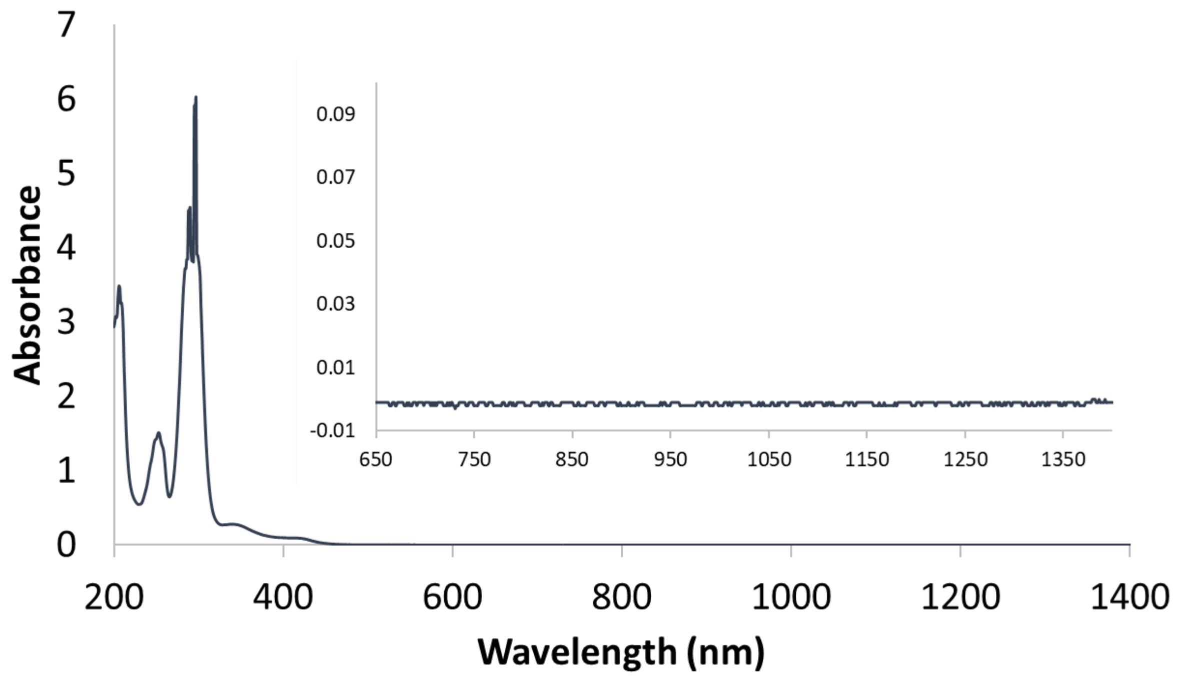

Figure S72. UV-vis-NIR spectrum of a 0.50 mM solution of **C_{70}@Cage^{4+}** (0.50 mM) in MeCN after the addition of 8 equiv of Cp^*_2Co. The absence of any signals corresponding to the reduced host or fulleride anions indicates that the fulleride remains bound in the reduced host upon its precipitation from solution.

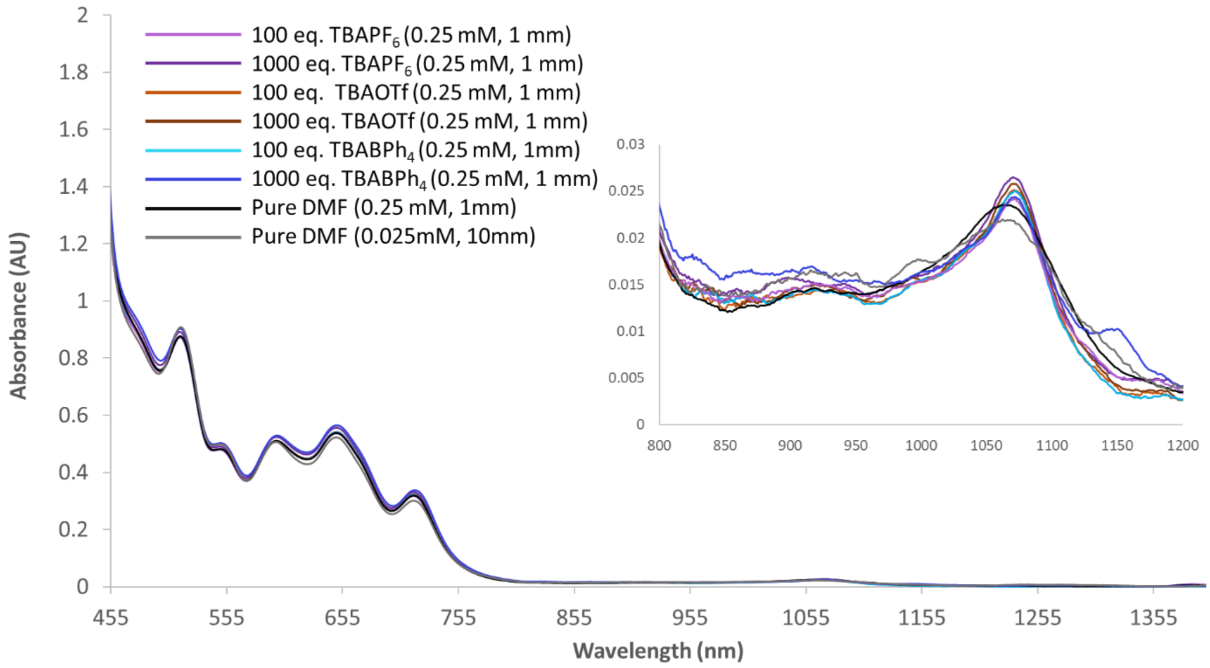

Figure S73. Overlaid absorbance spectra of solutions of **C_{60}@Cage^{4+}** in DMF after treatment with 1 equiv of the methoxyphenyl viologen radical cation (MPV$^{•+}$, $E_{1/2}$ = –0.71 V vs Fc$^{+/0}$) to establish a small equilibrium concentration of the reduced complex **C_{60}^{-}@Cage^{4+}**. Spectra were acquired at different concentrations of **C_{60}@Cage^{4+}** and with various concentrations of electrolytes added. The NIR absorption band (see inset) of the C_{60}^{-} guest indicates similar ratios of **C_{60}^{-}@Cage^{4+}** to **C_{60}@Cage^{4+}** were formed in each sample, suggesting that ions in solution have little impact on the reduction potential of the encapsulated guest.

7. Fluorescence measurements of C_{60} and C_{70} association in Cage^{4+}

Scintillation vials were treated to silylate exposed surface OH sites by leaving the vials in an evacuated desiccator with 10 mL of hexamethyldisilazane for at least 30 minutes before use. The vials were then filled with benzonitrile, 25.0 μL of a 4.0 x 10^{-5} M solution of **Cage**•4PF_6 in benzonitrile, and appropriate amounts of a stock solutions of 4.16 x 10^{-5} M C_{60} in benzonitrile for a total volume of 5 mL per sample, a **Cage^{4+}** concentration of 2.0 x 10^{-7} M, and C_{60} concentrations ranging from 1.0 x 10^{-7} M to 9.0 x 10^{-6} M. Samples with no C_{60} were prepared as references. All vials were sealed with parafilm and sonicated in a Bransonic CPX2800H ultrasonic bath for 1 h. The fluorescence emissions of each sample were then promptly measured using a Horiba Aqualog combination spectrophotometer-fluorimeter. Absorbances were also measured to ensure that they did not exceed an absorbance of 0.1. The same procedure was followed for C_{70}, except that lower concentration stock solutions were used, producing final solutions withC_{70} concentrations ranging from 5.0 x 10^{-9} M to 1.5 x 10^{-7} M and a calculated **Cage^{4+}** concentration of 3.98 x 10^{-8} M. Repeat data points around the inflection point were taken to help define the shape of the curve.

The concentration of host-guest complex in each sample was calculated according to Eq S1, where $[H]_0$ is the total host (**Cage^{4+}**) concentration, I is the measured reduction of fluorescence intensity, and I_0 is the fluorescence intensity of a sample of the host with no guest (fullerene) present. Since small amounts of **Cage^{4+}** were found to adsorb on the surface of the glass vials used for sample preparation, the value of $[H]_0$ was determined by comparing the fluorescence of the sample of **Cage^{4+}** without added guest vs. the fluorescence intensity of freshly prepared samples of **Cage^{4+}**. The concentrations of host-guest complex were then plotted as the dependent variable against the added guest concentration as the independent variable. OriginLab 2020 was used for baseline correction and fitting the resulting plots to the equations outlined by Thordarson[10] (Eq S2) for binding of a host and guest with a 1:1 stoichiometry.

$$[Complex] = [H]_0 * \left(1 - \frac{I}{I_0}\right) \qquad \text{Eq S1}$$

$$[Complex] = \frac{1}{2}\left(\left([H]_0 + [G]_0 + \frac{1}{K_{eq}}\right) - \sqrt{\left([H]_0 + [G]_0 + \frac{1}{K_{eq}}\right)^2 - 4[H]_0[G]_0}\right) \qquad \text{Eq S2}$$

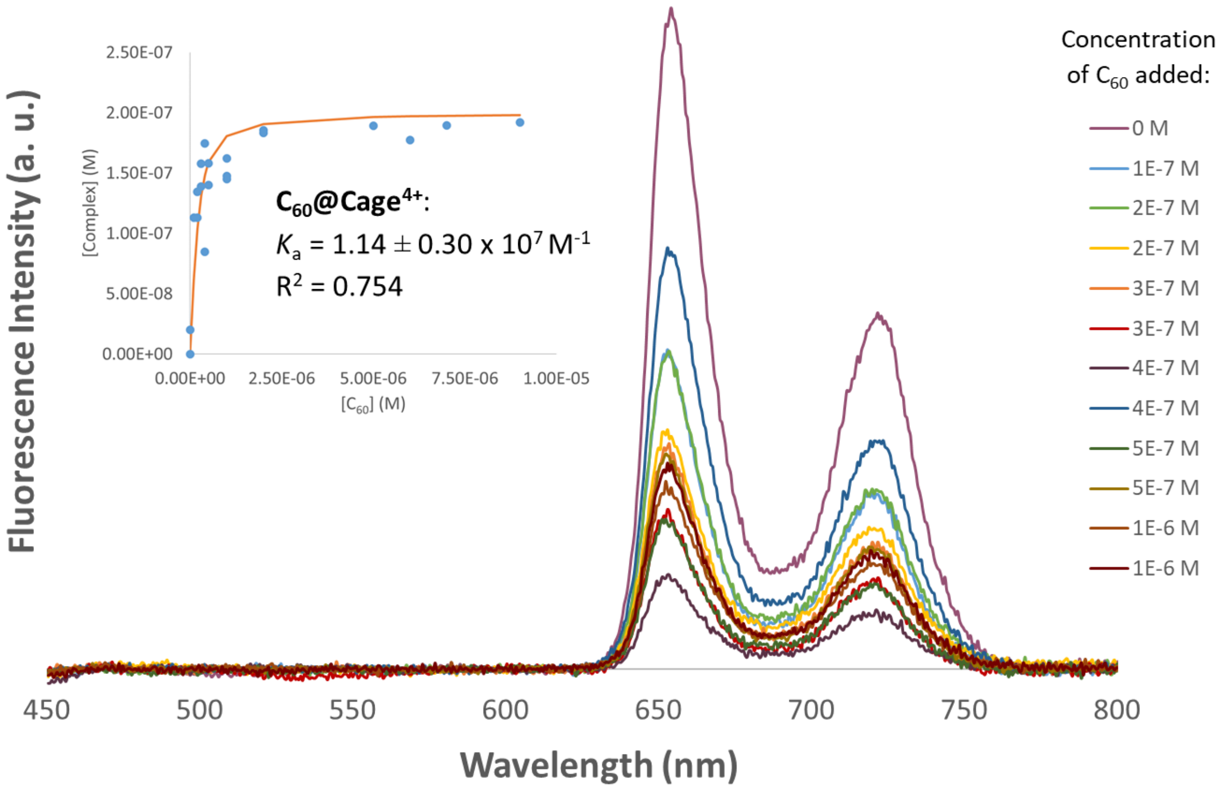

Figure S74. Representative fluorescence spectra showing the decrease in fluorescence intensity of benzonitrile solutions of **Cage^{4+}** containing various amounts of added C_{60}. The inset displays the resultant curve from fitting Eq S2 to the collated data from two runs of sample preparation and data collection.

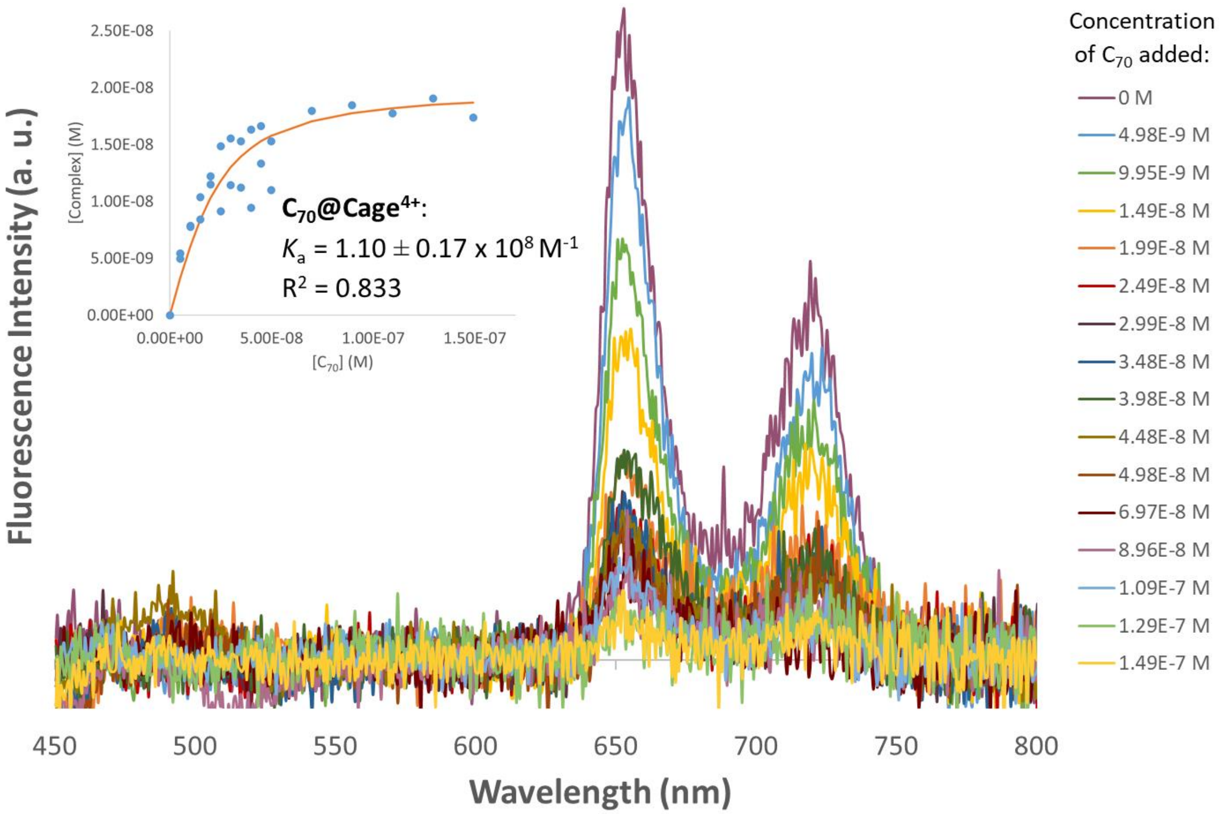

Figure S75. Representative fluorescence spectra showing the decrease in fluorescence intensity of benzonitrile solutions of **Cage**$^{4+}$ containing various amounts of added C_{70}. The inset displays the resultant curve from fitting Eq S2 to the collated data from two runs of sample preparation and data collection.

8. DFT Calculations

All density functional theory calculations were performed using Gaussian 16 revision A.03. The b3lyp functional was employed in all calculations along with the SMD solvation model of Truhlar and Cramer.[11] For optimizing the geometry of **Cage**$^{4+}$, the 6-31+G(d,p) basis set was employed for all atoms (C,N,H), and the structure was optimized from an initial guess based on reasonable bond lengths and angles for the porphyrin units and benzylpyridinium linkers. The optimized geometry of **Cage**$^{4+}$ was used along with reported structures of C_{60} and C_{70} to construct starting points for optimizations of the host-guest complexes **C_{60}@Cage**$^{4+}$ and **C_{70}@Cage**$^{4+}$. The host-guest complexes were optimized using the 3-21+G basis set because calculations could not be completed in a reasonable time (≤30 days) using a larger basis set.

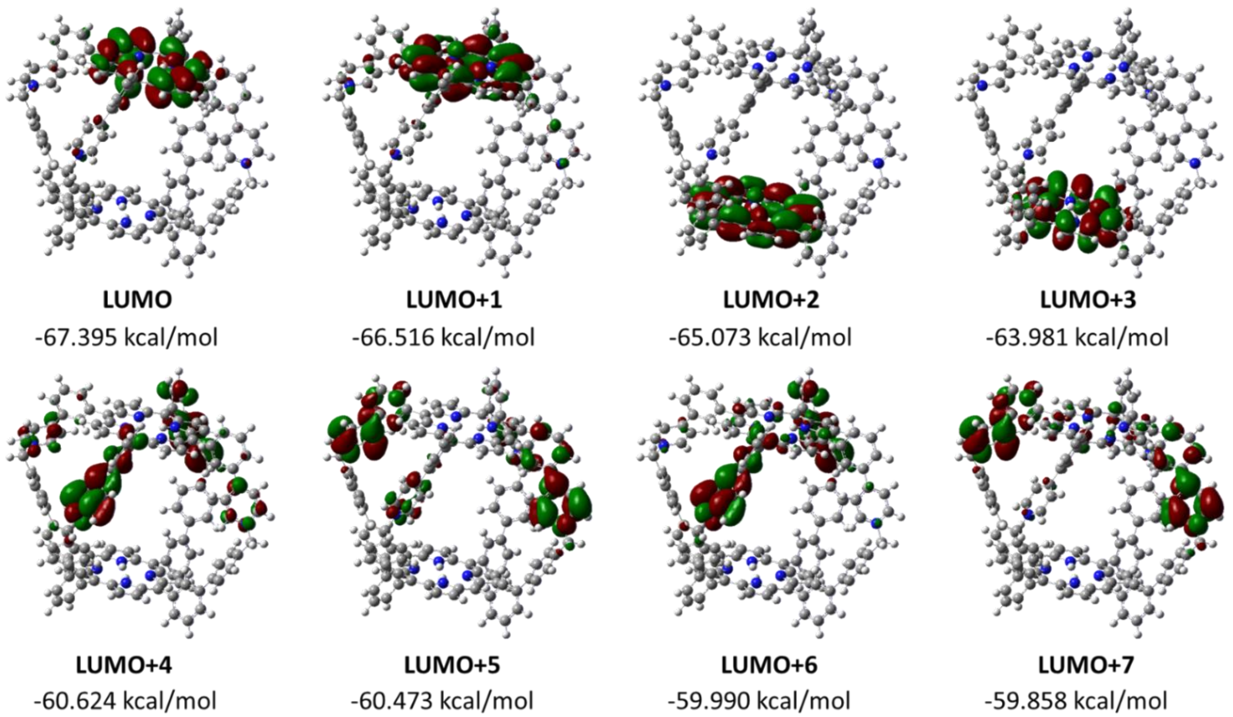

Figure S76. Depictions of the LUMO to LUMO+7 orbitals of **Cage^{4+}** determined via DFT calculations. Orbital images were generated using GaussView at a surface isovalue of 0.02.

9. References

1. P. D. W. Boyd, P. P. Bhyrappa, J. Stinchcombe, R. Bolskar, Y. Sun, C. A. Reed *J. Am. Chem. Soc.* 1995, **117**, 2907-2914.
2. R. J. Abraham, I. Marsden, L. Xiujing, *Magn. Reson. Chem.,* 1990, **28**, 1051-1057.
3. J. M. Guilbaud, A. Labonde, A. Selmi, M. Kammoun, H. Cattey, N. Pirio, J. Roger, J. C. Hierso *Catal. Commun.* 2018, **111**, 52-58.
4. S. J. Nemat, H. Jedrzejewska, A. Prescimone, A. Szumna, K. Tiefenbacher, K. *Org. Lett.* 2020, **22**, 5506-5510.
5. L. Chen, F. Hartl, H. M. Colquhoun, W. Barnaby, *Tetrahedron Lett.* 2017, **58**, 1859-1862.
6. I. Fenger, C. Drian, *Tetrahedron Lett.* 1998, **39**, 4287-4290.
7. N. P. Vargo, J. B. Harland, B. W. Musselman, N. Lehnert, M. Z. Ertem, J. R. Robinson, *Angew. Chem., Int. Ed. Engl.* 2021, **60**, 19836-19842.
8. P. K. Pal, S. Chowdhury, M. G. B. Drew, D. Datta, *New J. Chem.* 2002, **26,** 367-371.
9. D. Dubois, G. Moninot, W. Kutner, M.T. Jones, K.M. Kadish, *J. Phys. Chem.* 1992, **96**, 7137-7145.
10. P. Thordarson, *Chem. Soc. Rev.* **2011**, 40, 1305-1323.
11. A. V. Marenich, C. J. Cramer, D. G. Truhler, *J. Phys. Chem. B* 2009, **113**, 6378 – 6396.
